# Discovery of a 4-Hydroxy-3′-Trifluoromethoxy-Substituted Resveratrol Derivative as an Anti-Aging Agent

**DOI:** 10.3390/molecules29010086

**Published:** 2023-12-22

**Authors:** Yinhu Liang, Xi Chen, Zhifeng Teng, Xuekun Wang, Jie Yang, Guoyun Liu

**Affiliations:** 1 School of Pharmaceutical Sciences, Liaocheng University, 1 Hunan Street, Liaocheng 252059, Chinaxuekunwang0610@126.com (X.W.); 2 Liaocheng Key Laboratory of Quality Control and Pharmacodynamic Evaluation of Ganoderma Lucidum, Liaocheng University, 1 Hunan Street, Liaocheng 252059, China

**Keywords:** resveratrol derivatives, anti-aging, oxidative stress, inflammation, apoptosis, D-galactose

## Abstract

With the intensification of population aging, aging-related diseases are attracting more and more attention, thus, the study of aging mechanisms and anti-aging drugs is becoming increasingly urgent. Resveratrol is a potential candidate as an anti-aging agent, but its low bioavailability limits its application in vivo. In this work, a 4-hydroxy-3′-trifluoromethoxy-substituted resveratrol derivative (**4–6**), owing to its superior cell accumulation, could inhibit NO production in an inflammatory cell model, inhibit oxidative cytotoxicity, and reduce ROS accumulation and the population of apoptotic cells in an oxidative stress cell model. In D-galactose (D-gal)-stimulated aging mice, **4–6** could reverse liver and kidney damage; protect the serum, brain, and liver against oxidative stress; and increase the body’s immunity in the spleen. Further D-gal-induced brain aging studies showed that **4–6** could improve the pathological changes in the hippocampus and the dysfunction of the cholinergic system. Moreover, protein expression related to aging, oxidative stress, and apoptosis in the brain tissue homogenate measured via Western blotting also showed that **4–6** could ameliorate brain aging by protecting against oxidative stress and reducing apoptosis. This work revealed that *meta*-trifluoromethoxy substituted **4–6** deserved to be further investigated as an effective anti-aging candidate drug.

## 1. Introduction

Aging is a complex natural process, characterized by the degeneration of function and structure, as well as the decrease in resistance and adaptability. Due to the increase in the population and the extension of lifespan, the aging population is increasing, and aging-related diseases have attracted great attention worldwide, therefore, exploring the mechanisms of aging and developing anti-aging drugs have become increasingly important.

The aging process relates to many complex factors, including multiple genetic and environmental factors and diet. Cumulative research has shown that reactive oxygen species (ROS) caused by oxidative stress play an important part in aging [1,2,3]. Excessive accumulation of ROS can induce oxidative stress and disrupt the structure of proteins, phospholipids, and DNA, even leading to damage in cells and tissues [4]. Therefore, resisting oxidative stress or inhibiting the production of ROS is an effective prevention or treatment strategy for preventing age-related diseases, especially neurodegenerative diseases [5,6,7]. In addition, inflammation is believed to play an important part in the aging process [8,9]. Chronic inflammation is common in aging and age-related diseases. Accumulating evidence indicates that aging is accompanied by a low-grade chronic inflammatory state and can be explained by the imbalance between anti-inflammatory and pro-inflammatory factors [10].

Resveratrol, a stilbene derivative, is a natural nutrient in grapes, which possesses many biological characteristics, such as anti-inflammatory, neuroprotective, anti-cancer, anti-oxidative, and anti-diabetic effects [11,12,13]. In recent years, the anti-aging potential of resveratrol has been one of the most eye-catching biological activities [14,15,16,17,18,19,20,21,22]. Resveratrol is a potential candidate as an anti-aging agent, which can exert its anti-aging and health benefits via anti-oxidative, calorie-restrictive, and anti-inflammatory effects through activating the nuclear factor erythroid 2-related factor 2 (Nrf2), sirtuin 1 (Sirt1), and AMP-activated protein kinase (AMPK) signaling pathways. However, resveratrol demonstrates low aqueous solubility and rapid metabolism in vivo [23,24], which limits its bioavailability and may also be the major obstacle in translating its effects to humans. Therefore, various derivatives have been studied to overcome these limitations. For example, fluorinated substance **1–4** (3,5-dihydroxy-4′-trifluoromethoxy-*trans*-stilbene) showed excellent stability and cell uptake ability in A549 cells. However, **1–4** induced obvious premature senescence and caused a clear block in cells in the G1 phase (81.5%), so as to improve the anti-cancer effect [25].

In this study, a series of resveratrol derivatives modified with fluorinated groups, which improve biological activities, metabolic stability, and bioavailability [26,27], were comprehensively studied concerning their structure-activity relationship in anti-aging activity within separate inflammatory and oxidative stress cell models induced by lipopolysaccharide (LPS) and *tert*-butyl hydroperoxide (*t*-BHP). We then investigated whether the active derivative demonstrated a protective effect on D-galactose (D-gal)-stimulated aging mice and revealed the mechanism.

## 2. Results and Discussion

### 2.1. Resveratrol Derivatives

**Figure 1:**
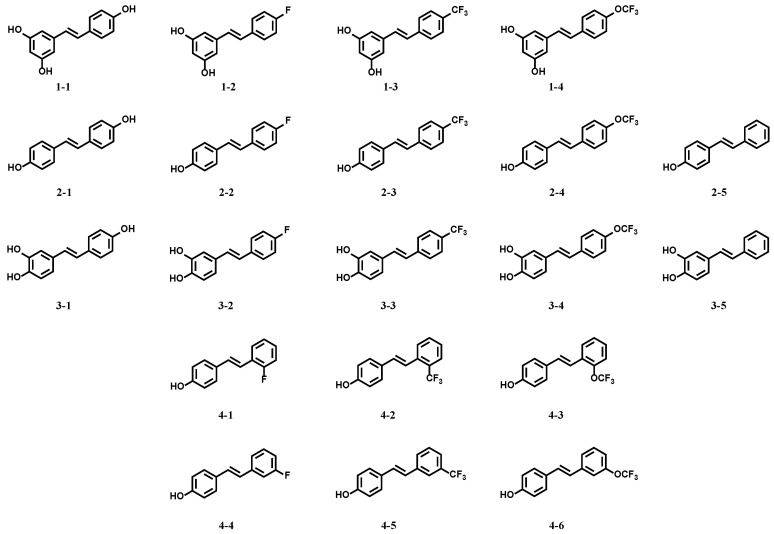
Structures of resveratrol and its derivatives.

Twenty resveratrol derivatives (see Figure 1 below), substituted with a fluorinated group (F, CF_3_, or CF_3_O) in different positions in the phenyl ring (*ortho*-, *meta*-, or *para*-), were designed and synthesized according to our previous publications [25,28].

### 2.2. In Vitro Study

#### 2.2.1. Initial Screening

Chronic inflammation is common in aging and age-related diseases. The relationship between aging and inflammation is accurately reflected in the term “inflamm-aging”, which is an important factor in the rate and lifespan of aging [29,30,31], representing a chronic, progressive increase in the inflammatory response associated with aging [32], Retarding “inflamm-aging” can improve the aging process and the health status of elderly people.

Oxidative stress also plays an important part in maintaining low-grade inflammation in aging and age-related diseases [28,33,34]. Aging, inflammation, and oxidative stress have causal relationships [35], which can cause and affect each other, and are complex. Therefore, we chose the inflammatory and oxidative stress cell models in Raw264.7 macrophage cells separately induced by LPS and *t*-BHP for initial the screening of the resveratrol derivatives.

##### Effects on Inflammation in LPS-Stimulated Raw264.7 Cells

Studying the anti-inflammatory effects of the resveratrol derivatives was carried out by determining the inhibitory effects on the LPS-stimulated excessive production of NO in Raw264.7 cells. As shown in Table 1, all of the hydroxyl-substituted resveratrol derivatives (**2–1**, **2–5**, **3–1,** and **3–5**) showed much lower IC_50_ values than resveratrol **1–1**, indicating they had higher anti-inflammatory activity. Among the fluorinated resveratrol derivatives, **4–6** showed the highest activity. For the 4-hydroxyl derivatives with the same fluorinated group, the *meta*-fluorinated derivative had the highest activity. (F, **4–4** > **4–1** or **1–2**; CF_3_, **4–5** > **4–2** or **1–3**; OCF_3_, **4–6** > **4–3** or **1–4**).

##### Effects on Oxidative Stress in *t*-BHP-Stimulated Raw264.7 Cells

**Figure 2:**
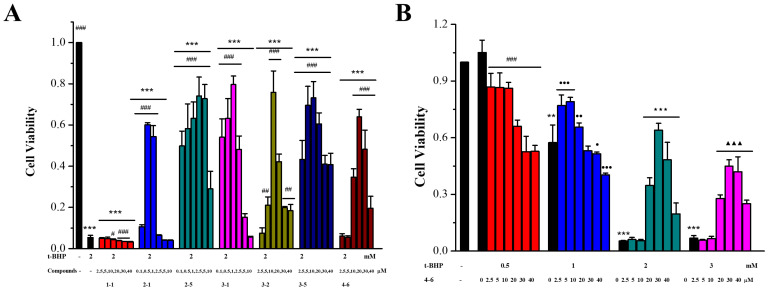
Effects of resveratrol derivatives on oxidative stress in Raw264.7 cells stimulated with *t*-BHP. (**A**) Cells were incubated with drugs for 24 h, then induced with 2 mM *t*-BHP for 3 h. (**B**) Cells were treated with **4–6** for 24 h, then induced with 0.5, 1, 2, or 3 mM *t*-BHP for 3 h. The MTT method was used to measure cytotoxicity. (**A**) *** *p* < 0.001 vs. the control group; ^#^ *p* < 0.05, ^##^ *p* < 0.01, ^###^ *p* < 0.001 vs. the 2 mM *t*-BHP group. (**B**) ** *p* < 0.01, *** *p* < 0.001 vs. the control group; ^###^ *p* < 0.001 vs. the 0.5 mM group; ^●^ *p* < 0.05, ^●●^ *p* < 0.01, ^●●●^ *p* < 0.001 vs. the 1 mM group; ^★★★^ *p* < 0.001 vs. the 2 mM group; ^▲▲▲^ *p* < 0.001 vs. the 3 mM group.

The ability of the resveratrol derivatives to inhibit oxidative cytotoxicity in Raw264.7 cells induced by *t*-BHP was determined using the MTT method in order to assess the resistance to oxidative stress. As illustrated in Figure 2, in contrast with the control group, the cell viability was decreased after inducement with different concentrations of *t*-BHP (Figure 2B). In the group induced with 2 mM *t*-BHP, six resveratrol derivatives (**2–1**, **2–5**, **3–1**, **3–2**, **3–5,** and **4–6**) obviously protected Raw264.7 cells against oxidative cytotoxicity induced by *t*-BHP (Figure 2A). However, there was no dose relationship. When high concentrations of derivatives were used, the cell viability decreased, which was mainly due to the toxic activity of the derivative itself (Figure S2). In addition, resveratrol **1–1** did not show any protective effect (Figure 2A), and the other derivatives (**1–2**, **1–3**, **1–4**, **2–2**, **2–3**, **2–4**, **3–3**, **3–4**, **4–1**, **4–2**, **4–3**, **4–4,** and **4–5**) showed low or no activity (Figure S1).

#### 2.2.2. Cellular Uptake

**Figure 3:**
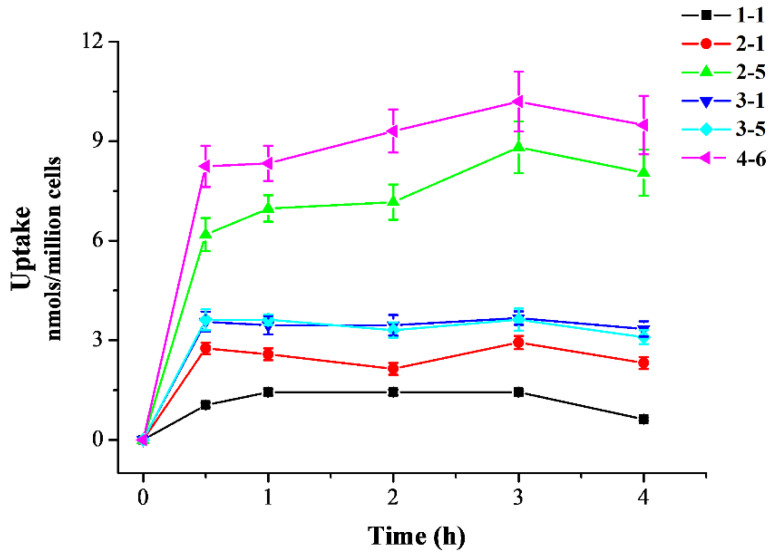
Cellular uptake of resveratrol and its active derivatives. Cells were pre-treated with compounds for 0.5, 1, 2, 3, or 4 h. Measurement of the intracellular compound concentrations was performed using HPLC.

After the initial screening of the resveratrol derivatives in the inflammation and oxidative stress cell models, active derivatives **2–1**, **2–5**, **3–1**, **3–5**, and **4–6** were chosen for further evaluation of cellular uptake activity. As shown in Figure 3, resveratrol **1–1** and only the polyhydroxyl-substituted resveratrol derivatives (**2–1**, **3–1,** and **3–5**) demonstrated low cell accumulation, only monohydroxyl-substituted derivative **2–5** demonstrated better cell accumulation, and *meta*-trifluoromethoxy-substituted derivative **4–6** demonstrated the best cell accumulation. The enhanced cellular uptake of **4–6** may have been attributed to the introduction of the *meta*-trifluoromethoxy group, potentially facilitating **4–6**’s ability to penetrate the cell membrane and enter the cell more effectively. Resveratrol, similar to other polyphenolic compounds, can be absorbed by small intestine mucosal cells and enter the circulatory system. In the body, they are easily degraded by metabolic enzymes in the liver and intestines, greatly reducing their bioavailability [36]. In this study, the resveratrol derivative **4–6**, which was substituted with the *meta*-trifluoromethoxy group, was selected as the chemical for further study. Filippis et al. also reported that a halogenated *E*-stibenol showed higher antioxidant activity than resveratrol in C2C12 cells, which was attributed to the presence of a trifluoromethyl group together with a chlorine atom [36].

#### 2.2.3. Effects of Resveratrol **1–1** and Its Active Derivative **4–6** on the Excessive Accumulation of ROS in *t*-BHP-Stimulated Raw264.7 Cells

**Figure 4:**
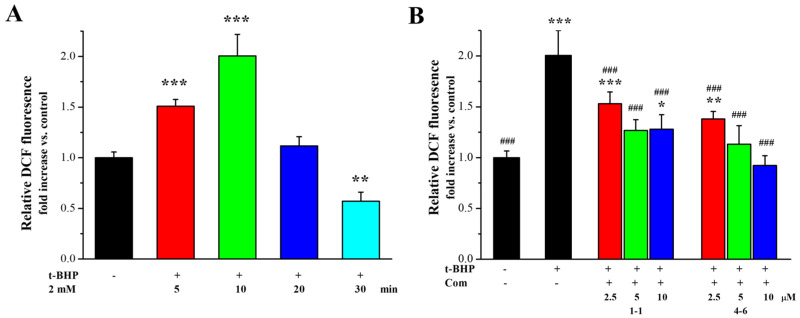
Effect of **1–1** or **4–6** on the excessive accumulation of ROS in Raw264.7 cells induced by *t*-BHP. (**A**) ROS levels induced by 2 mM *t*-BHP for different times. (**B**) ROS levels induced by 2 mM *t*-BHP for 10 min after cells were treated with 2.5, 5, and 10 μM of **1–1** or **4–6** for 1 h. Measurement of the ROS levels was carried out using flow cytometry. * *p* < 0.05, ** *p* < 0.01, *** *p* < 0.001 vs. the control group. ^###^ *p* < 0.001 vs. the *t*-BHP group.

ROS production is one of the most important indices underlying oxidative stress and plays an important part in the aging process. The effects of **1–1** and **4–6** on ROS production in Raw264.7 cells induced by *t*-BHP were further measured. As illustrated in Figure 4A, stimulation of the cells with *t*-BHP led to an increase in ROS, and inducement with *t*-BHP for 10 min increased the level of ROS. Pre-treatment with **1–1** or **4–6** caused the ROS accumulation induced by *t*-BHP to remarkably reduce in a dose-dependent manner, and the activity of **4–6** was slightly better than that of **1–1**. The prevention of ROS accumulation by **4–6** implied that **4–6** could protect cells against oxidative damage.

#### 2.2.4. Effects of Resveratrol **1–1** and Its Active Derivative **4–6** on Apoptosis in Raw264.7 Cells Induced by *t*-BHP

**Figure 5:**
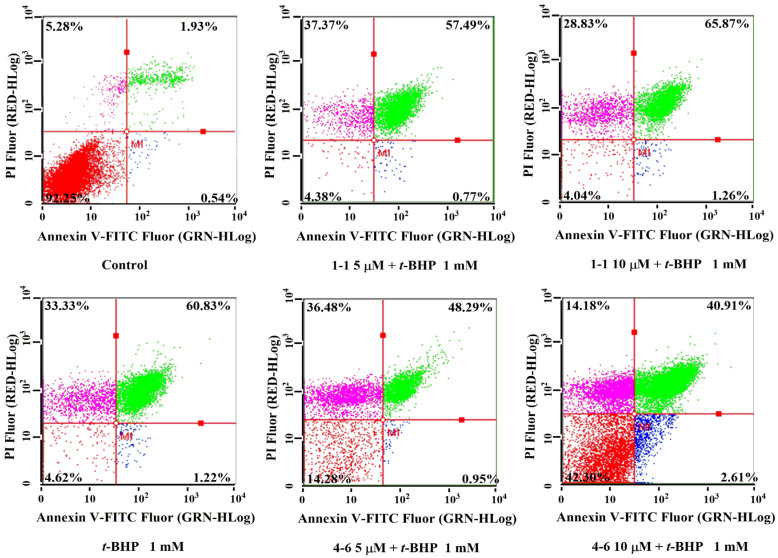
Flow cytometric analysis of apoptosis in Raw264.7 cells induced by *t*-BHP. Apoptosis induced by 1 mM *t*-BHP for 1 h after cells were pre-treated with 5 or 10 μM **1–1** or **4–6** for 1 h. Measurement of apoptosis was carried out using flow cytometry. The percentages of normal, early apoptosis, late apoptosis, and necrosis are indicated in each quadrant; sequentially lower-left, lower-right, upper-right, and upper-left.

There is a close relationship between oxidative stress and apoptosis [37,38]. *t*-BHP could induce cell apoptosis by activating oxidative stress. As seen in Figure 5, *t*-BHP caused cell apoptosis, evidenced by the 60.83% late apoptotic cells. Treatment with resveratrol **1–1** did not decrease the percentage of apoptotic cells, while **4–6** remarkably reduced the percentages of apoptosis. The result suggested that **4–6** could effectively inhibit cell apoptosis caused by oxidative stress.

#### 2.2.5. Molecular Docking Analysis of **1–1** and Its Derivative **4–6**

Resveratrol is the most well-known SIRT1 activator [39], which can simulate the anti-aging effect of heat restriction and participate in the regulation of the average life cycle of organic organisms [40,41,42,43]. Numerous studies have evaluated the computer-binding affinity of resveratrol or other active compounds to the human SIRT1 protein [44,45,46,47].

**Figure 6:**
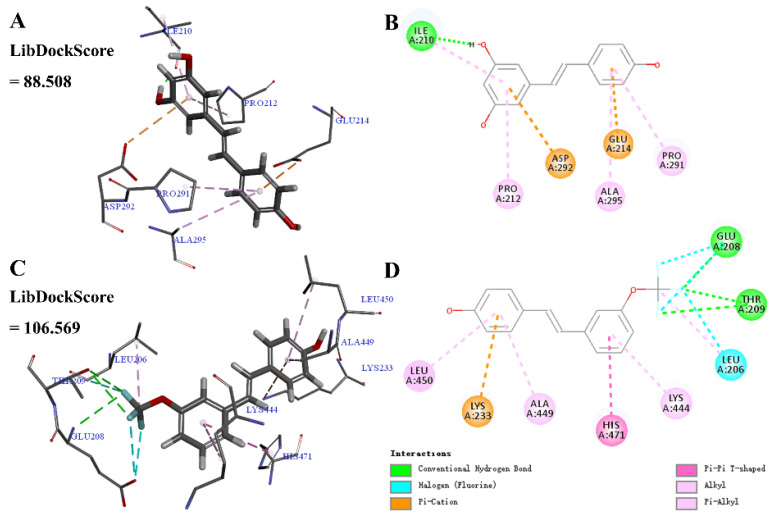
Molecular docking of resveratrol **1–1** and its derivative **4–6** with the SIRT1 protein structure (PDB ID 5BTR). (**A**,**B**) SIRT1-**1–1** interactions on 3D and 2D diagrams. (**C**,**D**) SIRT1-**4–6** interactions on 3D and 2D diagrams.

Molecular docking was calculated to predict the binding of the parent molecule resveratrol **1–1** or active derivative **4–6** with SIRT1. As shown in Figure 6, **1–1** may form conventional hydrogen bonds, pi-alkyl, and pi-cation interactions with SIRT1. **4–6** may form conventional hydrogen bonds, pi-alkyl, alkyl, halogen (fluorine), pi-cation, and pi-pi T-shaped interactions with SIRT1, among which, the *meta*-trifluoromethoxy group may form conventional hydrogen bonds, halogen (fluorine), and alkyl interactions. Moreover, the LibDockScore of **4–6** and SIRT1 was higher than that of **1–1** and SIRT1 (**4–6**, 106.569; **1–1**, 88.508), which was consistent with the comparison of inhibiting NO production (**4–6**, 11.1 ± 1.05 μM; **1–1**, 33.5 ± 3.03 μM). These results indicate that **4–6** displayed a significantly higher affinity towards SIRT1 than resveratrol **1–1**, which may be attributed to the enhanced biological activity of **4–6**.

### 2.3. In Vivo Study

The changes caused by the chronic injection of D-gal are similar to the natural aging of animals [48,49]. Therefore, D-gal-stimulated aging mice are used to study aging as a classic animal model, [50] in which brain aging is like that in humans, such as oxidative stress, inflammation, apoptosis, neuronal degradation, and so on [50,51]. The anti-aging activity of **4-6** was further assayed using aging mice caused by D-gal.

#### 2.3.1. Biochemical Analyses of Serum for Liver and Kidney Function Test

**Figure 7:**
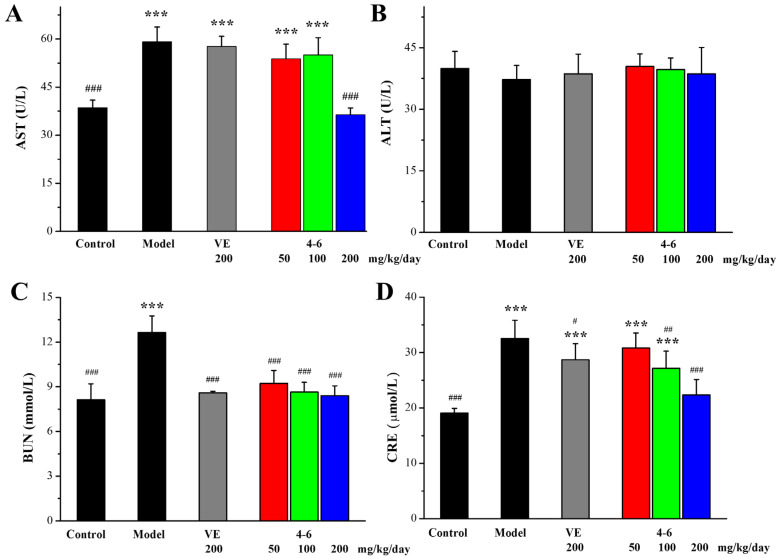
Serum AST (**A**), ALT (**B**), BUN (**C**), and CRE (**D**) levels for liver and kidney function. These biomarkers were assessed using the corresponding detection reagent kits. *** *p* < 0.001 vs. the control group. ^#^ *p* < 0.05, ^##^ *p* < 0.01, ^###^ *p* < 0.001 vs. the model group.

The extent of liver and kidney injury was evaluated by determining the levels of aspartate aminotransferase (AST), alanine aminotransferase (ALT), blood urea nitrogen (BUN), and creatinine (CRE) in the serum. Regarding liver damage, D-gal caused an increase in the AST level in the model group. Additionally, 200 mg/kg/day VE could not suppress the increase in AST, while 200 mg/kg/day **4–6** could suppress the D-gal-induced increase in AST, and **4–6** could reverse D-gal-induced liver damage. Regarding the levels of ALT, there were no significant differences among the control, model, VE, and **4–6** groups (Figure 7B, *p* > 0.05). Regarding kidney damage, D-gal caused increases in the levels of BUN and CRE. Both VE and **4–6** could reduce the level of BUN. 200 mg/kg/day VE could slightly reduce the level of CRE. Furthermore, **4–6** could reduce the level of CRE. These results indicate that 200 mg/kg/day **4–6** could reverse both liver and kidney injuries caused by D-gal.

#### 2.3.2. Effects of **4–6** on D-gal-Stimulated Oxidative Stress in the Serum 

**Figure 8:**
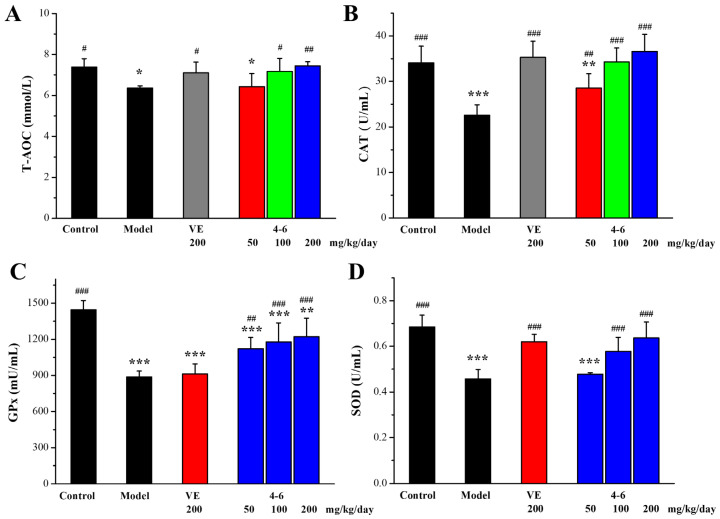
Effects of **4–6** on serum oxidative stress biomarkers in aging mice stimulated with D-gal. (**A**) T-AOC. (**B**) CAT. (**C**) GPx. (**D**) SOD. These oxidative stress biomarkers were assessed using their detection reagent kits. * *p* < 0.05, ** *p* < 0.01, *** *p* < 0.001 vs. the control group. ^#^ *p* < 0.05, ^##^ *p* < 0.01, ^###^ *p* < 0.001 vs. the model group.

Oxidative stress is an important aging mechanism induced by D-gal [51]. The total antioxidant capacity (T-AOC) and the activity of anti-oxidative defense enzymes catalase (CAT), superoxide dismutase (SOD), and glutathione peroxidase (GPx) in the serum of aging mice induced by D-gal as biomarkers of oxidative stress were measured. As illustrated in Figure 8, D-gal caused reductions in these biomarkers in the serum. 200 mg/kg/day VE could reverse the D-gal-induced reduction in T-AOC, CAT, and SOD, but could not increase the reduced level of GPx. For the intervention of **4–6**, it reversed the D-gal-induced reductions in serum T-AOC, CAT, GPx, and SOD in a concentration-dependent manner.

#### 2.3.3. Effects of **4-6** on Oxidative Stress Stimulated with D-gal in the Brain and Liver

Furthermore, the level of malondialdehyde (MDA), an end product of lipid peroxidation, and the activities of CAT, GPx, and SOD in the brain or liver of aging mice stimulated with D-gal as biomarkers of oxidative stress were measured.

**Figure 9:**
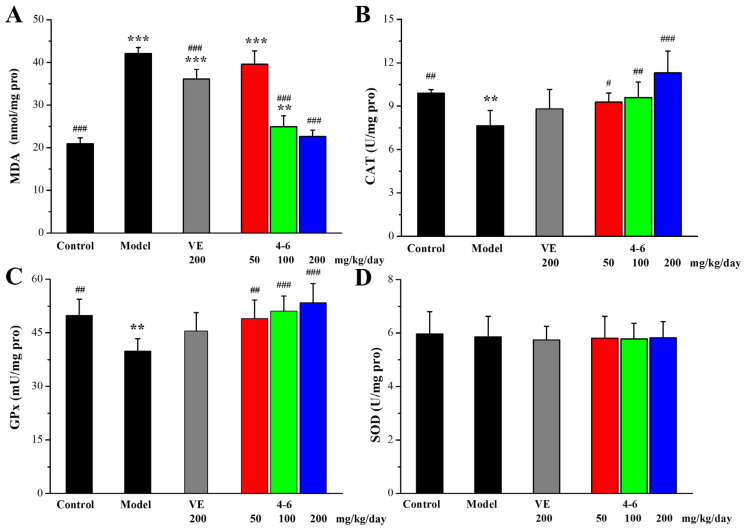
Effects of **4–6** on oxidative stress biomarkers in the brain. (**A**) MDA. (**B**) CAT. (**C**) GPx. (**D**) SOD. These oxidative stress biomarkers were assessed using their detection reagent kits. ** *p* < 0.01,*** *p* < 0.001 vs. the control group. ^#^ *p* < 0.05, ^##^ *p* < 0.01, ^###^ *p* < 0.001 vs. the model group.

As illustrated in Figure 9, in contrast with the control group, D-gal stimulated the increase in MDA level and the reductions in CAT and SOD activity in the brain. However, there were no significant differences in the GPx levels among the groups (Figure 9C, *p* > 0.05). Treatment with either VE or **4–6** could effectively reverse the changes in the MDA, CAT, and SOD levels stimulated with D-gal. The activity of **4–6** was better than that of VE.

**Figure 10:**
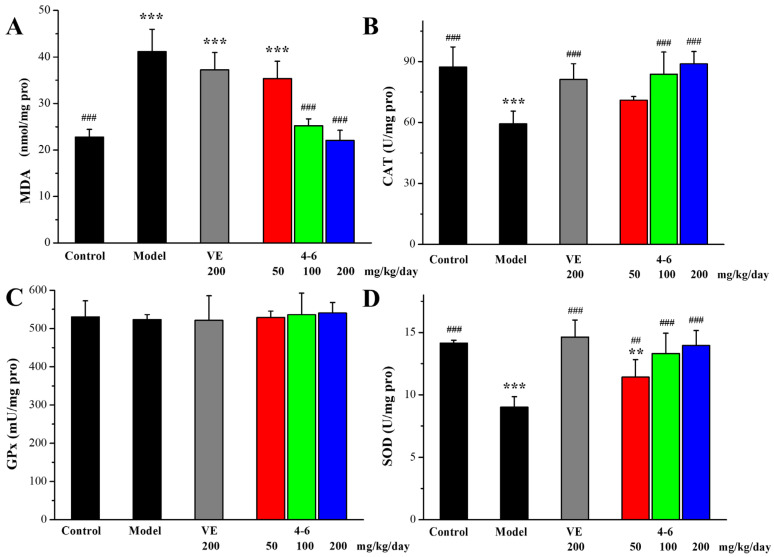
Effects of **4–6** on oxidative stress biomarkers in the liver. (**A**) MDA. (**B**) CAT. (**C**) GPx. (**D**) SOD. These oxidative stress biomarkers were assessed using their detection reagent kits. ** *p* < 0.01, *** *p* < 0.001 vs. the control group. ^##^ *p* < 0.01, ^###^ *p* < 0.001 vs. the model group.

As illustrated in Figure 10, in contrast with the control group, D-gal stimulated the increase in MDA levels and the reduction in CAT and GPx activity in the liver. However, there were no significant differences in the SOD levels among the groups (Figure 10D, *p* > 0.05). Treatment with either VE or **4–6** could effectively reverse these changes in MDA, CAT, and GPx levels. The activity of **4–6** was better than that of VE.

These results indicate that **4–6** could effectively increase the activity of anti-oxidative defense enzymes, further protect against oxidative stress, and reduce the production of lipid peroxidation.

#### 2.3.4. Effects of **4–6** on D-gal-Induced Inflammation in the Spleen Tissue

**Figure 11:**
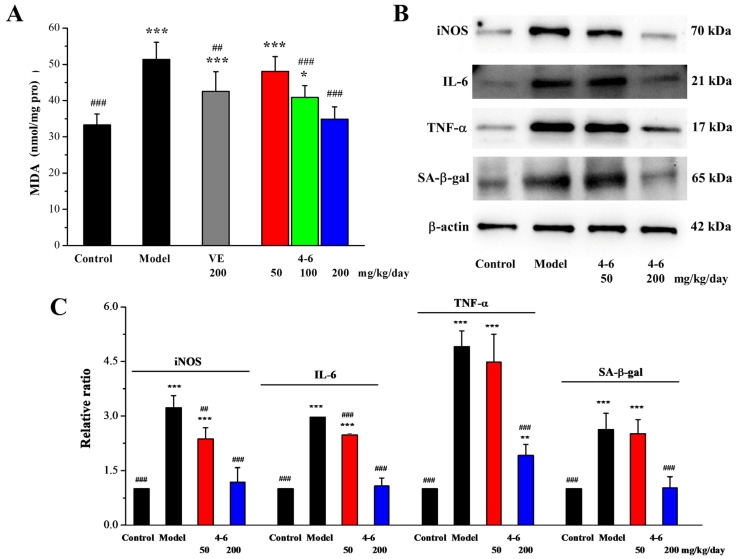
Effects of **4–6** on MDA (**A**), iNOS, IL-6, TNF-α, and SA-β-gal in the spleen of D-gal-stimulated aging mice (**B**). The MDA level was assessed using its detection reagent kit. The expressions of iNOS, IL-6, TNF-α, and SA-β-gal were analyzed using Western blotting. ImageJ 1.53e software was used to analyze the densitometric quantifications. * *p* < 0.05, ** *p* < 0.01, *** *p* < 0.001 vs. the control group. ^##^ *p* < 0.01, ^###^ *p* < 0.001 vs. the model group (**C**).

Oxidative stress can also be induced in the spleen of aging mice stimulated with D-gal. As illustrated in Figure 11A, in contrast with the control group, D-gal caused an increase in the MDA level of the spleen. Treatment with either VE or **4–6** could reverse the change in MDA level caused by D-gal.

In addition to oxidative stress, the spleen is a crucial organ in the human immune system, which has the function of synthesizing and secreting immune substances, and can also produce active substances related to immune regulation. As age increases, the structure and function of the spleen gradually decline. Therefore, we further evaluated whether **4–6** can protect the spleen in D-gal-induced aging mice.

Inducible nitric oxide synthase (iNOS) is a class of enzymes that utilizes the oxidative stress of NO to assist macrophages in fighting pathogens in the immune system. Cytokines are glycoproteins or small-molecule polypeptides, which are synthesized and secreted by tissue cells. They can regulate innate and adaptive immunity, participate in inflammatory reactions, repair damaged tissues, and so on. INOS and two cytokines (interleukin (IL)-6 and tumor necrosis factor-α (TNF-α)) were determined using Western blotting.

As illustrated in Figure 11B,C, in contrast with the control group, D-gal stimulated the increase in the iNOS, IL-6, and TNF-α expression levels of the spleen. Treatment with **4–6** decreased these increased expression levels. Meanwhile, the expression level of senescence-associated-β-galactosidase (SA-β-gal), a marker of aging, was also significantly increased in the spleen of aging mice stimulated with D-gal, while **4–6** could reverse the D-gal-induced change in SA-β-gal level in aging mice.

These results indicate that D-gal could cause oxidative stress and inflammation in the spleen of aging mice, while **4–6** could increase the body’s immunity by regulating iNOS and cytokines and protect the spleen against oxidative and inflammatory damage, thereby ameliorating the aging of mice.

#### 2.3.5. Effects on Histopathological Alternations in Aging Mice Caused by D-gal

**Figure 12:**
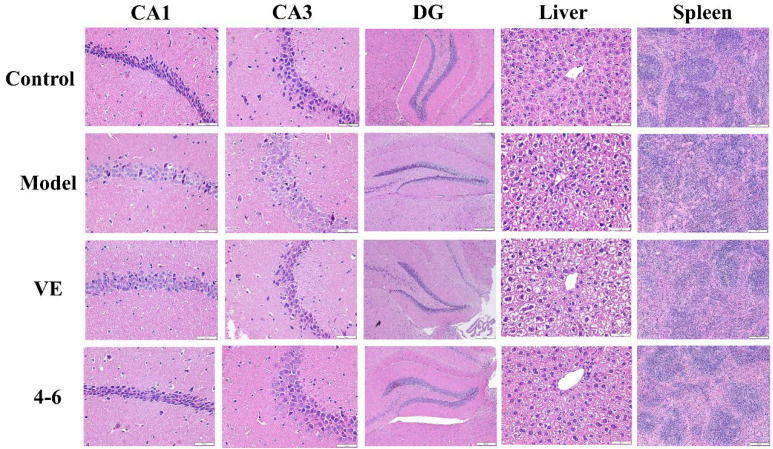
Effects on the histopathology of the hippocampus, liver, and spleen. HE staining was used for the paraffin sections. (Scale bar: hippocampus: CA1, 50 μM; CA3, 50 μM; DG, 200 μM. liver, 50 μM. spleen, 200 μM).

Histopathological studies on brain, liver, and spleen tissues were performed using HE staining. As illustrated in Figure 12, the morphology of the hippocampus, liver, and spleen of control mice was normal. In the model group, D-gal caused a loose structure, blurred membrane boundaries, and morphological changes in the DG area of the hippocampus as well as cell swelling, binuclear phenomenon in the liver tissue, and a reduction in the white pulp of the spleen tissue. These results suggest that hippocampal neurons and the liver and spleen were damaged by D-gal. However, **4–6** could reverse these D-gal-induced histopathological changes.

**Figure 13:**
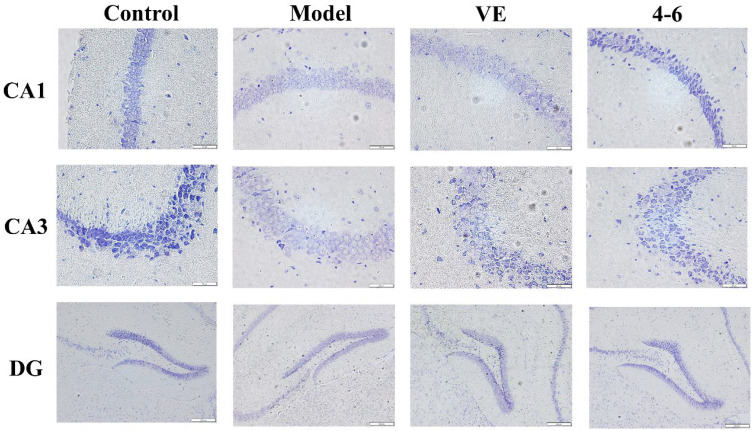
Effects on the histopathology of the subfields of the hippocampus (CA1, CA3, and DG). Nissl staining was used for the paraffin sections. (Scale bar: CA1, 50 μM; CA3, 50 μM; DG, 200 μM).

Nissl bodies are a feature of the structure of neurons and their quantity indicates the condition of the neuronal cell [52]. Furthermore, the brain tissue was stained via Nissl staining to observe the status of the neurons. The model group was slightly stained with Nissl, but VE and **4–6** could improve this situation, especially **4–6**, as illustrated in Figure 13.

#### 2.3.6. Effects on AchE and Ach in the Brain of Aging Mice Stimulated with D-gal

Acetylcholine (Ach) is an important neurotransmitter in the body and acetylcholin esterase (AchE) is a specific cholinergic marker protein, which can degrade acetylcholine in the synaptic cleft. The cholinergic system is important for learning and memory [53,54]. Clinically, the Ach level and AchE activity in the brain could be used to detect the biochemical indicators of aging. 

**Figure 14:**
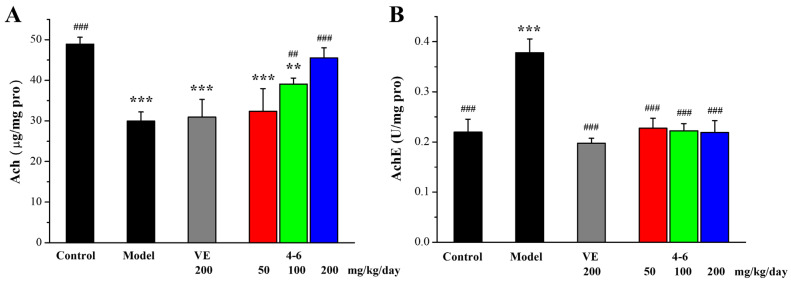
Effects of **4–6** on (**A**) Ach and (**B**) AchE in brain tissue. The Ach level and AchE activity were assessed using their corresponding detection reagent kits. ** *p* < 0.01, *** *p* < 0.001 vs. the control group. ^##^ *p* < 0.01, ^###^ *p* < 0.001 vs. the model group.

As illustrated in Figure 14, D-gal upregulated AchE expression, and, correspondently, decreased the level of Ach, in contrast with the control group. Treatment with **4–6** reversed the changes in Ach level and AchE activity. Treatment with **1–1** could reduce the activity of AchE but could not increase the level of Ach. These results suggest that **4–6** may improve cholinergic system dysfunction.

#### 2.3.7. Effects on Protein Expression Related to Aging, Oxidative Stress, and Apoptosis in the Brain Homogenate of Aging Mice

The brain aging of mice induced by D-gal is similar to that of humans. Furthermore, protein expression related to aging, oxidative stress, and apoptosis in the brain homogenate of aging mice was assayed via Western blotting.

**Figure 15:**
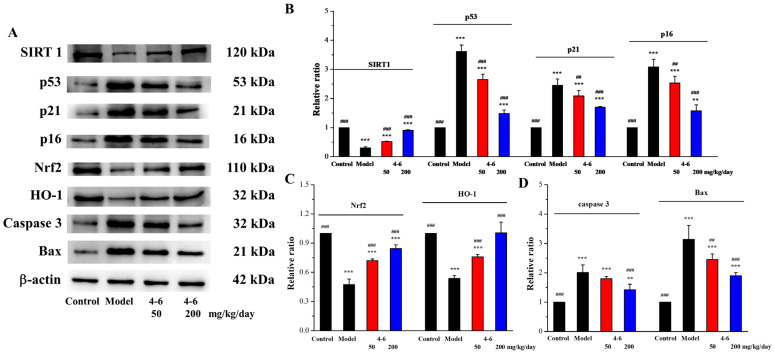
Effects on aging, oxidative stress, and apoptosis in brain tissue. (**A**) The expression levels of the corresponding proteins were analyzed with Western blotting. (**B**–**D**) Quantitative analysis of the corresponding proteins. ImageJ software was used to analyze the densitometric quantification. ** *p* < 0.01, *** *p* < 0.001 vs. the control group. ^##^ *p* < 0.01, ^###^ *p* < 0.001 vs. the model group.

SIRT1 is a protein related to mammalian aging, which is an important factor in regulating redox conditions, energy metabolism, cell apoptosis, and prolonging lifespan. SIRT1 can delay aging, extend lifespans, and prevent aging-related diseases [55], while SIRT1 deficiency can promote the expression of aging-related genes [56]. Moreover, p53/p21 (the p53-dependent pathway) and p16/Rb (the p53-independent pathway) are two critical signaling pathways for regulating cellular senescence [57,58]. Therefore, the expressions of SIRT1, p53, p21, and p16, senescence-associated proteins, were detected in the brain tissue. As illustrated in Figure 15, D-gal downregulated SIRT1 expression (Figure 15B) and upregulated the expression levels of p53, p21, and p16 (Figure 15B). Treatment with **4–6** increased the SIRT1 level and reduced the p53, p21, and p16 levels. These results suggest that **4–6** could intervene and reverse D-gal-induced mouse aging. Eren et al. reported that resveratrol also has corresponding effects on cells.

Nrf2 is a key signaling pathway for oxidative stress, binding to ARE to regulate phase II antioxidant enzymes like HO-1 and NQO1 [59]. Many studies have also shown that the Nrf2 pathway is a mechanism that counteracts the aging effect caused by D-gal. To confirm the protective effect of **4–6** via regulating the Nrf2 pathway, Nrf2 and HO-1 expression levels were assayed. As illustrated in Figure 15, D-gal downregulated Nrf2 and HO-1 expression (Figure 15A,C), while **4–6** effectively reversed these changes (Figure 15A,C). These results indicate that **4–6** could resist oxidative stress through the Nrf2 signaling pathway to protect the brain in aging mice.

The apoptosis of nerve cells can cause neural cell senescence, and this plays an important part in the aging of the nervous system [60,61,62]. D-galactose can activate both the extrinsic and intrinsic pathways of apoptosis [51]. Therefore, the effects of **4–6** on Bax and Caspase 3 expression in brain tissue were studied. As shown in Figure 15, D-gal upregulated the expression levels of Caspase 3 and Bax (Figure 15A,D), while **4–6** decreased these changes (Figure 15A,D). These results suggest that **4–6** could reverse D-gal-induced brain tissue apoptosis.

#### 2.3.8. Medicinal and Chemical Properties of 4–6

The medicinal and chemical properties of drug molecules can predict whether they can penetrate the blood-brain barrier. According to the literature, the standards for drug molecules to pass the blood-brain barrier are as follows: cLogP = 0–5, MW < 450, and PSA < 70 Å^2^ [63]. As shown in Table 2, the value of cLogP for **4–6** (4.96) was larger than that of the parent molecule **1–1** (2.99), which meant that **4–6** had stronger lipophilicity, the MW of **4–6** was less than 450, and the value of PSA for **4–6** (29.46) was smaller than that of the parent molecule **1–1** (60.68). These results suggest that **4–6** has the ability to penetrate the blood-brain barrier in vivo.

## 3. Conclusions

In summary, a series of resveratrol derivatives were investigated for their anti-inflammatory and anti-oxidative stress activities in cell models, and several derivatives with better activity were selected. The 4-Hydroxy-3′-trifluoromethoxy-substituted resveratrol derivative (**4–6**), demonstrating the best cell accumulation, could inhibit the excessive production of NO in an inflammatory cell model, inhibit oxidative cytotoxicity, and reduce ROS accumulation and the population of apoptotic cells in an oxidative stress cell model. In D-gal-stimulated aging mice, **4–6** could reverse liver and kidney injury (serum AST, ALT, BUN, and CRE), protect against oxidative stress damage (T-AOC, MDA, CAT, GPx, and SOD) in the serum, brain, and liver, and increase the body’s immunity (iNOS, IL-6, and TNF-α) in the spleen. In addition to protecting the brain from oxidative stress (biomarkers of oxidative stress), **4–6** could significantly improve the D-gal-induced histopathological alternations (HE and Nissl staining) in the hippocampus and the dysfunction of the cholinergic system (Ach and AchE). Furthermore, protein expression related to aging (SIRT1, p53, p21, and p16), oxidative stress (Nrf2 and HO-1), and apoptosis (Caspase 3 and Bax) in the brain measured using Western blotting also showed that **4–6** could ameliorate aging through protecting against oxidative stress and reducing apoptosis. In addition, the molecular docking simulation showed that **4–6** could effectively form interactions with SIRT1. Moreover, the predicted medicinal and chemical properties of **4–6** demonstrated the ability to penetrate the blood-brain barrier. 

This work revealed that *meta*-trifluoromethoxy-substituted-**4–6** deserved to be further investigated as an effective anti-aging candidate drug. However, further research is needed on the anti-aging effects and mechanisms of **4–6** on other tissues. In order to improve its bioavailability, nanomaterials loaded with **4–6** are also needed in further investigations. 

## 4. Materials and Methods

### 4.1. Synthesis

Derivatives were synthesized from a corresponding aldehyde and benzyl phosphonate via the Witting-Horner reaction, and their ^1^H and ^13^C NMR spectroscopy were witnessed in previously published papers [25,28] (See Supplementary Materials).

### 4.2. Cell Culture

Raw264.7 cells (Shanghai Institute of Biochemistry) were cultured in DMEM medium (Hyclone, Logan, UT, USA), including 1% antibiotic solution (Beyotime, Shanghai, China), 10% fetal bovine serum (Gibco, South America origin), and 5% CO_2_ at 37 °C.

#### 4.2.1. Determination of the Inhibition of NO

In 96-well plates, 100 μL of cells were seeded (1 × 10^6^ cells/mL). The cells were seeded with different compounds in fresh medium for 24 h after overnight incubation. A total of 75 μL of cell supernatant was mixed with 75 μL of fresh Griess reagent solution. The solution was determined at 540 nm using a microplate reader.

#### 4.2.2. t-BHP-Induced Oxidative Cytotoxicity

In 96-well plates, 100 μL of cells were seeded (2 × 10^5^ cells/mL). The cells were seeded with different compounds in fresh medium for 24 h after overnight incubation. Different concentrations of *t*-BHP solution were added and stimulated for 3 h. After washing with 10 mM PBS solution (pH 7.4), 100 μL of MTT (0.5 mg/mL) solution was added. 100 μL of DMSO was added after 4 h. The solution was determined at 570 nm using a microplate reader.

#### 4.2.3. Cell Cytotoxic Assay

In 96-well plates, 100 μL of cells were seeded (2 × 10^5^ cells/mL). The cells were seeded with different compounds in fresh medium for 24 h after overnight incubation. Then, 10 μL of MTT (5 mg/mL) solution was added. 100 μL DMSO was added after 4 h. The solution was measured at 570 nm using a microplate reader.

#### 4.2.4. Cellular Uptake

In 6-well plates, 2 mL of cells were seeded (1.5 × 10^6^ cells/mL). The cells were seeded with 50 μM of resveratrol (**1–1**) or its derivatives (**2–1**, **2–5**, **3–1**, **3–5,** and **4–6**) in fresh medium for 0.5, 1, 2, 3, or 4 h, after overnight incubation. The solution was washed twice with 1 mL of ice-cold PBS, then 0.8 mL of MeOH was added into each well at 4 °C for 24 h. After centrifugation, 200 μL of the supernatant was determined using a microplate reader.

λ_max_ and ε were measured in methanol (200 μL) using a 96-well plate:

**1–1**, A = −0.0086 + 0.00949C, λ = 308 nm; **2–1**, A = −0.0084 + 0.01519C, λ = 305 nm; **2–5**, A = −0.0110 + 0.01358C, λ = 305 nm; **3–1**, A = −0.0174 + 0.01213C, λ = 324 nm; **3–5**, A = 0.0084+ 0.01274C, λ = 326 nm; **4–6**, A = −0.0132+ 0.01507C, λ = 320 nm. (C, μM)

#### 4.2.5. Determination of Intracellular ROS

In 96-well plates, 100 μL of cells were seeded (2 × 10^5^ cells/mL). The cells were seeded with resveratrol **1–1** or derivative **4–6** in fresh medium for 1 h after overnight incubation. The cells were stimulated with 2 mM *t*-BHP for 10 min and washed with PBS solution. The cells were treated with 10 μM DCFH-DA for 30 min under dark conditions and then washed twice with PBS. The fluorescence intensity of the cells was determined using a benchtop flow cytometer (Millipore Guava easyCyte 8HT, California, MA, USA). (Ex/Em = 488 nm/530 nm).

#### 4.2.6. Determination of Cell Apoptosis

In 6-well plates, 2 mL of cells were seeded (1 × 10^6^ cells/mL). The cells were seeded with 5 or 10 μM resveratrol **1–1** or derivative **4–6** in fresh medium for 1 h after overnight incubation. The cells were stimulated with 1 mM *t*-BHP for 1 h and washed with PBS solution. According to the product manual, the cells were treated using a commercial reagent kit (Annexin V-FITC/PI Apoptosis Detection Kit, BD, Franklin Lakes, NJ, USA). A benchtop flow cytometer was used to analyze 10,000 cells.

### 4.3. Molecular Docking Studies of SIRT1 and the Compounds

The structure of SIRT1 (5BTR) was downloaded from the RCSB PDB database. The structure of SIRT1 was composed of the *N*-terminal region and the histone deacetylases (HDACs) domain, which was utilized for docking studies according to the literature [64]. The active site, AD5, of the SIRT1 (A chain) protein was used for molecular docking. The combined spherical area was x = −17.917889, y = 64.825133, z = 12.769794, radius = 14.5000. The LibDock module from Discovery studio 2020 was used for molecular docking, and the interaction with the highest score between SIRT1 and the compound (**1–1** or **4–6**) was analyzed.

### 4.4. Animal

C57bl/6j mice (7-month-old, male, weighing approximately 30 g, Pengyue, Jinan, China) could freely drink food and water at 23 ± 2 °C with a 12-h light/dark cycle. The mouse experiment was approved by the ethics committee of Liaocheng University (approval code: 2023022718).

#### 4.4.1. Treatment of Mice

Following a week of adaptation, the mice were randomly divided into six groups with eight mice in each group: the control group, the model group (the D-Gal-induced group), the VE-treated group (200 mg/kg/day), and the **4–6**-treated groups (50, 100, and 200 mg/kg/day). The mice in the control group were injected intraperitoneally and were administered saline orally. The mice in the model group were injected with D-Gal (500 mg/kg/day) daily for 10 weeks. In the VE or **4–6** groups, mice were injected with D-Gal (500 mg/kg/day) and were administered an oral saline suspension of VE or **4–6** daily for 10 weeks. After the last injection and oral administration dose, the mice were fasted for 12 h, then euthanized, and their tissues were immediately excised.

#### 4.4.2. Biochemical Analysis

The AST, ALT (Applygen Technologies Inc., Beijing, China), BUN, and CRE (Nanjing Jiancheng Bioengineering Institute) contents in the serum and the biomarkers of oxidative stress (T-AOC, CAT, SOD, GPx, and MDA) (Beyotime) in the brain, liver, spleen, and serum were assessed using commercial reagent kits according to the product manuals. The Ach level and AchE activity (Nanjing Jiancheng Bioengineering Institute, Nanjing, China) in the brain tissue were also determined using commercial reagent kits.

#### 4.4.3. Histopathological Examination

The brain, liver, and spleen were fixed with formalin, followed by embedding in paraffin and sectioning. HE was used to stain the sections, and the section of brain tissue was stained with Nissl. Then, photomicrographs were taken using an Olympus microscope (BX53 + DP80, Tokyo, Japan).

#### 4.4.4. Western Blot Assays

RIPA buffer containing protease inhibitors (Beyotime) was used to lyse tissues. The primary antibodies were as follows: IL-6 (Wanleibio, Shenyang, China,), TNF-α (Wanleibio), Caspase-3 (Cell Signaling Technology, Danvers, MA, USA), iNOS (Santa Cruz Biotechnology, Santa Cruz, CA, USA), Bax (Cell Signaling Technology), SIRT 1 (Wanleibio), p53 (Wanleibio), p21 (Wanleibio), SA-β-gal (Cell Signaling Technology), p16 (Wanleibio), Nrf2 (Beyotime), HO-1 (Wanleibio), and β-actin (Wanleibio). The secondary antibodies were as follows: HRP-labeled goat anti-mouse and anti-rabbit IgG(H + L) (Beyotime).

### 4.5. Data Analyses

Statistical analyses were carried out using a one-way analysis of variance and Dunnett’s multiple comparison test with GraphPad Prism software (version 5.01). The statistical significance was considered if *p* < 0.05.

## Figures and Tables

**Table 1:** The inhibition of the LPS-stimulated NO accumulation.

Num	IC_50_ (μM)	Num	IC_50_ (μM)	Num	IC_50_ (μM)
**1–2**	45.8 ± 2.26	**1–3**	82.5 ± 1.70	**1–4**	30.5 ± 1.55
**2–2**	16.5 ± 1.45	**2–3**	22.7 ± 0.99	**2–4**	23.6 ± 2.10
**3–2**	22.9 ± 1.38	**3–3**	21.9 ± 2.69	**3–4**	13.1 ± 0.20
**4–1**	23.4 ± 0.13	**4–2**	23.2 ± 1.76	**4–3**	26.0 ± 1.36
**4–4**	14.9 ± 1.12	**4–5**	13.8 ± 1.06	**4–6**	11.1 ± 1.05
**1–1**	33.5 ± 3.03	**2–1**	2.2 ± 0.01	**3–1**	3.02 ± 0.03
		**2–5**	18.8 ± 1.75	**3–5**	7.96 ± 0.23

**Table 2:** Medicinal and chemical properties of **4–6**.

Num	cLogP ^a^	Molecular Weight MW (g/mol)	Polar Surface Area PSA (Å^2^) ^a^
**4-6**	4.96	280.25	29.46
**1-1**	2.99	228.25	60.68

^a^ The data were calculated online from the website: www.molinspiration.com (accessed on 7 February 2023).

## Data Availability

Data are contained within the article and Supplementary Materials.

## Supplementary Materials

The following supporting information can be downloaded at: https://www.mdpi.com/article/10.3390/molecules29010086/s1, Figure S1: Effects of resveratrol derivatives on t-BHP-induced oxidative stress in Raw264.7 cells. Raw264.7 cells were treated with the compound for 24 h, and then induced with 2 mM t-BHP for 3 h. Measurement of cytotoxicity using the MTT assay. Data were expressed as means ± SD (n = 3) from independent experiments; Figure S2: Cell viability of resveratrol derivatives in Raw264.7 cells. Raw264.7 cells were treated with the compound for 24 h. Measurement of cytotoxicity using the MTT assay. Data were expressed as means ± SD (n = 3) from independent experiments; Figure S3: Synthetics routs of resveratrol derivatives. Refs. [25,28,65,66,67,68,69,70,71,72,73,74,75,76] are cited in supplementary file.
