# Supplementary material for: Discovery of a 4-Hydroxy-3′-Trifluoromethoxy-Substituted Resveratrol Derivative as an Anti-Aging Agent
Source: Molecules. 2023 Dec 22;29(1):86. doi: 10.3390/molecules29010086 (PMC10779923; doi:10.3390/molecules29010086)
Supplement: Supplementary file 1 [file molecules-29-00086-s001.zip › molecules-2696445-supplementary.pdf]

Discovery of a 4-Hydroxy-3′-Trifluoromethoxy-Substituted Resveratrol Derivative as an Anti-Aging Agent

[1] School of Pharmaceutical Sciences, Liaocheng University, 1 Hunan Street, Liaocheng 252059, China;

17852094217@163.com (Y.L.); 17753126694@163.com (X.C.); 15550579575@163.com (Z.T.); xuekunwang0610@126.com (X.W.);

[2] Liaocheng Key Laboratory of Quality Control and Pharmacodynamic Evaluation of Ganoderma Lucidum, Liaocheng University, 1 Hunan Street, Liaocheng 252059, China

Supporting Information

* Correspondence: yangjie1110@163.com (J.Y.); guoyunliu@126.com (G.L.);

Fax: (+86)-150-6350-5132 (G.L)

1. Effects of resveratrol derivatives on *t*-BHP-induced oxidative stress in Raw264.7 cells

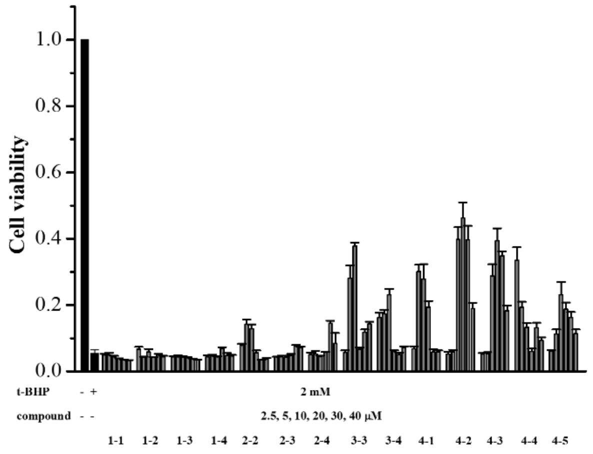

Figure S1. Effects of resveratrol derivatives on *t*-BHP-induced oxidative stress in Raw264.7 cells. Raw264.7 cells were treated with the compound for 24 h, and then induced with 2 mM *t*-BHP for 3 h. Measurement of cytotoxicity using the MTT assay. Data were expressed as means ± SD (n = 3) from independent experiments.

2. Cell viability of resveratrol derivatives in Raw264.7 cells

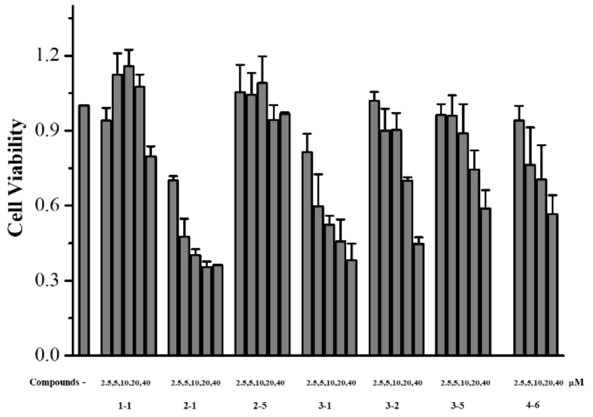

Figure S2. Cell viability of resveratrol derivatives in Raw264.7 cells. Raw264.7 cells were treated with the compound for 24 h. Measurement of cytotoxicity using the MTT assay. Data were expressed as means ± SD (n = 3) from independent experiments.

3. General procedure for the synthesis of resveratrol derivatives

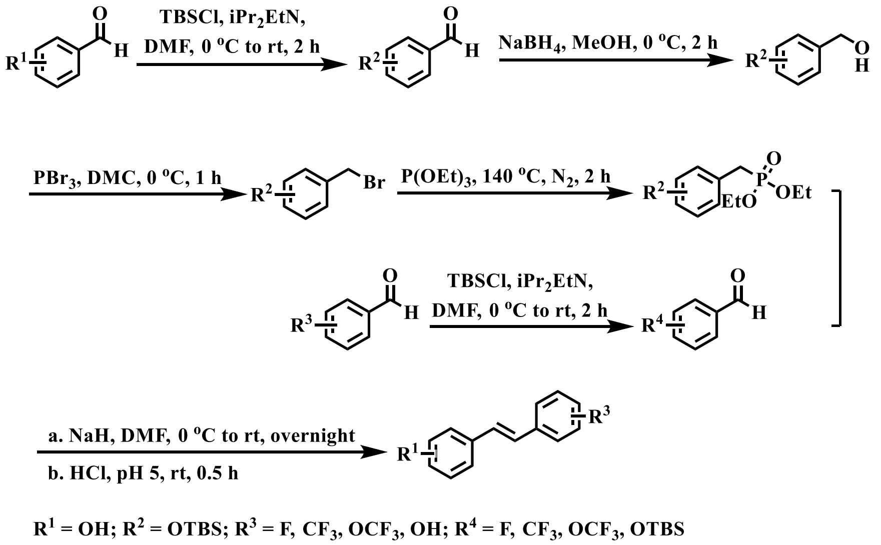

Figure S3. Synthetics routs of resveratrol derivatives.

All chemicals were obtained from Energy Chemical, Tan sole and sigma-aldrich.

Resveratrol derivatives were synthesized from corresponding aldehyde and benzyl phosphonate by the Witting-Horner reaction according to our previously published paper[1,2], and their ^{1}H and ^{13}C NMR spectroscopy witnessed previously published work.

Diethyl-(*tert*-butyldimethylsilyloxy)benzyl phosphonates were prepared according to the published procedure[3,4]. Briefly, NaH (10 mmol) was added in portions to a solution of diethyl- (*tert*-butyldimethylsilyloxy)benzyl phosphonate (2.6 mmol) in DMF at 0 °C. After 15 min stirring, fluorine-substituted aldehyde (2.0 mmol) in DMF (10 mL) was added dropwise and the reaction mixture was stirred overnight at room temperature under N_2 atmosphere. Then, the reaction was quenched by addition of H_2O at 0 °C and dilute HCl solution was added to the aqueous solution to adjust the pH to 5. The solution was extracted with ethyl acetate and the organic phase was washed with H_2O, dried with anhydrous Na_2SO_4, and concentrated in vacuo. The crude products were purification by column chromatography to afford the pure resveratrol derivatives.

2. ^{1}H NMR, ^{13}C NMR and ^{19}F NMR spectra

3,5-dihydroxy-4'-fluoro-*trans*-stilbene (**1-2**)[5]

^{1}H-NMR (400 MHz, DMSO-d_6), δ_H (ppm): 9.25(s, 2OH), 7.64-7.61 (m, 2H), 7.21 (t, J = 8.8 Hz, 2H), 7.07 (d, J = 16.4 Hz, 1H), 7.02 (d, J = 16.4 Hz, 1H), 6.44 (d, J = 2.0 Hz, 2H), 6.17(t, J = 2.0 Hz, 1H); ^{13}C-NMR(101 MHz, DMSO-d_6), δ_C(ppm): 163.18 (d, J_{C-F} = 245.43 Hz), 158.93, 139.07, 134.04 (d, J_{C-F} = 3.03 Hz), 129.27 (d, J_{C-F} = 2.02 Hz), 128.76 (d, J_{C-F} = 8.08 Hz), 127.05, 116.00 (d, J_{C-F} = 22.22 Hz), 105.06, 102.73; ^{19}F-NMR(DMSO-d_6), δ_F(ppm): -114.51.

3,5-dihydroxy-4’-trifluoromethyl-*trans*-stilbene (**1-3**)[6]

^{1}H-NMR (400 MHz, $CDCl_3$), δ_H (ppm): 7.62 (d, J = 8.8 Hz, 2H), 7.58 (d, J = 8.8 Hz, 2H), 7.05 (s, 2H), 6.60 (d, J = 2.0 Hz, 2H), 6.32(t, J = 2.0 Hz, 1H); ^{13}C-NMR(101 MHz, DMSO-d_6), δ_C(ppm): 158.53, 141.17 (d, J = 1.31 Hz), 138.12, 131.85, 126.91, 126.25, 125.42 (d, J = 3.54 Hz), 105.01, 102.90; ^{19}F-NMR(DMSO-d_6), δ_F(ppm): -60.84.

3,5-dihydroxy-4’-trifluoromethoxy-*trans*-stilbene (**1-4**)[7]

^{1}H-NMR (400 MHz, DMSO-d_6), δ_H (ppm): 9.29(s, 2OH), 7.71(d, J = 8.8 Hz, 2H), 7.35(d, J = 8.00 Hz, 2H), 7.09(s, 2H), 6.46(d, J = 2.0 Hz, 2H), 6.19(t, J = 2.0 Hz, 1H); ^{13}C-NMR(101 MHz, DMSO-d_6), δ_C(ppm): 158.97, 147.84 (d, J_{C-F} = 1.72 Hz), 138.84, 136.93, 130.67, 128.51, 126.69, 121.64, 119.23, 105.24, 103.00; ^{19}F-NMR(DMSO-d_6), δ_F(ppm): -56.83.

4,4’-dihydroxy*trans*-stilbene (**2-1**)[8]

^{1}H-NMR (400 MHz, DMSO-d$_6$), δ_H (ppm): 9.47(s, 2OH), 7.37(d, J = 8.0 Hz, 4H), 6.90(s, 2H), 6.77(d, J = 8.4 Hz, 4H); ^{13}C-NMR (101 MHz, DMSO-d$_6$), δ_C (ppm): 157.20, 129.03, 127.78, 125.64, 115.94.

4-hydroxy-4’-fluoro-*trans*-stilbene (**2-2**)[9]

^{1}H-NMR (400 MHz, DMSO-d_6), δ_H (ppm): 9.59(s, 1OH), 7.59(dd, J = 5.6 and 8.8 Hz, 2H), 7.42(d, J = 8.8 Hz, 2H), 7.19(t, J = 8.8 Hz, 2H), 7.11(d, J = 16.4 Hz, 1H), 7.03(d, J = 16.8 Hz, 1H), 6.79(d, J = 8.8 Hz, 2H); ^{13}C-NMR(101 MHz, DMSO-d_6), δ_C(ppm): 162.86(d, J = 244.82 Hz), 157.70, 134.55(d, J = 3.13 Hz), 128.77(d, J = 2.32 Hz), 128.40, 128.22, 128.14, 124.30, 115.96(d, J = 21.51 Hz), 115.75; ^{19}F-NMR(DMSO-d_6), δ_F(ppm): -115.29.

4-hydroxy-4’-trifluoromethyl-*trans*-stilbene (**2-3**) [8]

^{1}H-NMR (400 MHz, DMSO-d_6), δ_H (ppm): 9.66(s, 1OH), 7.74(d, J = 8.4 Hz, 2H), 7.67(d, J = 8.4 Hz, 2H), 7.47(d, J = 8.4 Hz, 2H), 7.32(d, J = 16.4 Hz, 1H), 7.11(d, J = 16.4 Hz, 1H), 6.79(d, J = 8.4 Hz, 2H); ^{13}C-NMR(101 MHz, DMSO-d_6), δ_C(ppm): 158.29, 142.14, 131.78, 128.77, 127.92, 126.85, 125.92(q, J = 3.84 Hz), 123.85, 116.02; ^{19}F-NMR(DMSO-d_6), δ_F(ppm): -60.76.

4-hydroxy-4’-trifluoromethoxy-*trans*-stilbene (**2-4**)[7]

^{1}H-NMR (400 MHz, DMSO-d_6), δ_H (ppm): 9.65(s, 1OH), 7.64(d, J = 8.8 Hz, 2H), 7.43(d, J = 8.4 Hz, 2H), 7.31(d, J = 8.0 Hz, 2H), 7.18(d, J = 16.4 Hz, 1H), 7.05(d, J = 16.4 Hz, 1H), 6.78(d, J = 8.4 Hz, 2H); ^{13}C-NMR(101 MHz, DMSO-d_6), δ_C(ppm):

158.02, 147.46(d, *J* = 1.72 Hz), 137.48, 130.18, 128.51, 128.19, 128.00, 123.89, 121.71, 119.28, 116.01; ^{19}F-NMR(DMSO-*d6*), δ_F(ppm): -56.89.

4-hydroxy-*trans*-stilbene (**2-5**) [8]

^{1}H-NMR (400 MHz, DMSO-d6), δ_H (ppm): 9.60(s, 1OH), 7.55(d, *J* = 7.2 Hz, 2H), 7.45(d, *J* = 8.8 Hz, 2H), 7.37(t, *J* = 7.6 Hz, 2H), 7.24(t, *J* = 7.2 Hz, 1H), 7.17(d, *J* = 16.8 Hz, 1H), 7.04(d, *J* = 16.4 Hz, 1H), 6.80(d, *J* = 8.4 Hz, 2H); ^{13}C-NMR (101 MHz, DMSO-d6), δ_C (ppm): 157.77, 137.97, 129.06, 128.88, 128.50, 128.32, 127.39, 126.46, 125.51, 116.00.

3,4,4'-trihydroxy-*trans*-stilbene (**3-1**)[10]

^{1}H-NMR (400 MHz, DMSO-d6), δ_H (ppm): 9.43(s, 1OH), 8.92(s, 2OH), 7.36(d, *J* = 8.4 Hz, 2H), 6.93(s, 1H), 6.81-6.79(m, 3H), 6.75-6.70(m, 3H); ^{13}C-NMR (101 MHz, DMSO-d6), δ_C (ppm): 157.15, 145.80, 145.44, 129.60, 129.02, 127.77, 126.00, 125.51, 118.45, 116.14, 115.90, 113.39.

3,4-dihydroxy-4'-fluoro-*trans*-stilbene (**3-2**)[11]

^{1}H-NMR (400 MHz, DMSO-*d6*), δ_H (ppm): 9.048(s, 2OH), 7.59(dd, *J* = 5.6 and 8.4 Hz, 2H), 7.18(t, *J* = 8.8 Hz, 2H), 7.04-6.99(m, 2H), 6.93(d, *J* = 16.4 Hz, 1H), 6.87(dd, *J* = 1.6 and 8.4 Hz, 1H), 6.74(d, *J* = 8.0 Hz, 1H); ^{13}C-NMR(101 MHz, DMSO-*d6*), δ_C(ppm): 162.82(d, *J* = 244.72 Hz), 145.99, 145.80, 134.54(d, *J* = 3.13 Hz), 129.14(d, *J* = 2.32 Hz), 128.95, 128.22(d, *J* = 7.88 Hz), 124.17, 118.92, 116.08, 115.92(d, *J* = 21.41 Hz), 113.71; ^{19}F-NMR(DMSO-*d6*), δ_F(ppm): -110.62.

3,4-dihydroxy-4'-trifluoromethyl-*trans*-stilbene (**3-3**)[11]

^{1}H-NMR (400 MHz, DMSO-*d6*), δ_H (ppm): 9.21(s, 1OH), 8.99(s, 1OH), 7.76(d, *J* = 8.0 Hz, 2H), 7.68(d, *J* = 8.4 Hz, 2H), 7.26(d, *J* = 16.4 Hz, 1H), 7.04(d, *J* = 2.0 Hz, 1H), 7.03(d, *J* = 16.4 Hz, 1H), 6.94(dd, *J* = 2.0 and 8.0 Hz, 1H), 6.76(d, *J* = 8.4 Hz, 1H); ^{13}C-NMR(101 MHz, DMSO-*d6*), δ_C(ppm): 146.20, 145.43, 141.72(d, *J* = 1.21 Hz), 131.75, 128.04, 126.42, 125.40(d, *J* = 3.64 Hz), 123.29, 119.12, 115.68, 113.65; ^{19}F-NMR(DMSO-*d6*), δ_F(ppm): -60.76.

3,4-dihydroxy-4'-trifluoromethoxy-*trans*-stilbene (**3-4**) [7]

^{1}H-NMR (400 MHz, DMSO-*d6*), δ_H (ppm): 9.18(s, 1OH), 8.98(s, 1OH), 7.66(d, *J* = 8.8Hz, 2H), 7.33(d, *J* = 8.4 Hz, 2H), 7.12(d, *J* = 16.4 Hz, 1H), 7.01(d, *J* = 2.0 Hz, 1H), 6.97(d, *J* = 16.4 Hz, 1H), 6.89(dd, *J* = 2.0 and 8.0 Hz, 1H), 6.75(d, *J* = 8.0 Hz, 1H); ^{13}C-NMR(101 MHz, DMSO-*d6*), δ_C(ppm): 147.38(d, *J* = 1.62 Hz), 146.27, 145.82,

137.44, 130.54, 128.71, 127.97, 123.74, 121.60, 119.19, 116.09, 113.87; ^{19}F-NMR(DMSO-d_6), δ_F(ppm): -56.84.

3,4-dihydroxy-*trans*-stilbene (**3-5**)[8]

^{1}H-NMR (400 MHz, DMSO-d$_6$), δ_H (ppm): 9.06(s, 2OH), 7.54(d, J = 7.6 Hz, 2H), 7.35(t, J = 7.6 Hz, 2H), 7.23(t, J = 7.2 Hz, 1H), 7.09(d, J = 16.4 Hz, 1H), 7.01(d, J = 2.4 Hz, 1H), 6.94(d, J = 16.4 Hz, 1H), 6.89(dd, J = 2.0 and 8.1 Hz, 1H), 6.75(d, J = 8.4 Hz, 1H); ^{13}C-NMR (101 MHz, DMSO-d$_6$), δ_C (ppm): 146.01, 145.81, 137.90, 129.19, 128.99, 127.29, 126.41, 125.33, 118.99, 116.10, 113.73.

4-hydroxy-2'-fluoro-*trans*-stilbene (**4-1**)[9]

^{1}H-NMR (400 MHz, DMSO-d$_6$), δ_H (ppm): 9.66(s, 1OH), 7.75(t, J = 8.0 Hz, 1H), 7.46(d, J = 8.8 Hz, 2H), 7.28-7.17(m, 4H), 7.08(d, J = 16.8 Hz, 1H), 6.80(d, J = 8.8 Hz, 2H); ^{13}C-NMR (101 MHz, DMSO-d$_6$), δ_C (ppm): 161.07(d, J = 247.85 Hz), 158.08, 131.59(d, J = 4.55 Hz), 128.93(d, J = 8.38 Hz), 128.50, 128.19, 127.36(d, J = 3.64 Hz),125.51(d, J = 11.82 Hz), 125.01(d, J = 3.33 Hz), 117.07(d, J = 3.54 Hz), 116.15(d, J = 22.02 Hz), 115.99; ^{19}F-NMR (400 MHz, DMSO-d$_6$), δ_F (ppm): -118.80.

4-hydroxy-2'-trifluoromethyl-*trans*-stilbene (**4-2**)[1]

^{1}H-NMR (400 MHz, CDCl$_3$), δ_H (ppm): 7.77(d, J = 8.0 Hz, 1H), 7.67(d, J = 7.6 Hz, 1H), 7.54(t, J = 7.2 Hz, 1H), 7.45(d, J = 8.8 Hz, 2H), 7.36-7.31(m, 2H), 7.05(d, J = 16 Hz, 1H), 6.87(d, J = 8.4 Hz, 2H); ^{13}C-NMR(101 MHz, CDCl$_3$), δ_C(ppm): 155.66, 136.65, 132.00, 131.81, 129.96, 128.40, 127.41(d, J = 29.80 Hz), 126.82, 126.72, 125.97(q, J = 5.76 Hz), 123.12, 122.31(d, J = 1.82 Hz), 115.675; ^{19}F-NMR(CDCl$_3$), δ_F(ppm): -59.39.

4-hydroxy-2'-trifluoromethoxy-*trans*-stilbene (**4-3**)[1]

^{1}H-NMR (400 MHz, DMSO-d_6), δ_H (ppm): 9.72(s, 1OH), 7.94(d, J = 8.0 Hz, 1H), 7.70(d, J = 8.0 Hz, 1H), 7.66(t, J = 7.6 Hz, 1H), 7.43-7.40(m, 3H), 7.25(d, J = 16 Hz, 1H), 7.15(dd, J = 16.0 and 2.0 Hz, 1H), 6.81(d, J = 8.4 Hz, 2H); ^{13}C-NMR(101 MHz, DMSO-d_6), δ_C(ppm): 158.49, 136.59, 133.57, 133.09, 128.76, 127.88, 127.59, 127.27, 126.24(q, J = 5.86 Hz), 125.99(d, J = 26.36 Hz), 123.65, 119.98(d, J = 1.72 Hz), 116.19; ^{19}F-NMR(DMSO-d_6), δ_F(ppm): -58.09.

4-hydroxy-3'-fluoro-*trans*-stilbene (**4-4**)[12]

^{1}H-NMR (400 MHz, DMSO-d$_6$), δ_H (ppm): 9.65(s, 1OH), 7.44(d, J = 8.4 Hz, 2H), 7.41-7.35(m, 3H), 7.25(d, J = 16.4 Hz, 1H), 7.05-7.00(m, 2H), 6.80(d, J = 8.4 Hz, 2H); ^{13}C-NMR (101 MHz, DMSO-d$_6$), δ_C (ppm): 164.23(d, J = 243.41 Hz), 158.00, 140.76(d, J

= 8.08 Hz), 130.86(d, *J* = 8.08 Hz), 130.43, 128.49, 128.08, 124.23(d, *J* = 3.03), 122.79(d, *J* = 2.02 Hz), 115.97, 113.98(d, *J* = 21.21 Hz), 112.50(d, *J* = 22.22 Hz); ^{19}F-NMR (400 MHz, DMSO-d_6), δ_F (ppm): -113.53.

4-hydroxy-3'-trifluoromethyl-*trans*-stilbene (**4-5**)[13]

^{1}H-NMR (400 MHz, $CDCl_3$), δ_H (ppm): 7.71(s, 1H), 7.63(d, *J* = 7.2 Hz, 1H), 7.48-7.41(m, 4H), 7.12(d, *J* = 16.4 Hz, 1H), 6.98(d, *J* = 16.0 Hz, 1H), 6.85(d, *J* = 8.8 Hz, 2H); ^{13}C-NMR(101 MHz, $CDCl_3$), δ_C(ppm): 155.57, 138.37, 131.52(q, *J* = 32.02), 129.91, 129.77, 129.29, 129.04, 128.17, 125.52, 125.12, 123.70(q, *J* = 3.74), 122.85(q, *J* =3.74), 115.71; ^{19}F-NMR($CDCl_3$), δ_F(ppm): -62.78.

4-hydroxy-3'-trifluoromethoxy-*trans*-stilbene (**4-6**)[14]

^{1}H-NMR (400 MHz, $CDCl_3$), δ_H (ppm): 7.42-7.37(m, 3H), 7.35-7.32(m, 2H), 7.08-7.04(m, 2H), 6.95(d, *J* = 16.0 Hz, 1H), 6.85(d, *J* = 8.8 Hz, 2H); ^{13}C-NMR(101 MHz, $CDCl_3$), δ_C(ppm): 155.66, 149.68, 139.80, 129.86(d, *J* = 2.22 Hz), 129.68, 128.15, 125.08, 124.61, 121.78, 119.34, 118.38, 115.70; ^{19}F-NMR($CDCl_3$), δ_F(ppm): 57.68.

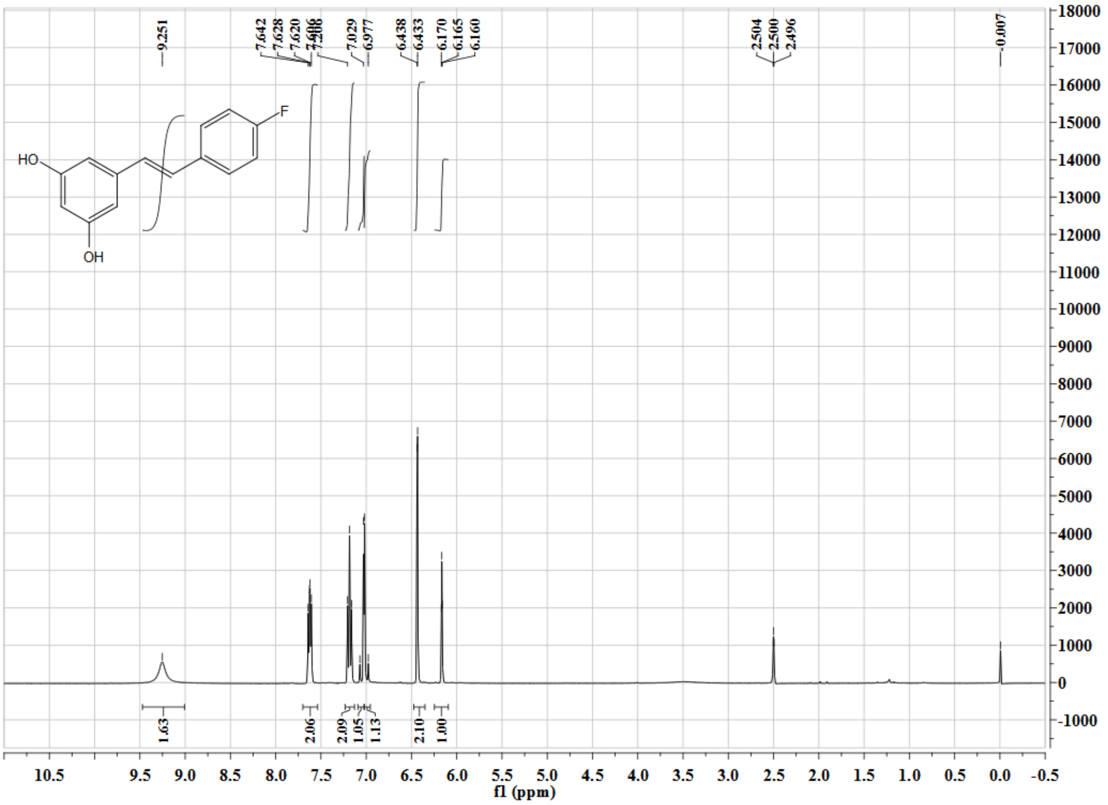

(up) ^{1}H NMR spectrum of compound **1-2** (400 MHz, DMSO-d_6)

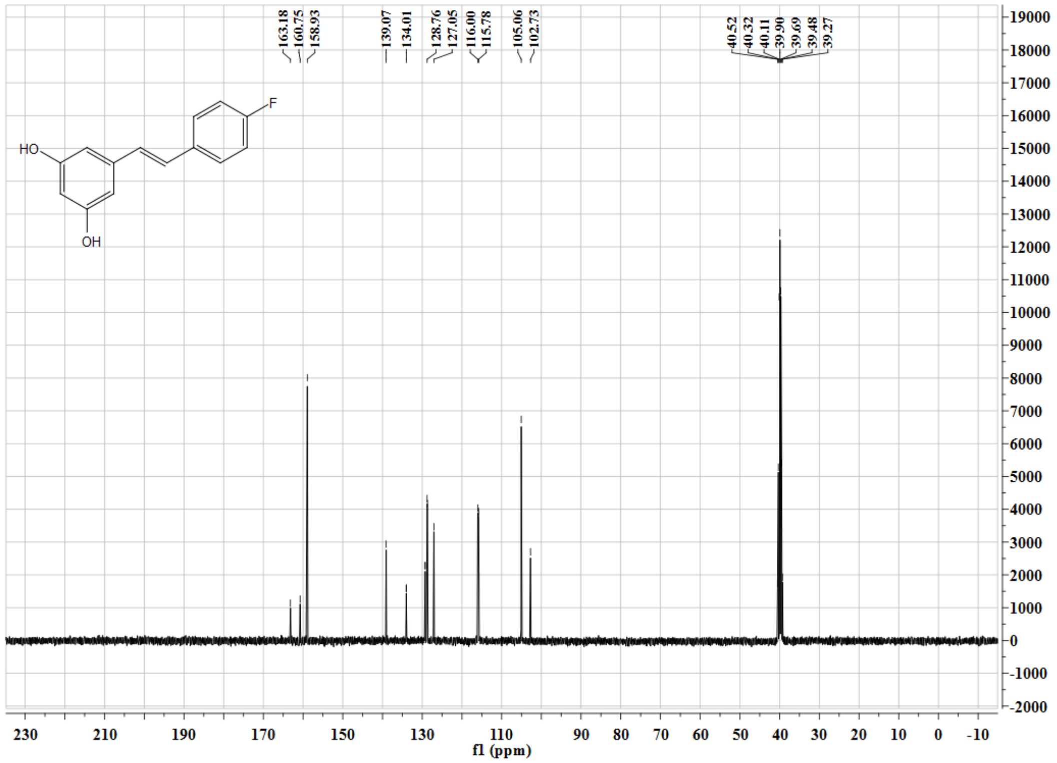

(up) ^{13}C NMR spectrum of compound **1-2** (101 MHz, DMSO-d_6)

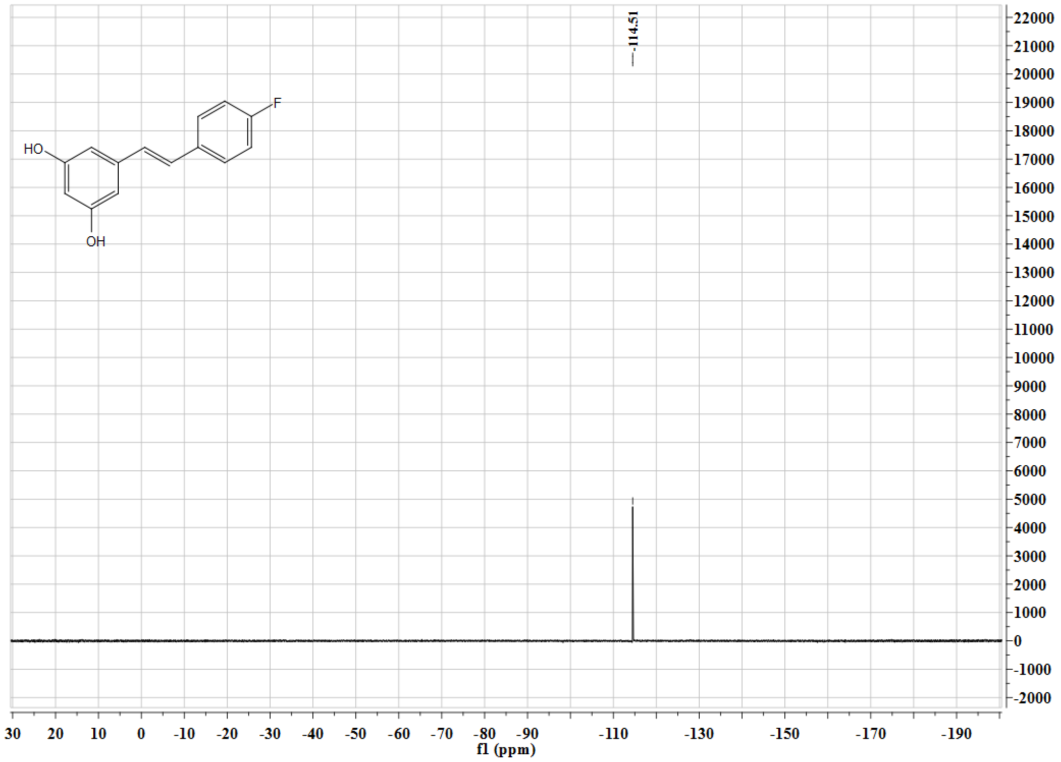

(up) ^{19}F NMR spectrum of compound **1-2** (400 MHz, DMSO-d_6)

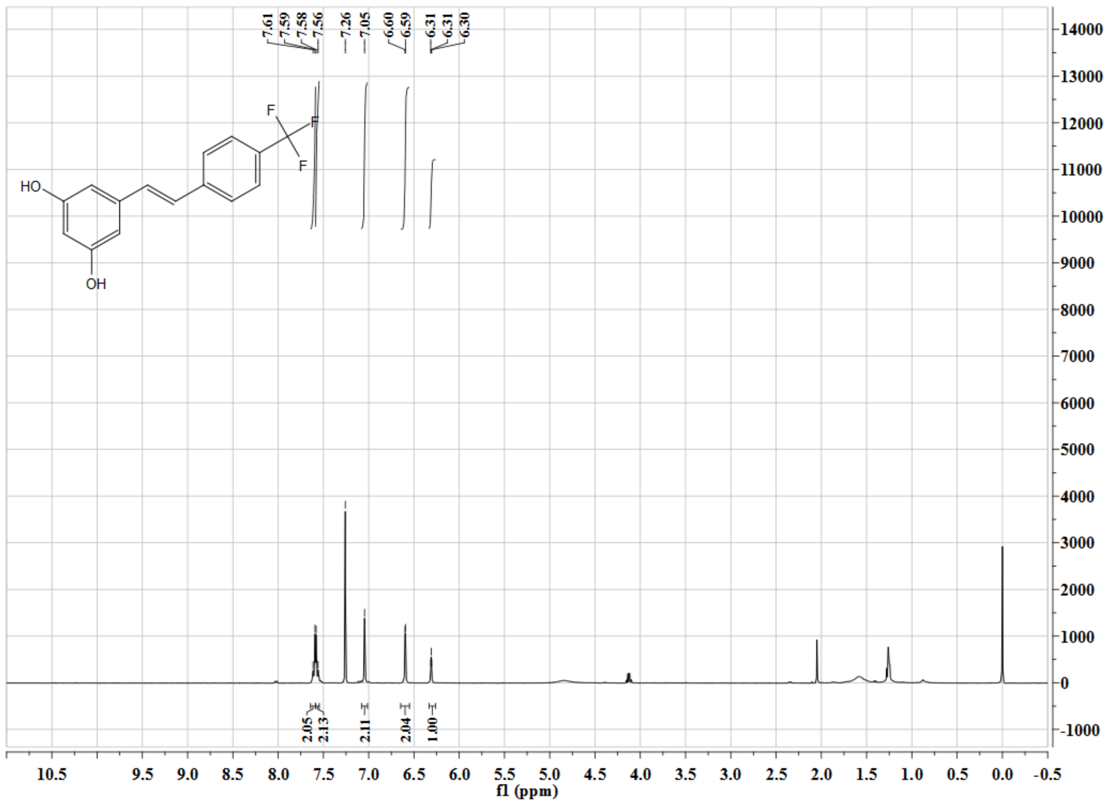

(up) ^{1}H NMR spectrum of compound **1-3** (400 MHz, $CDCl_3$)

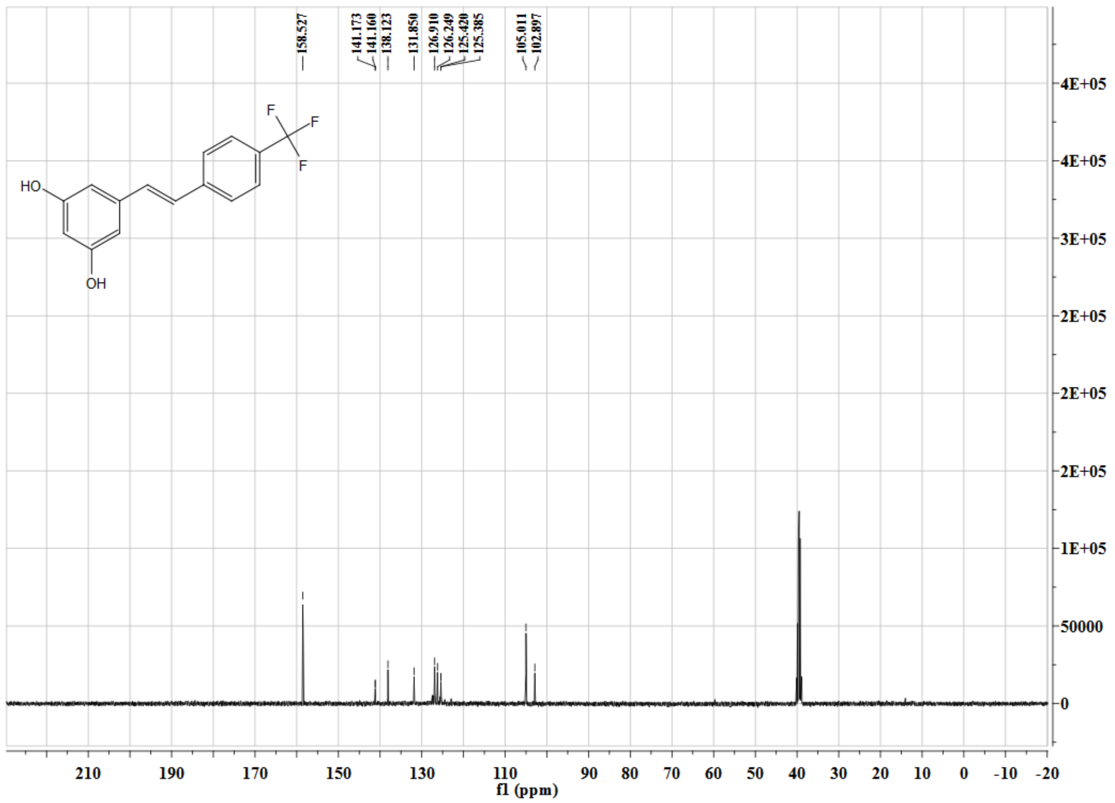

(up) ^{13}C NMR spectrum of compound **1-3** (101 MHz, DMSO-d_6)

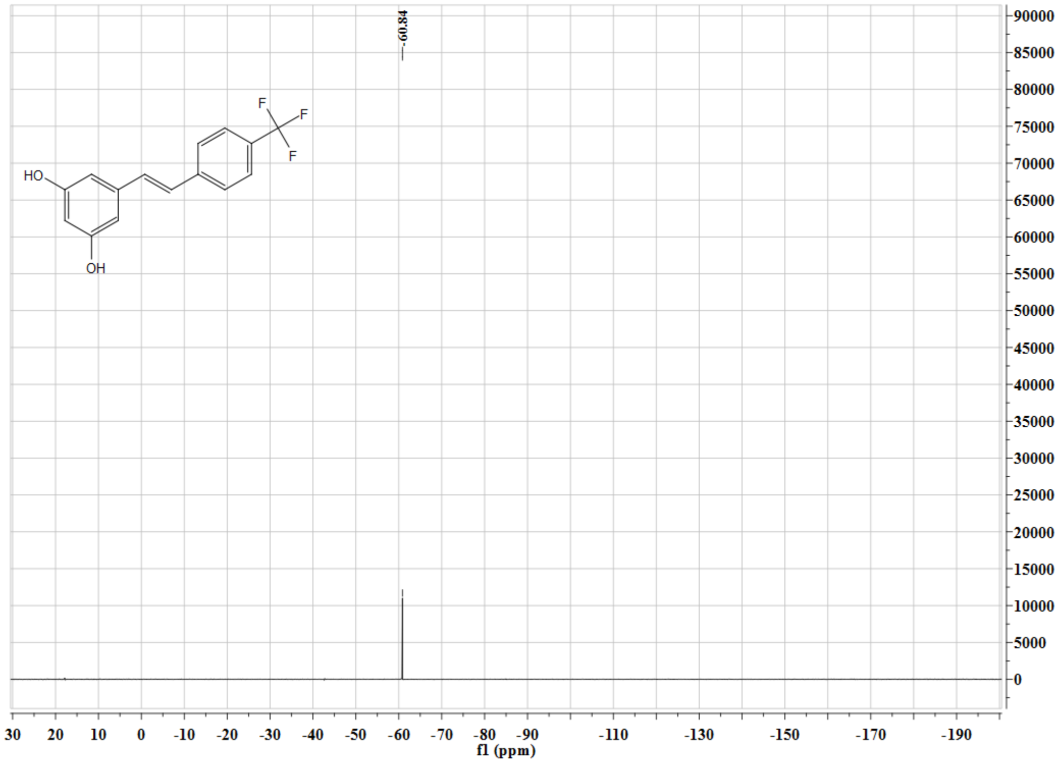

(up) ^{19}F NMR spectrum of compound **1-3** (400 MHz, DMSO-d_6)

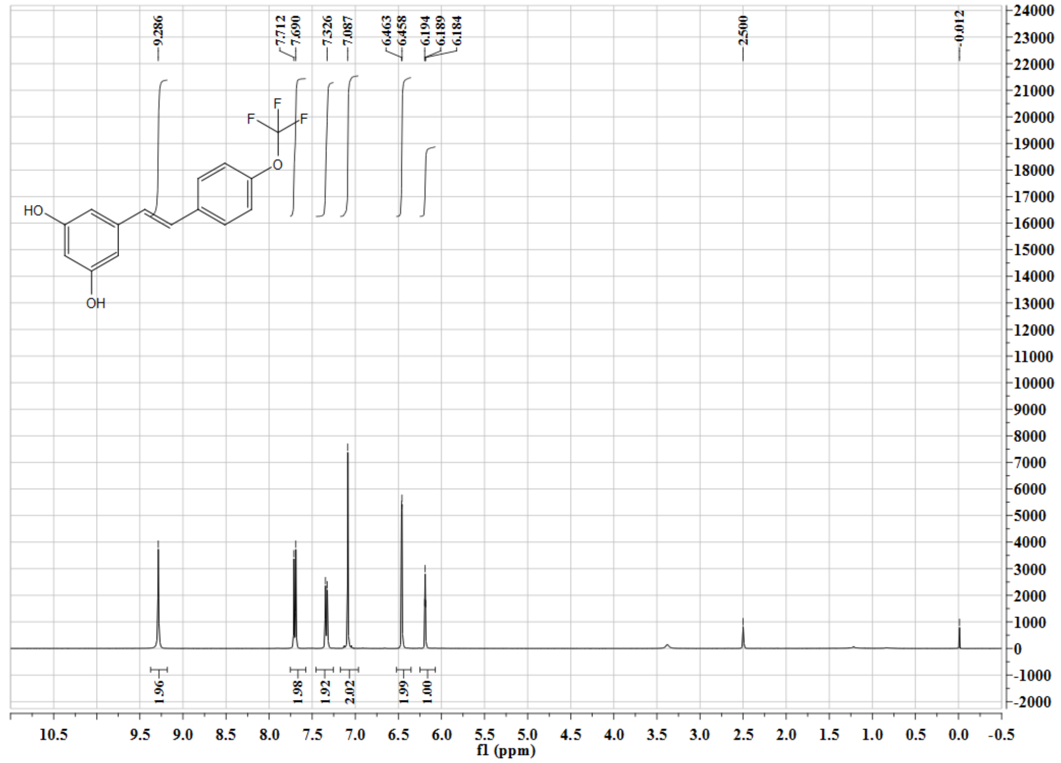

(up) ^{1}H NMR spectrum of compound **1-4** (400 MHz, DMSO-d_6)

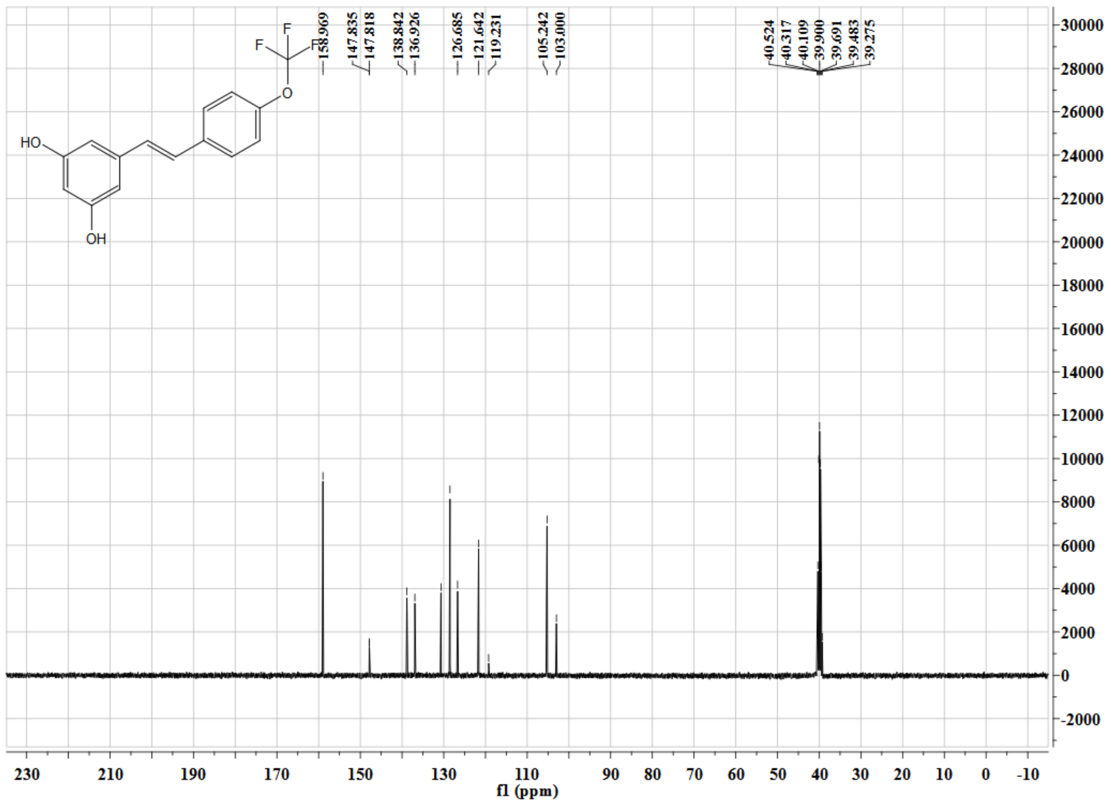

(up) ^{13}C NMR spectrum of compound **1-4** (101 MHz, DMSO-d_6)

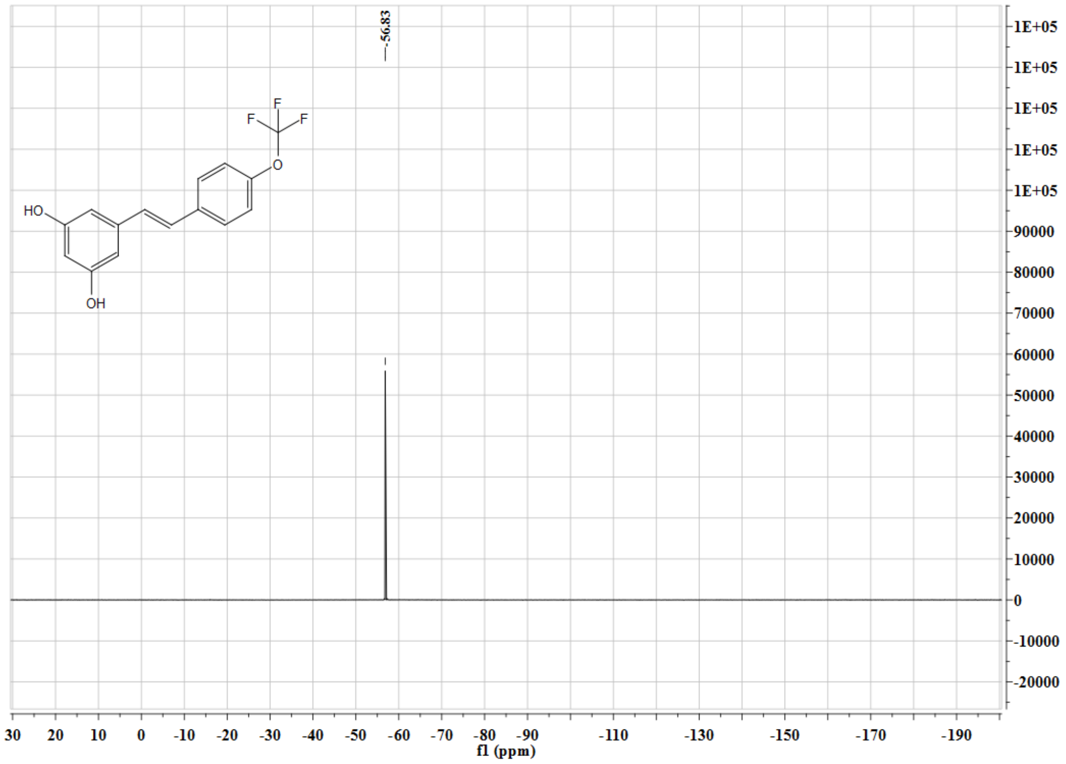

(up) ^{19}F NMR spectrum of compound **1-4** (400 MHz, DMSO-d_6)

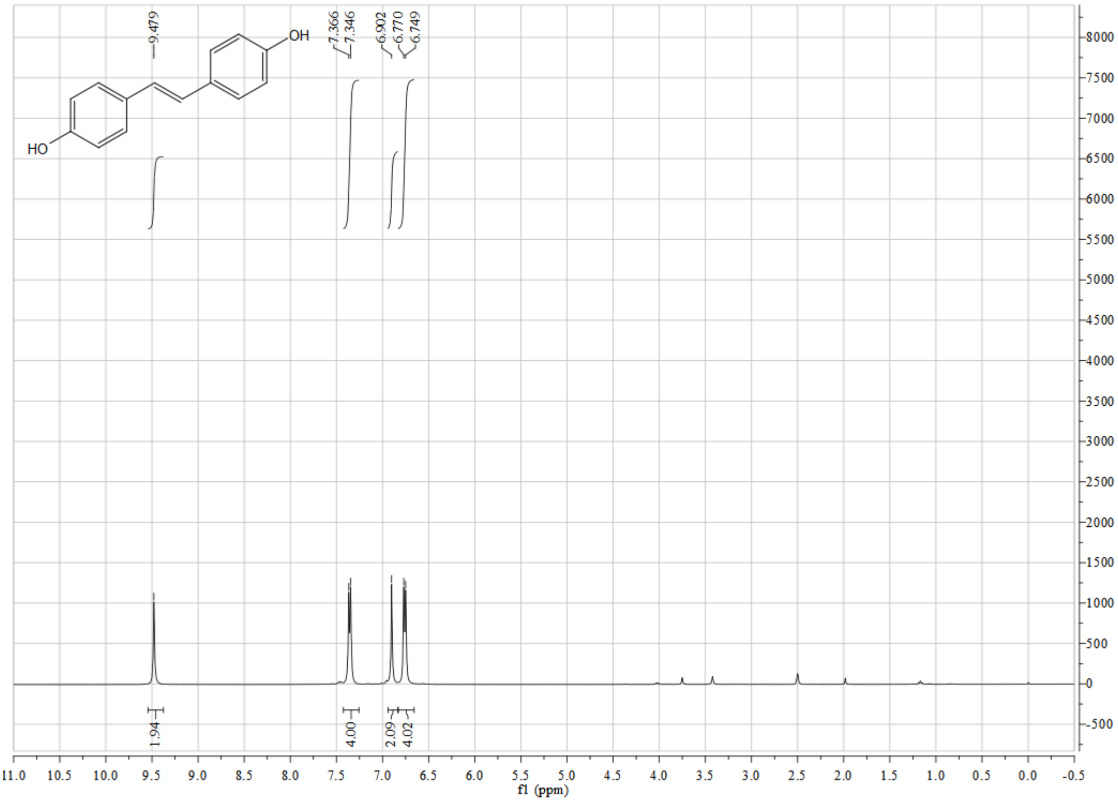

(up) ^{1}H NMR spectrum of compound **2-1** (400 MHz, DMSO-d_6)

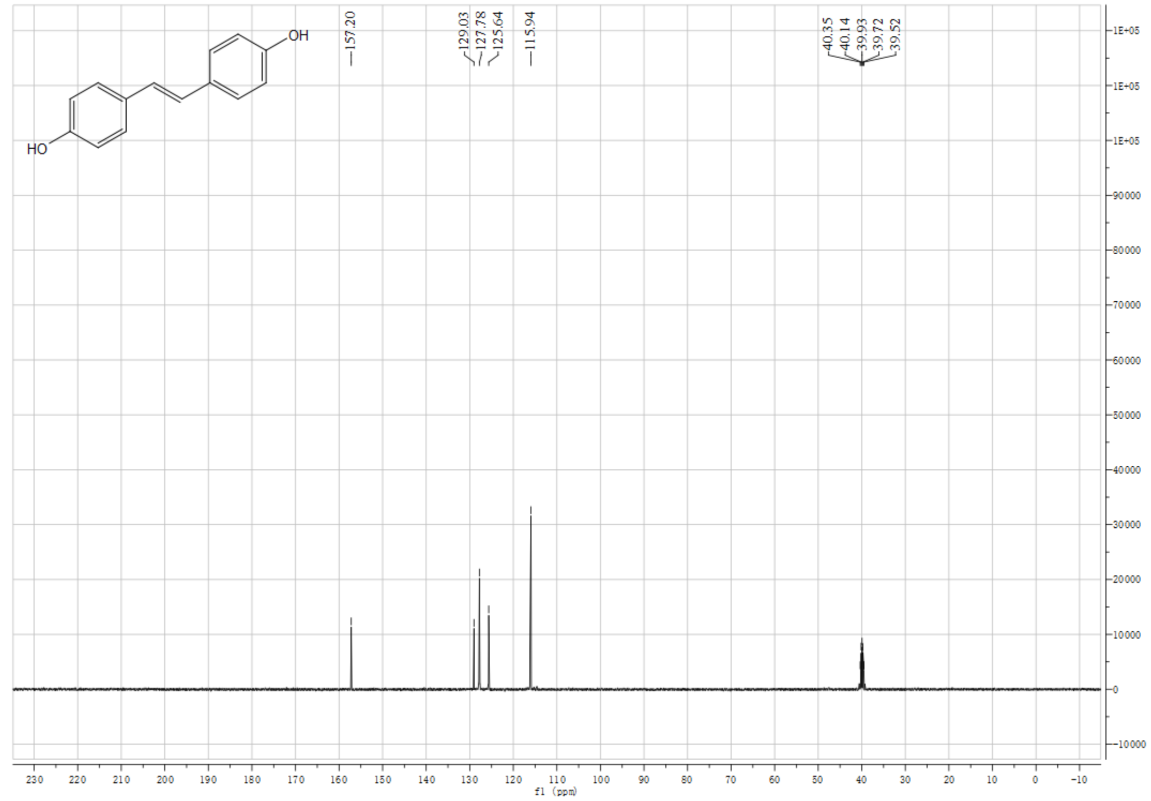

(up) ^{13}C NMR spectrum of compound **2-1** (101 MHz, DMSO-d_6)

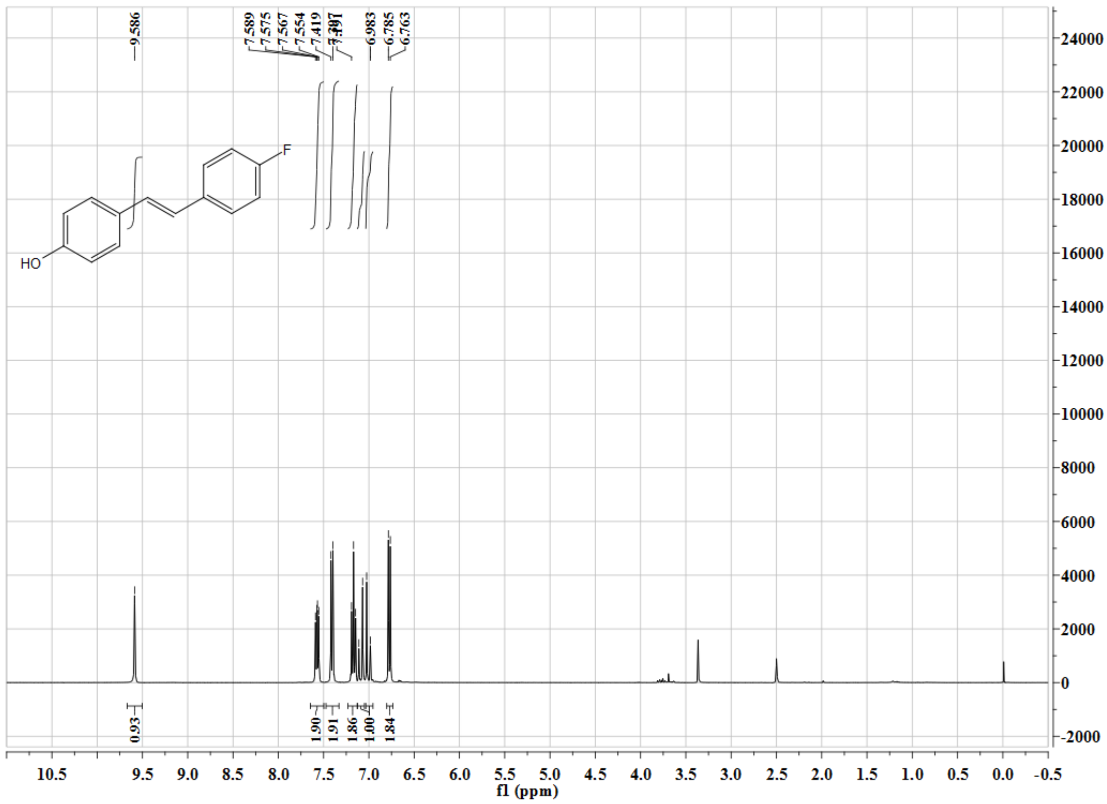

(up) ^{1}H NMR spectrum of compound **2-2** (400 MHz, DMSO-d_6)

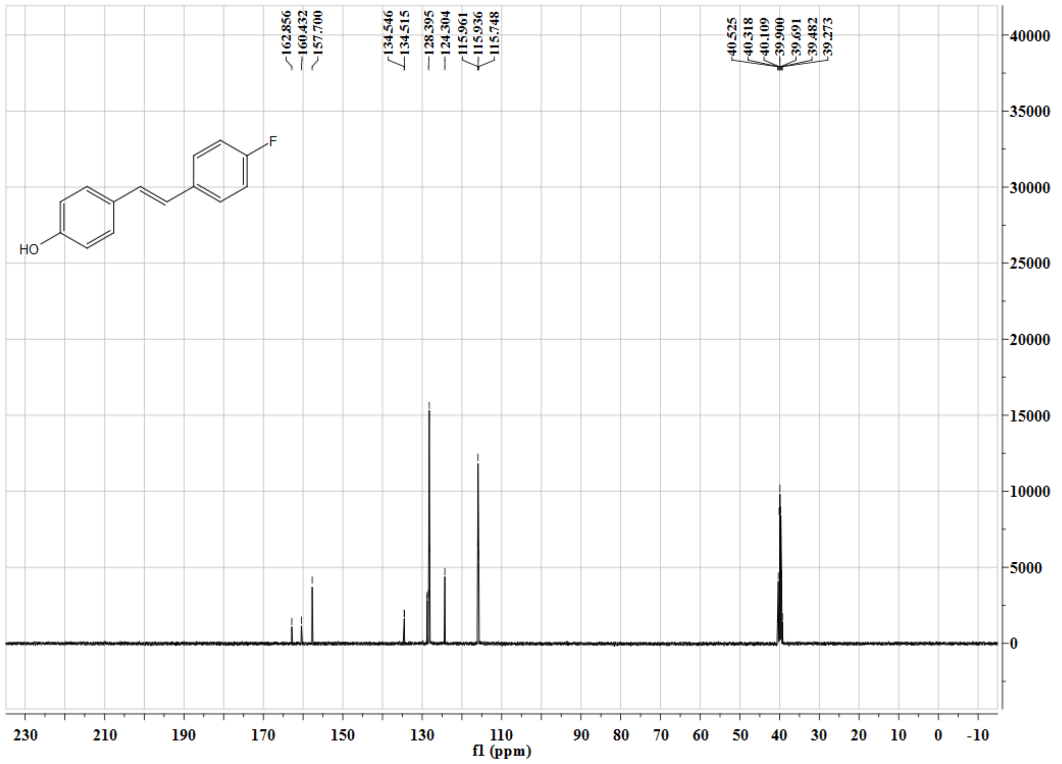

(up) ^{13}C NMR spectrum of compound **2-2** (101 MHz, DMSO-d_6)

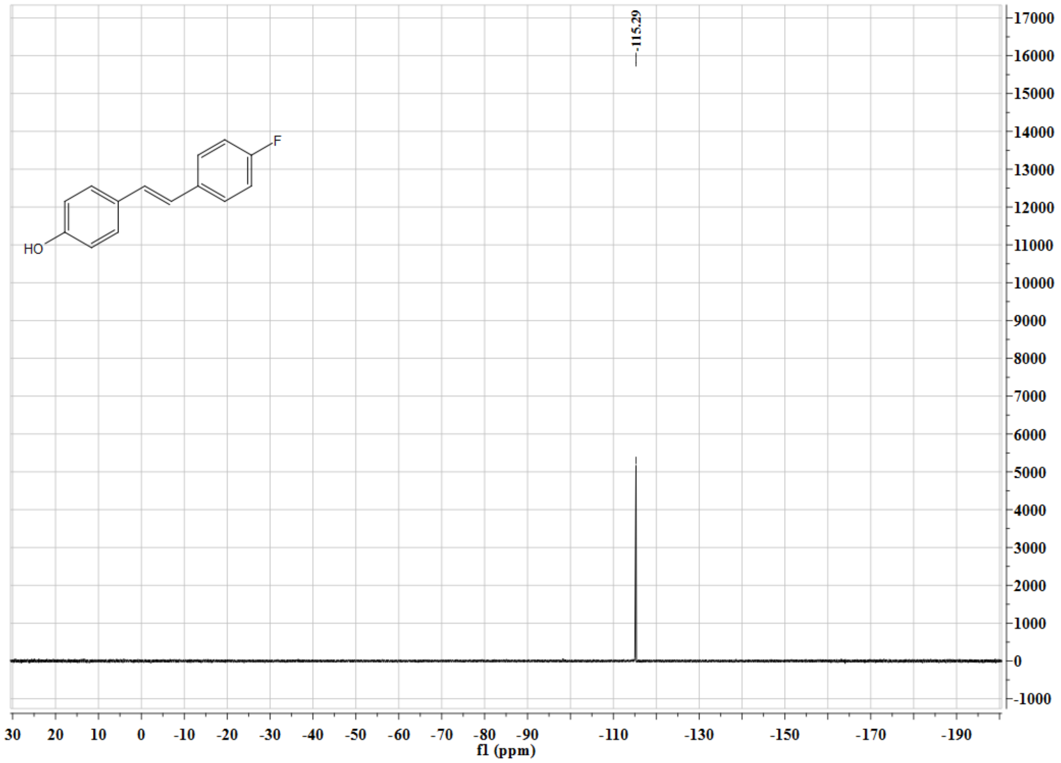

(up) ^{19}F NMR spectrum of compound **2-2** (400 MHz, DMSO-d_6)

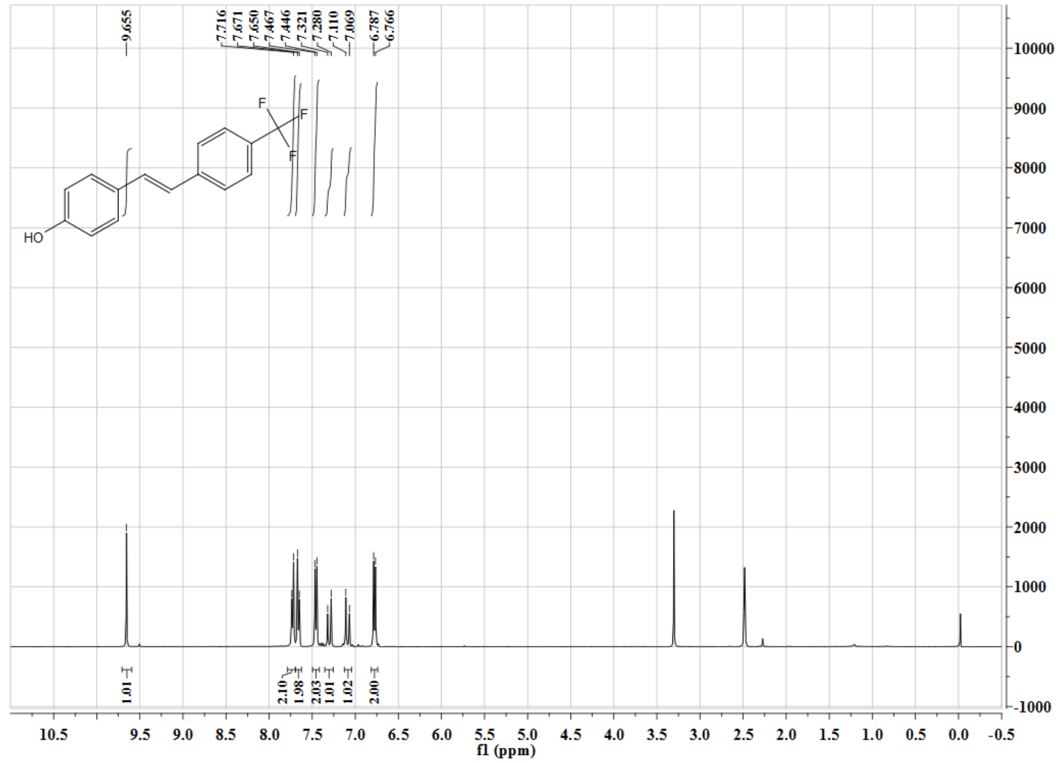

(up) ^{1}H NMR spectrum of compound **2-3** (400 MHz, DMSO-d_6)

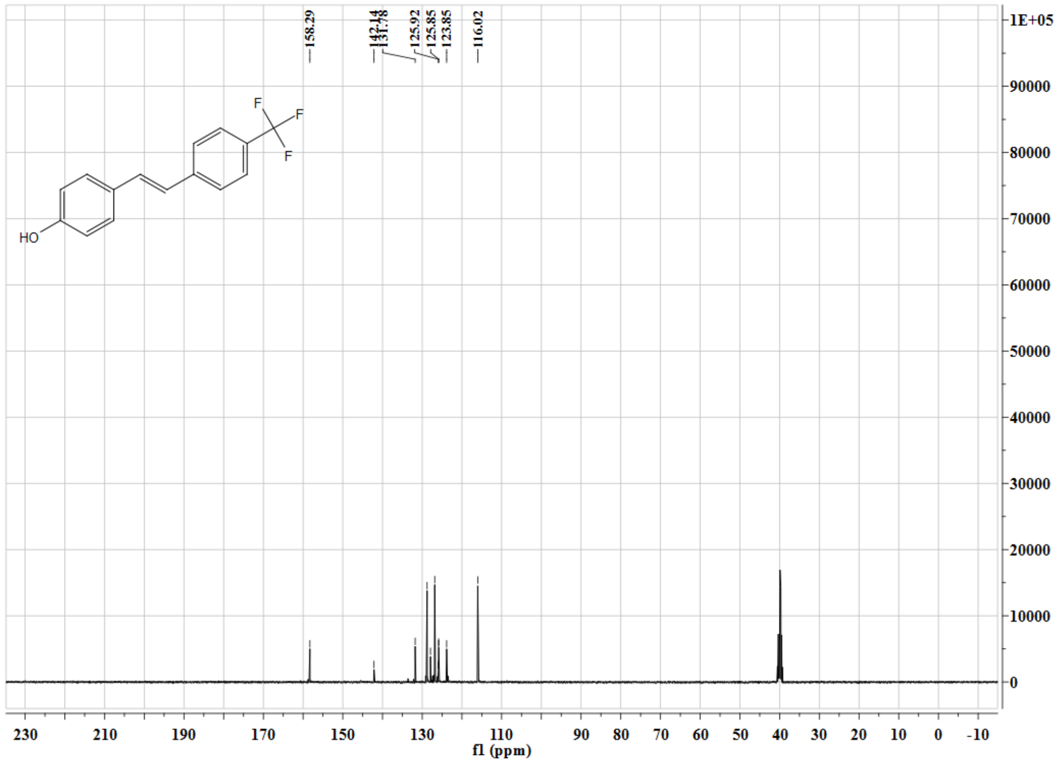

(up) ^{13}C NMR spectrum of compound **2-3** (101 MHz, DMSO-d_6)

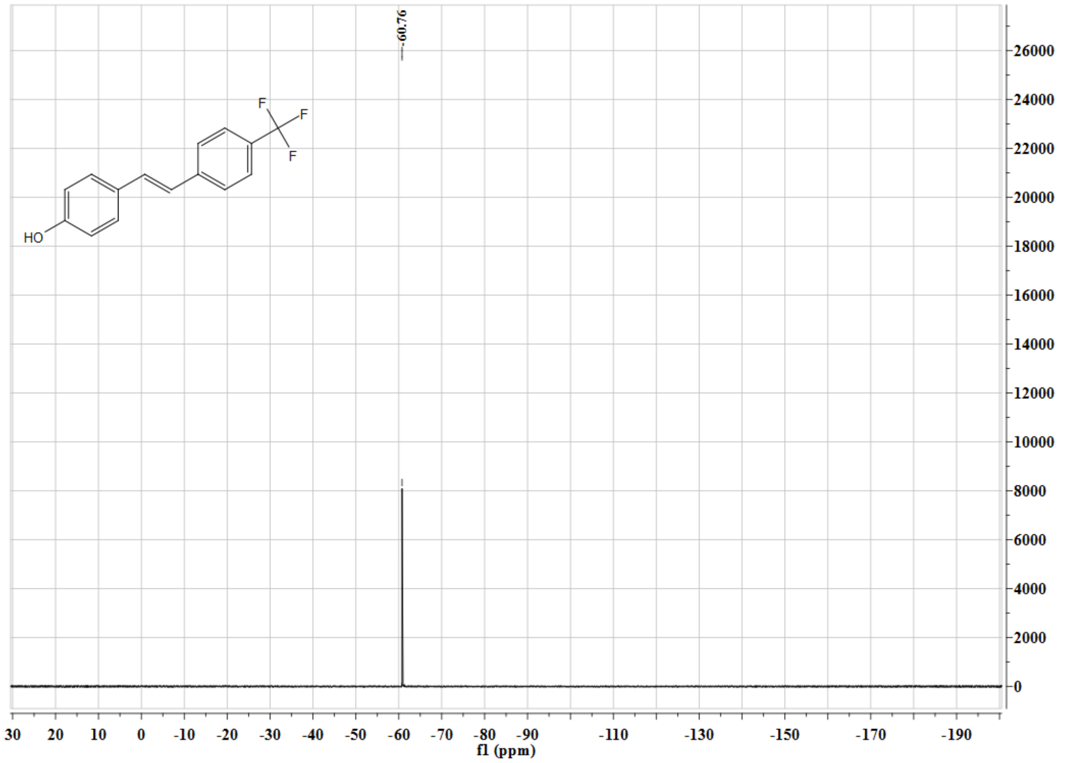

(up) ^{19}F NMR spectrum of compound **2-3** (400 MHz, DMSO-d_6)

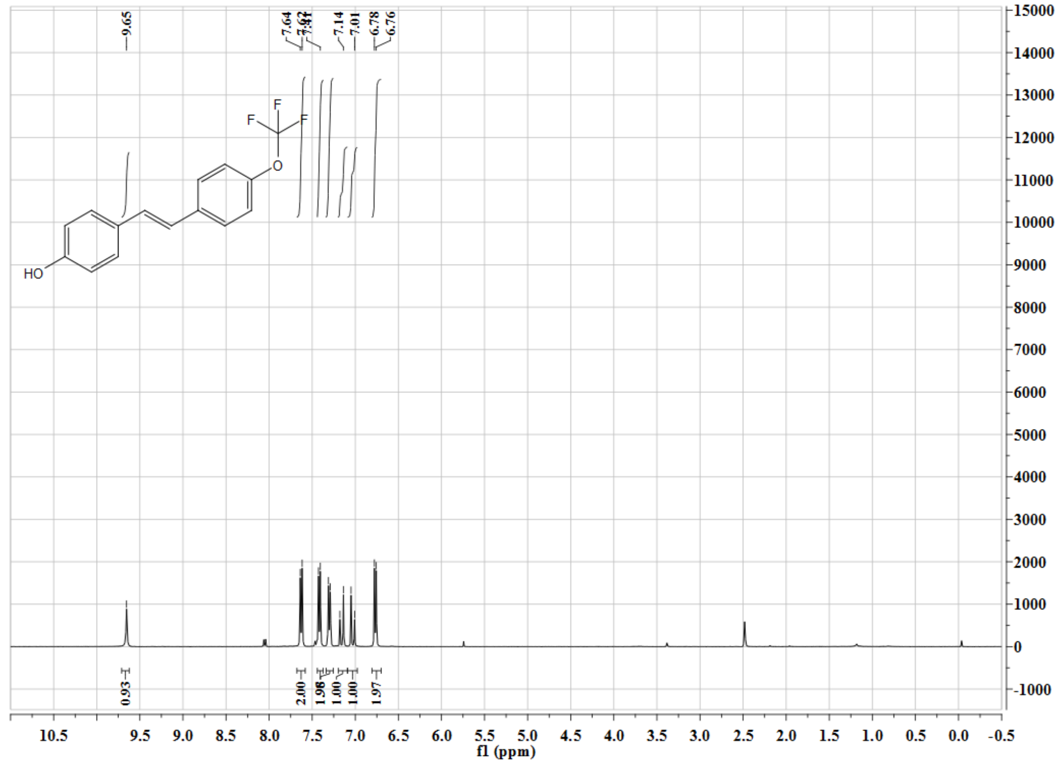

.(up) ^{1}H NMR spectrum of compound **2-4** (400 MHz, DMSO-d_6)

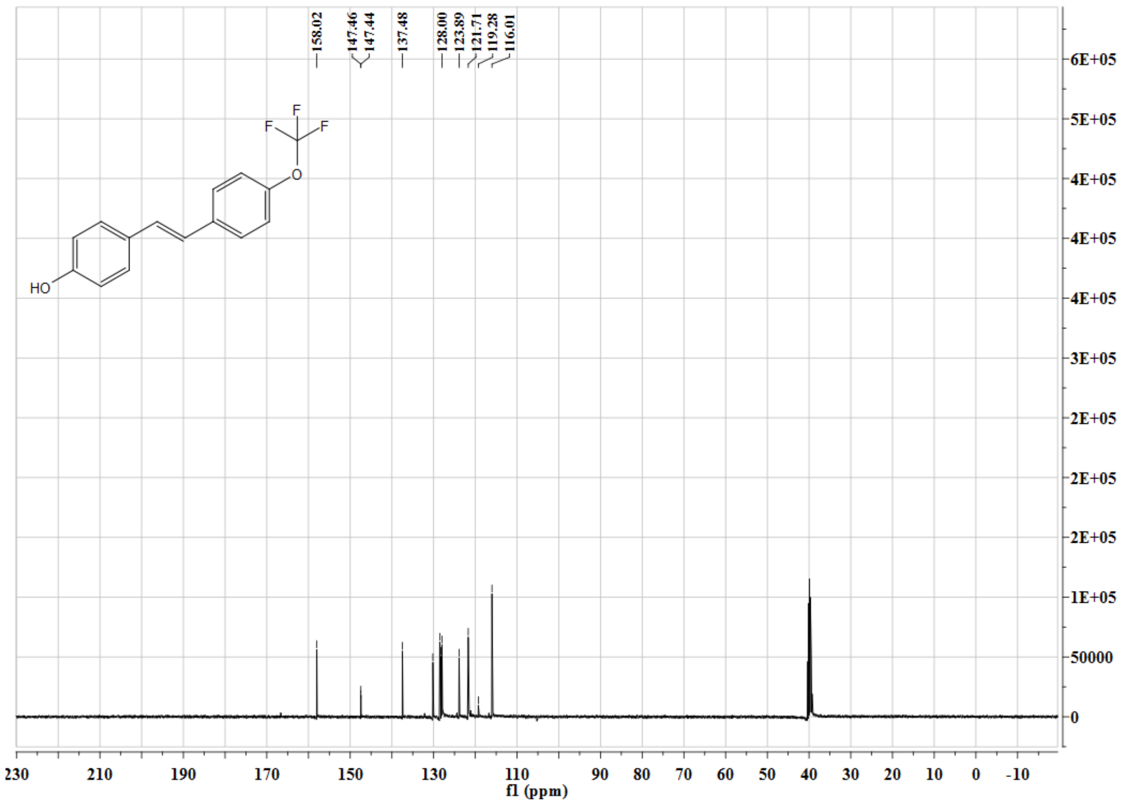

(up) ^{13}C NMR spectrum of compound **2-4** (101 MHz, DMSO-d_6)

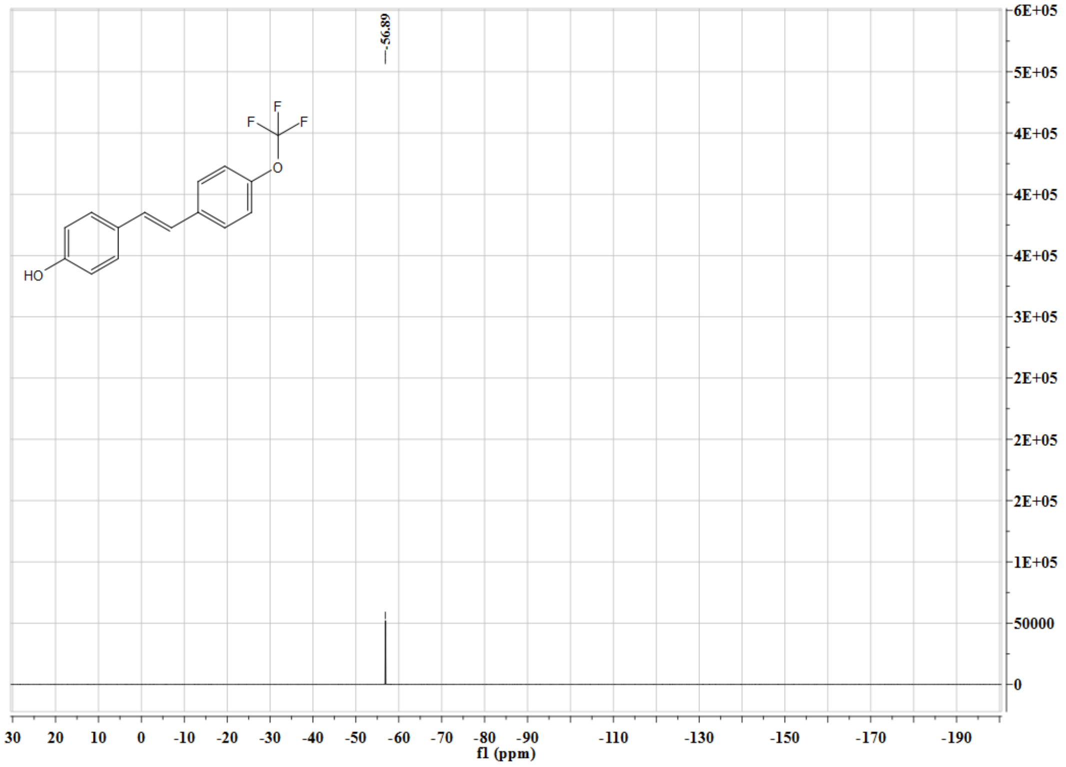

(up) ^{19}F NMR spectrum of compound **2-4** (400 MHz, DMSO-d_6)

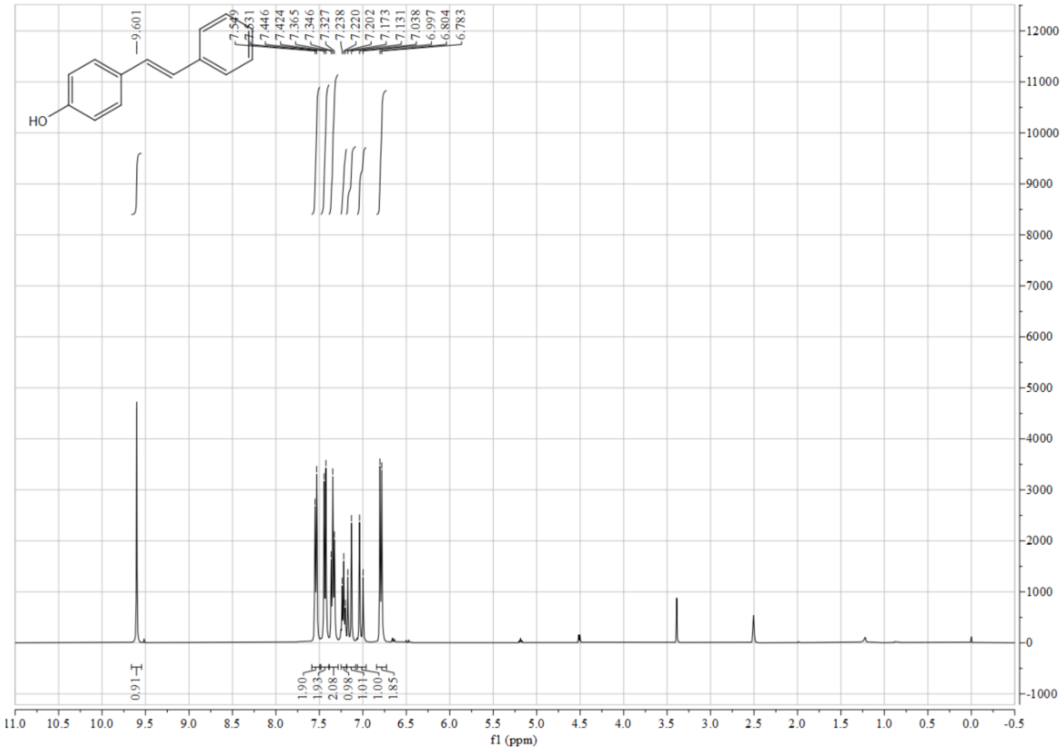

(up) ^{1}H NMR spectrum of compound **2-5** (400 MHz, DMSO-d_6)

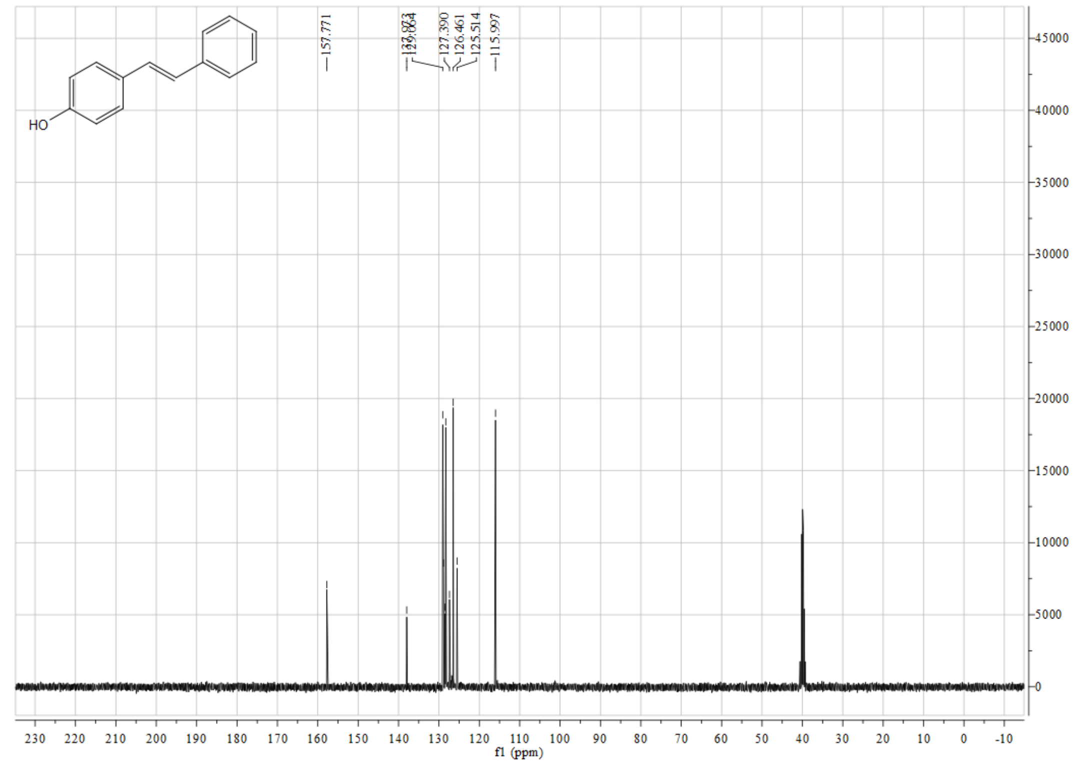

(up) ^{13}CNMR spectrum of compound **2-5** (101 MHz, DMSO-d_6)

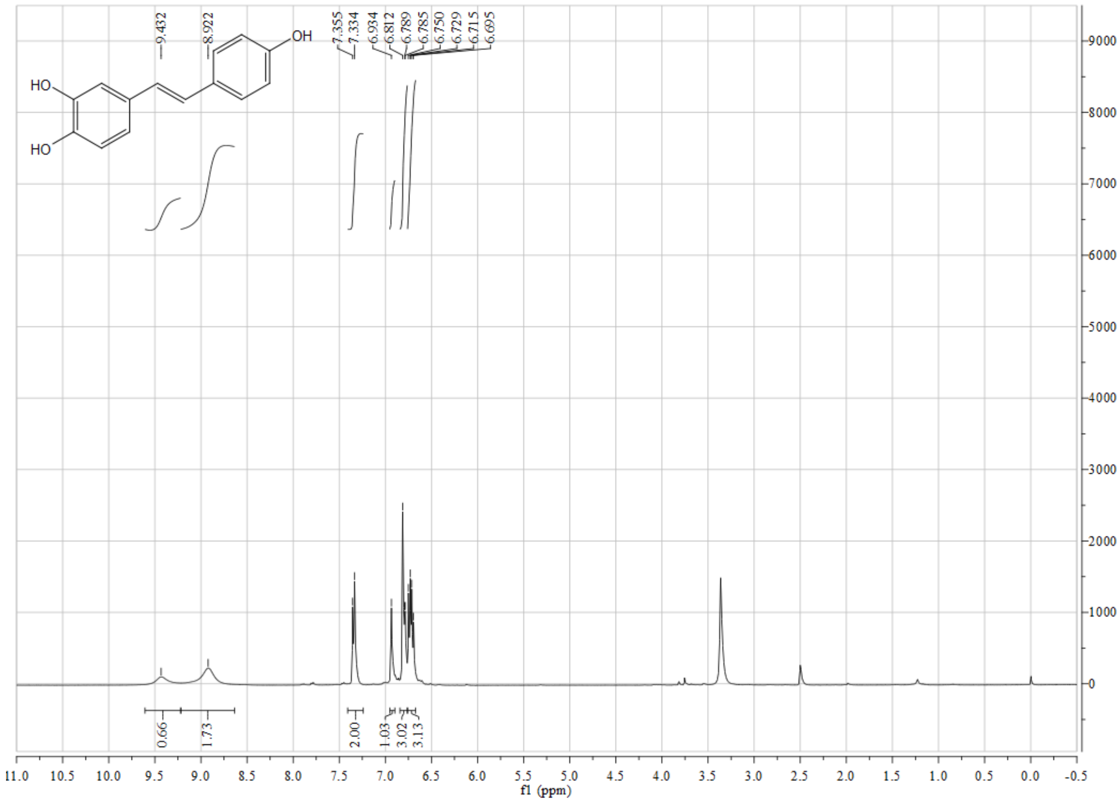

(up) ^{1}H NMR spectrum of compound **3-1** (400 MHz, DMSO-d_6)

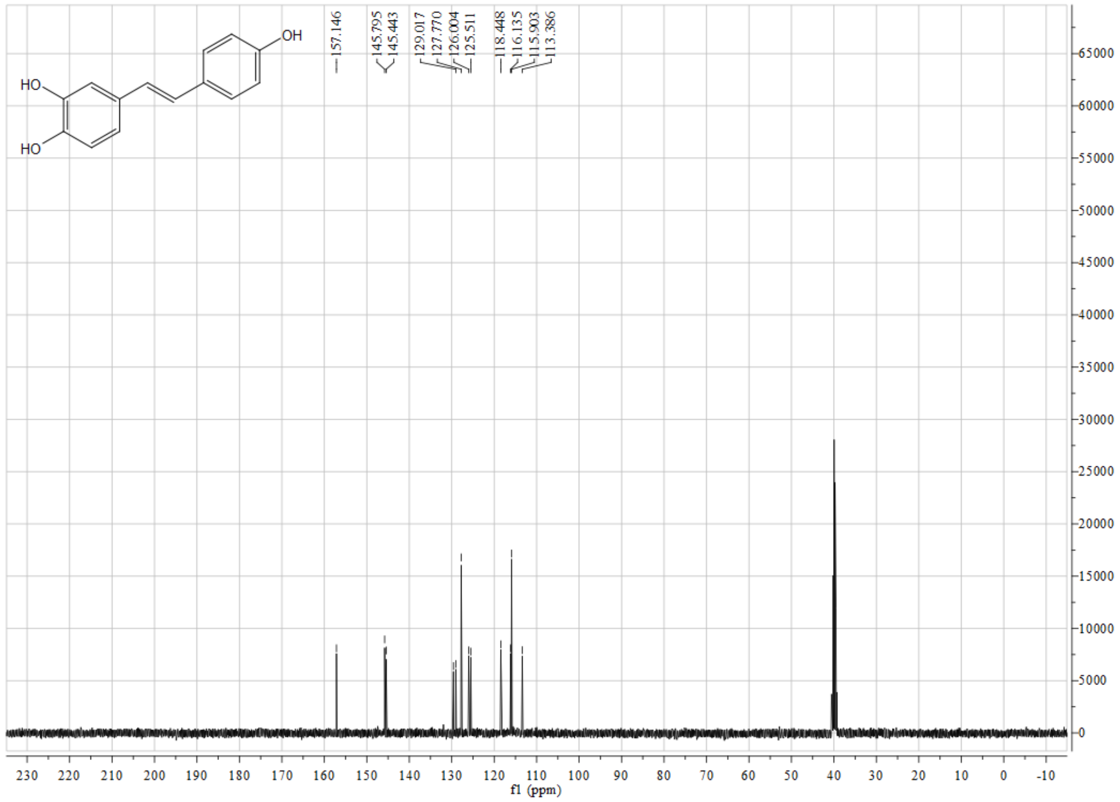

(up) ^{13}C NMR spectrum of compound **3-1** (101 MHz, DMSO-d_6)

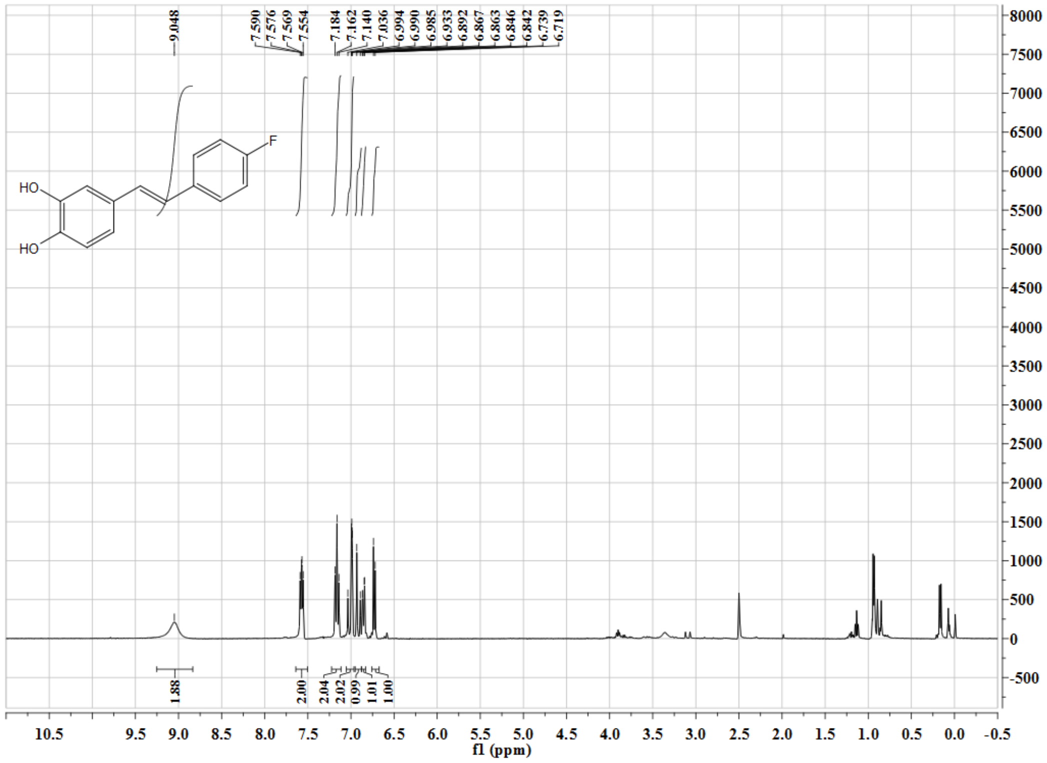

(up) ^{1}H NMR spectrum of compound **3-2** (400 MHz, DMSO-d_6)

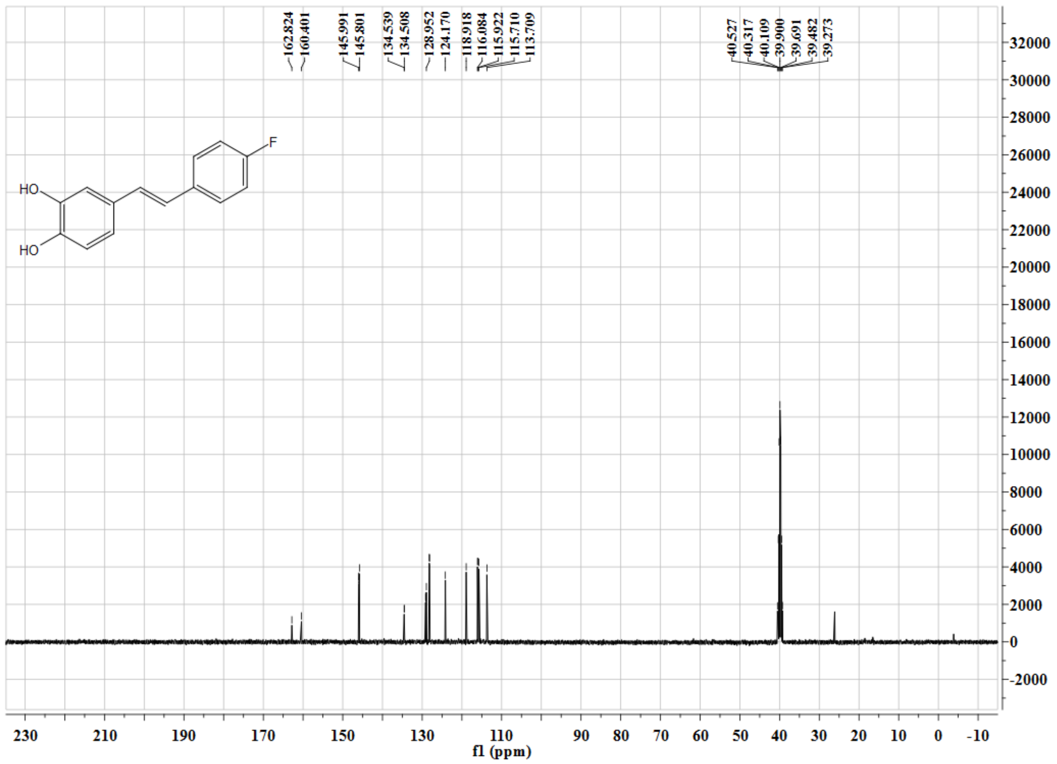

(up) ^{13}C NMR spectrum of compound **3-2** (101 MHz, DMSO-d_6)

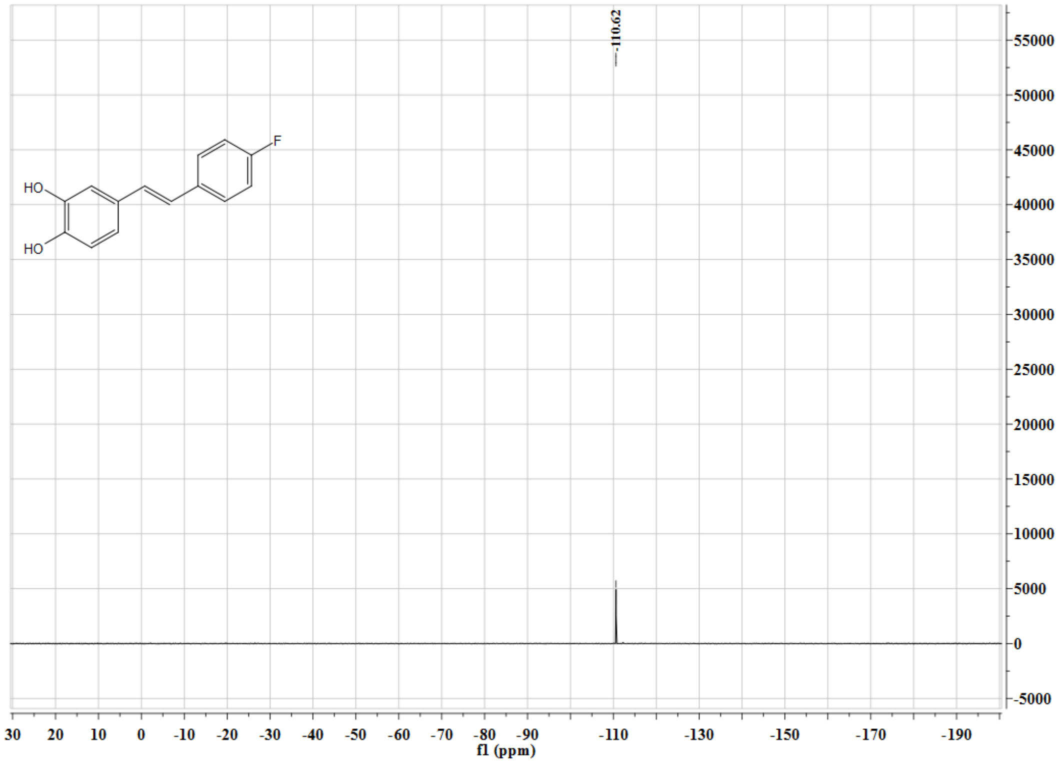

(up) ^{19}F NMR spectrum of compound **3-2** (400 MHz, DMSO-d_6)

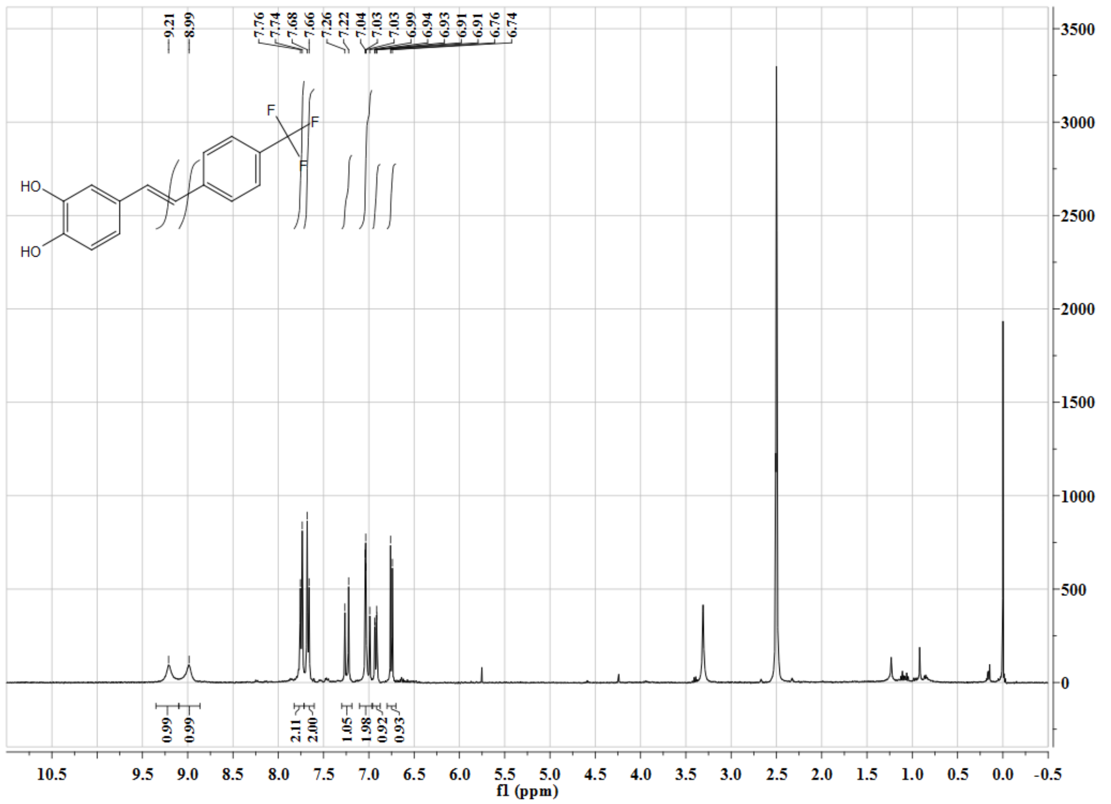

(up) ^{1}H NMR spectrum of compound **3-3** (400 MHz, DMSO-d_6)

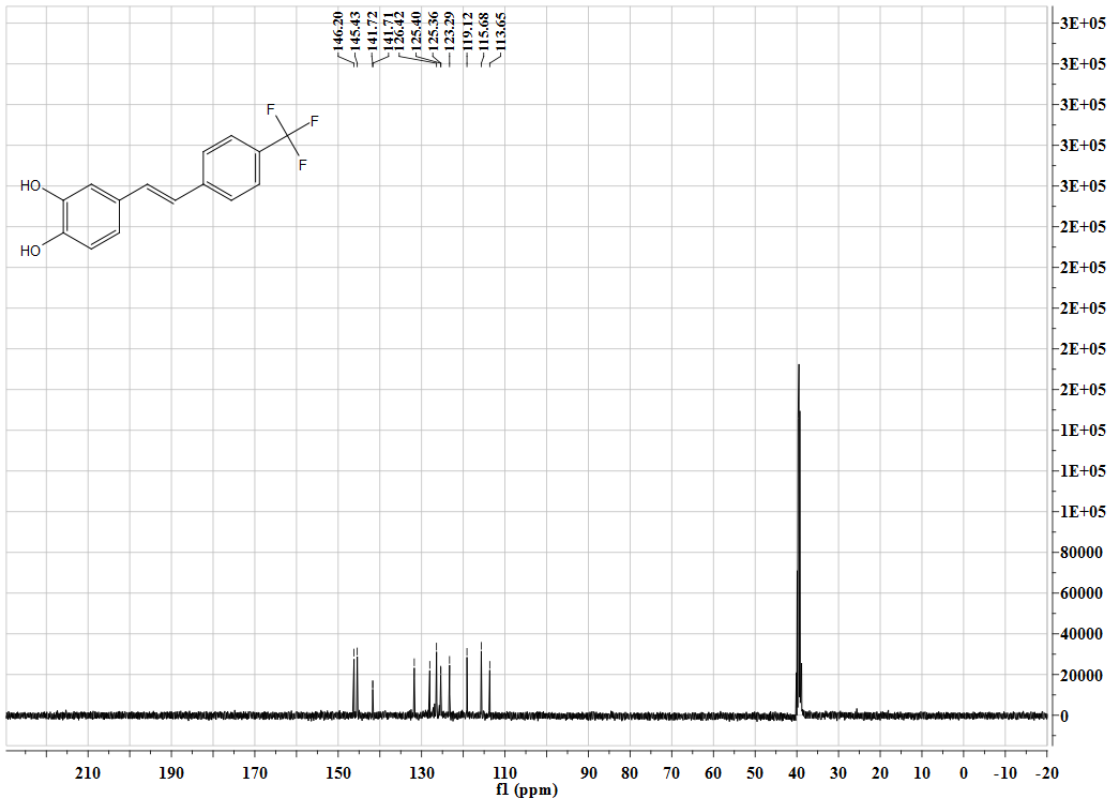

(up) ^{13}C NMR spectrum of compound **3-3** (101 MHz, DMSO-d_6)

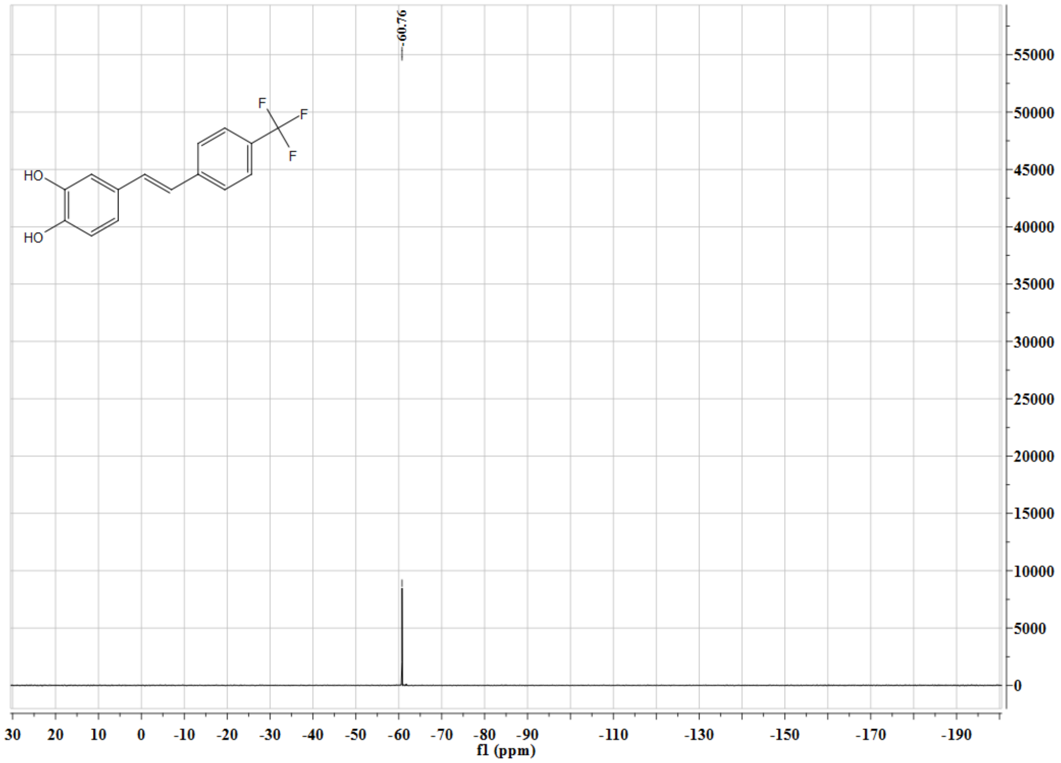

(up) ^{19}F NMR spectrum of compound **3-3** (400 MHz, DMSO-d_6)

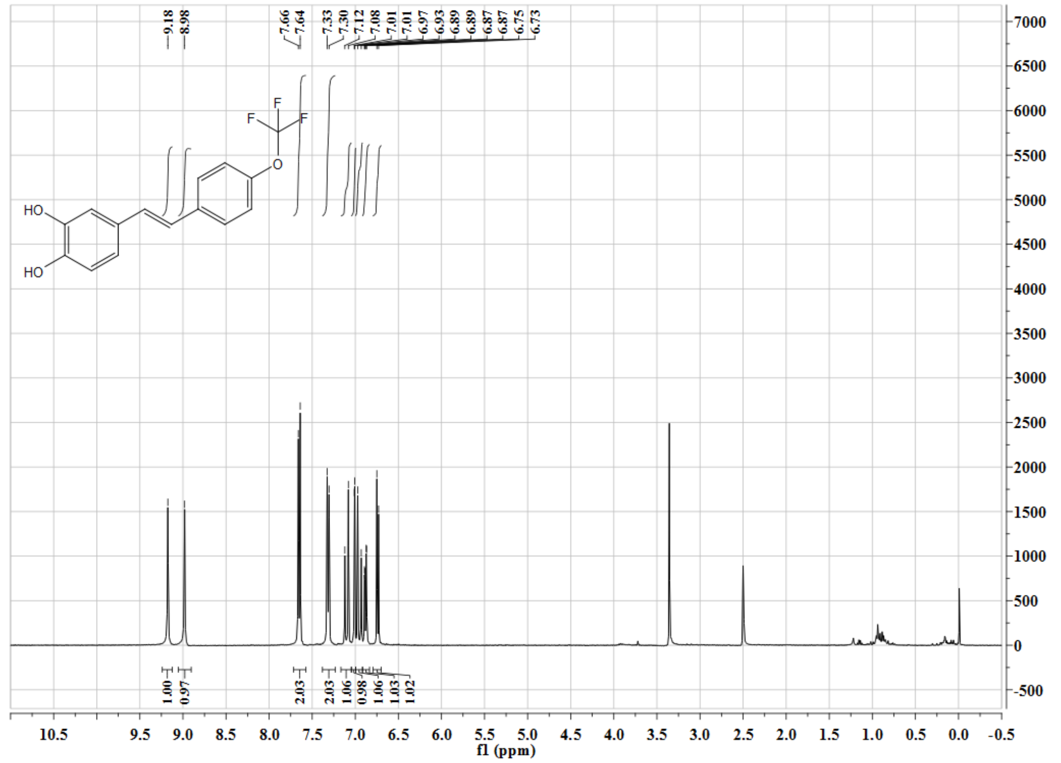

(up) ^{1}H NMR spectrum of compound **3-4** (400 MHz, DMSO-d_6)

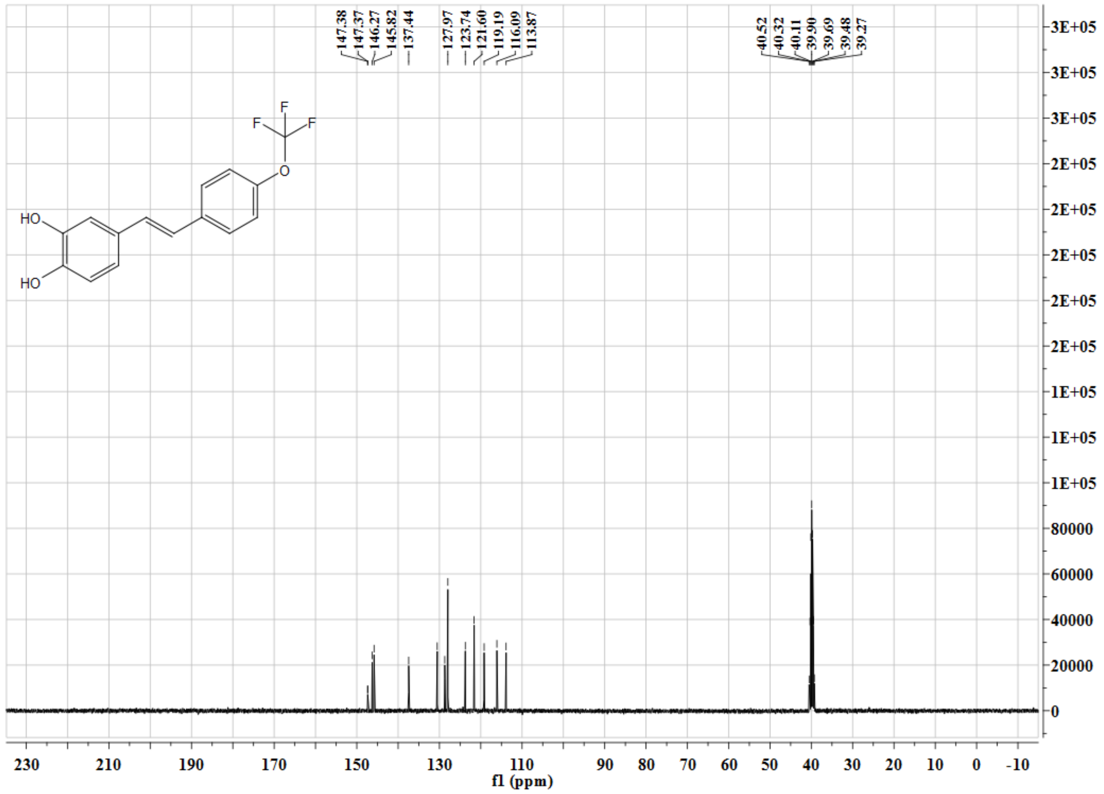

(up) ^{13}CNMR spectrum of compound **3-4** (101 MHz, DMSO-d_6)

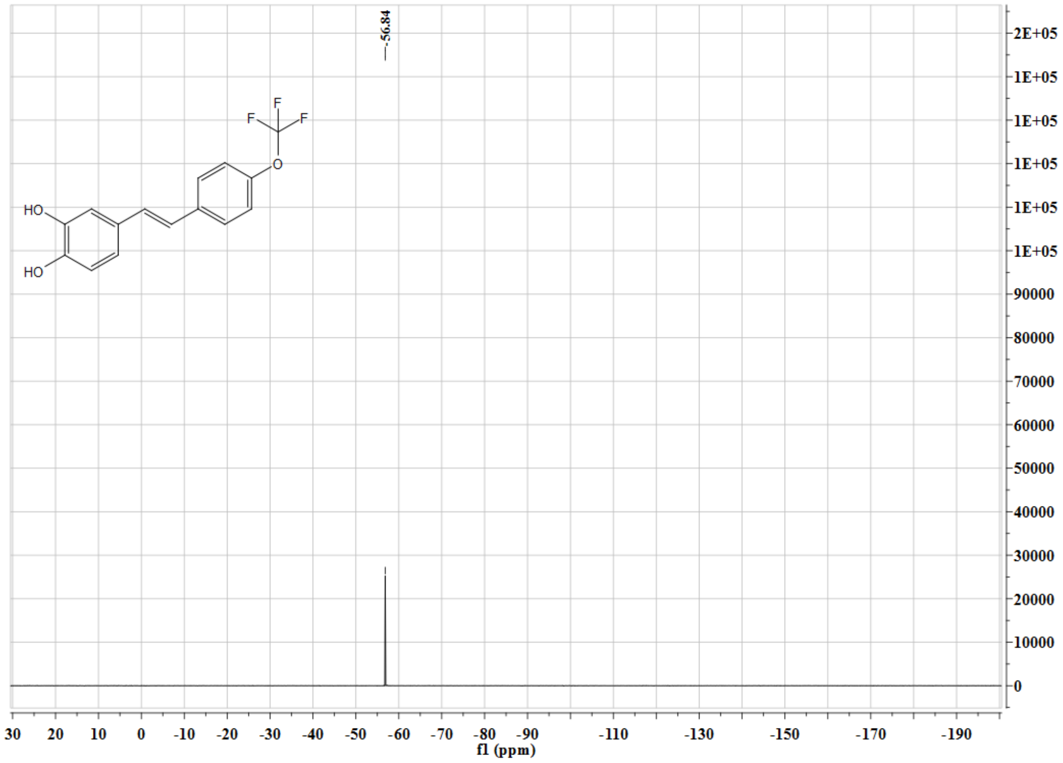

(up) ^{19}F NMR spectrum of compound **3-4** (400 MHz, DMSO-d_6)

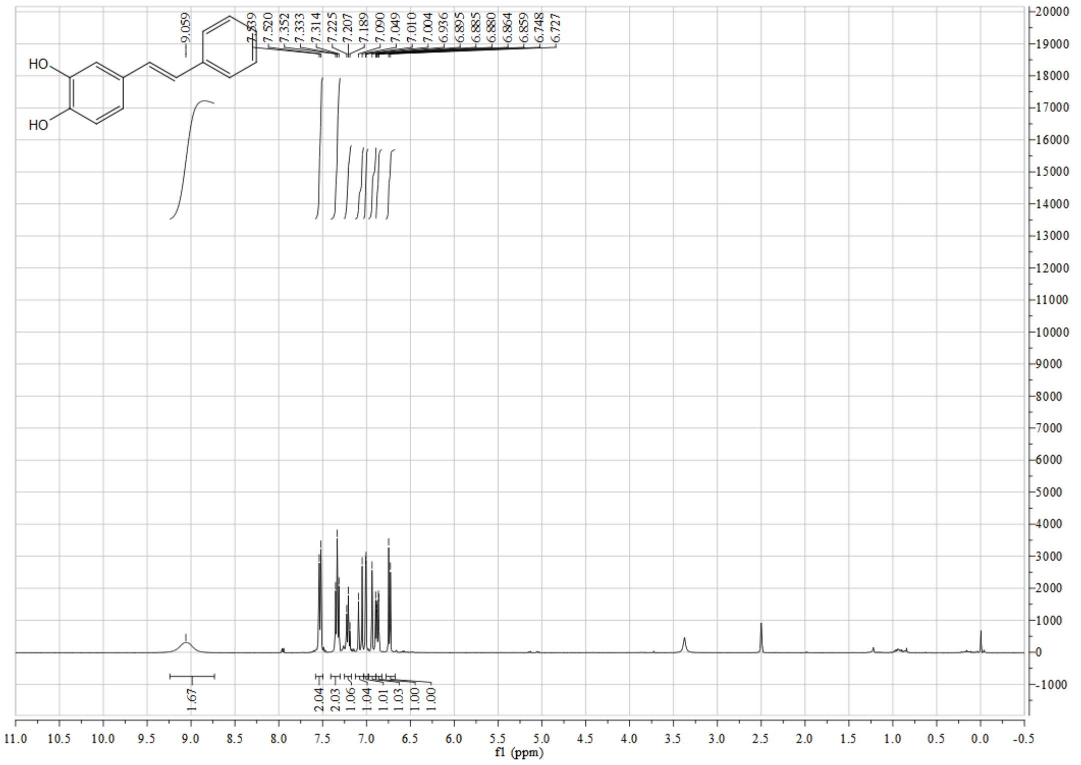

(up) ^{1}H NMR spectrum of compound **3-5** (400 MHz, DMSO-d_6)

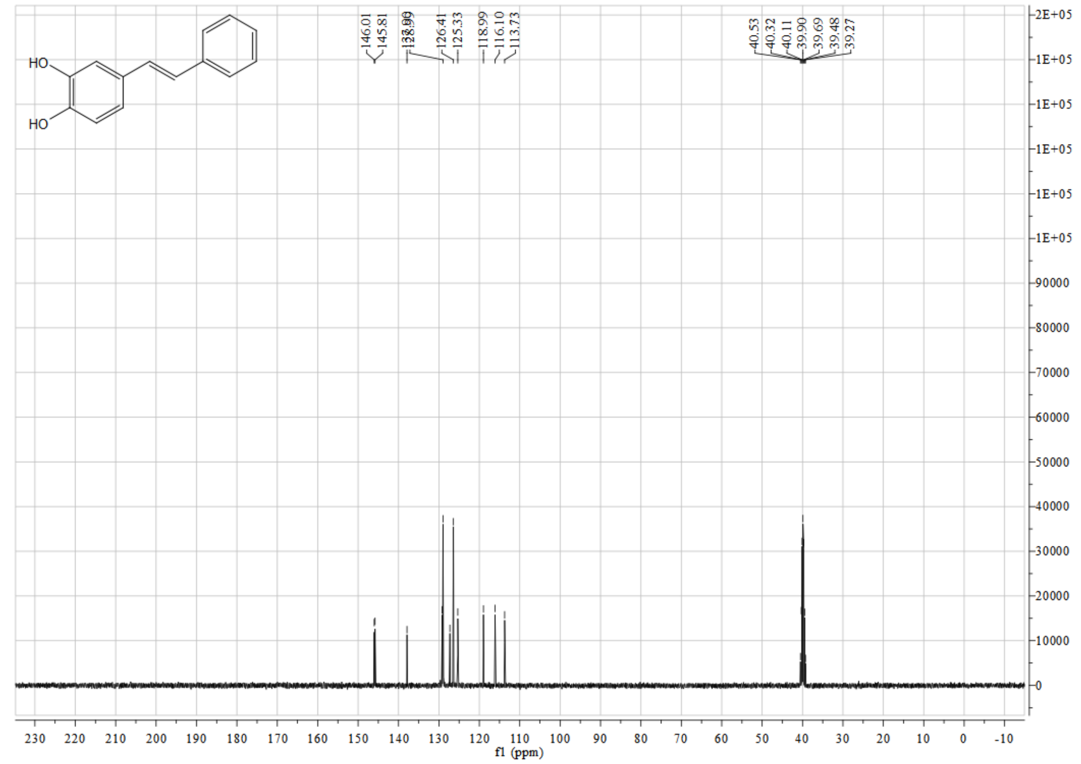

(up) ^{13}CNMR spectrum of compound **3-5** (101 MHz, DMSO-d_6)

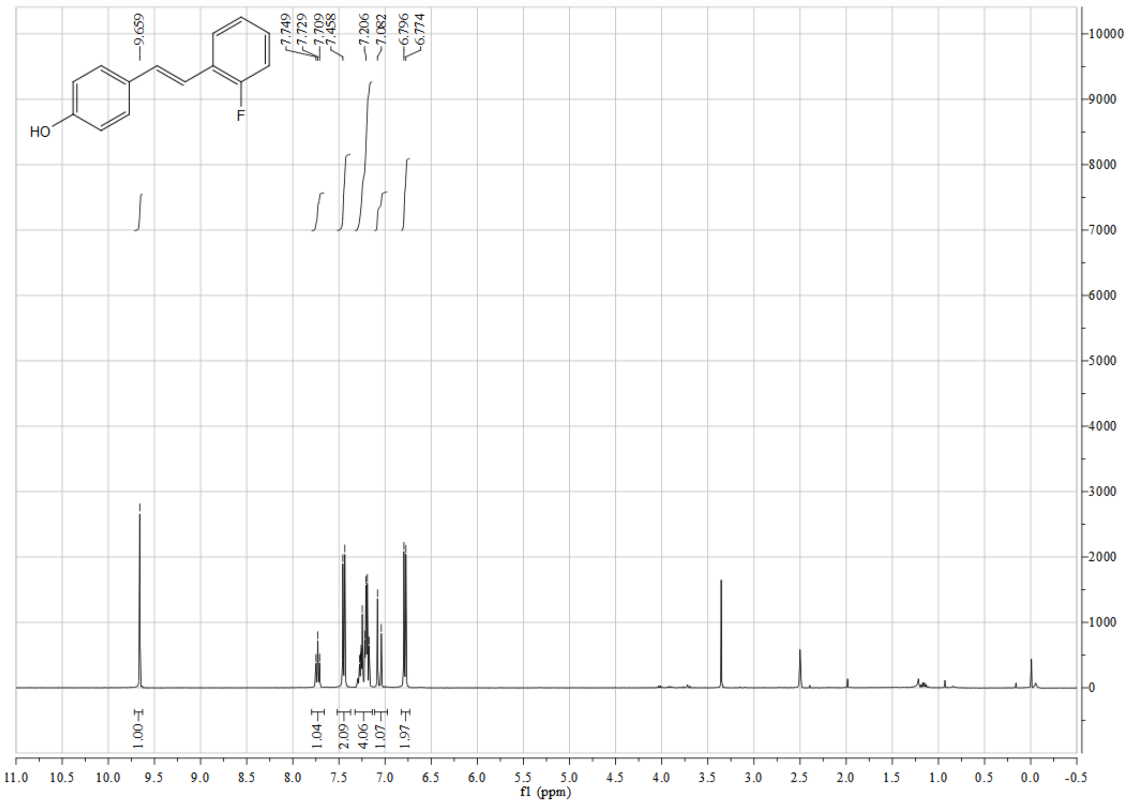

(up) ^{1}H NMR spectrum of compound **4-1** (400 MHz, DMSO-d_6)

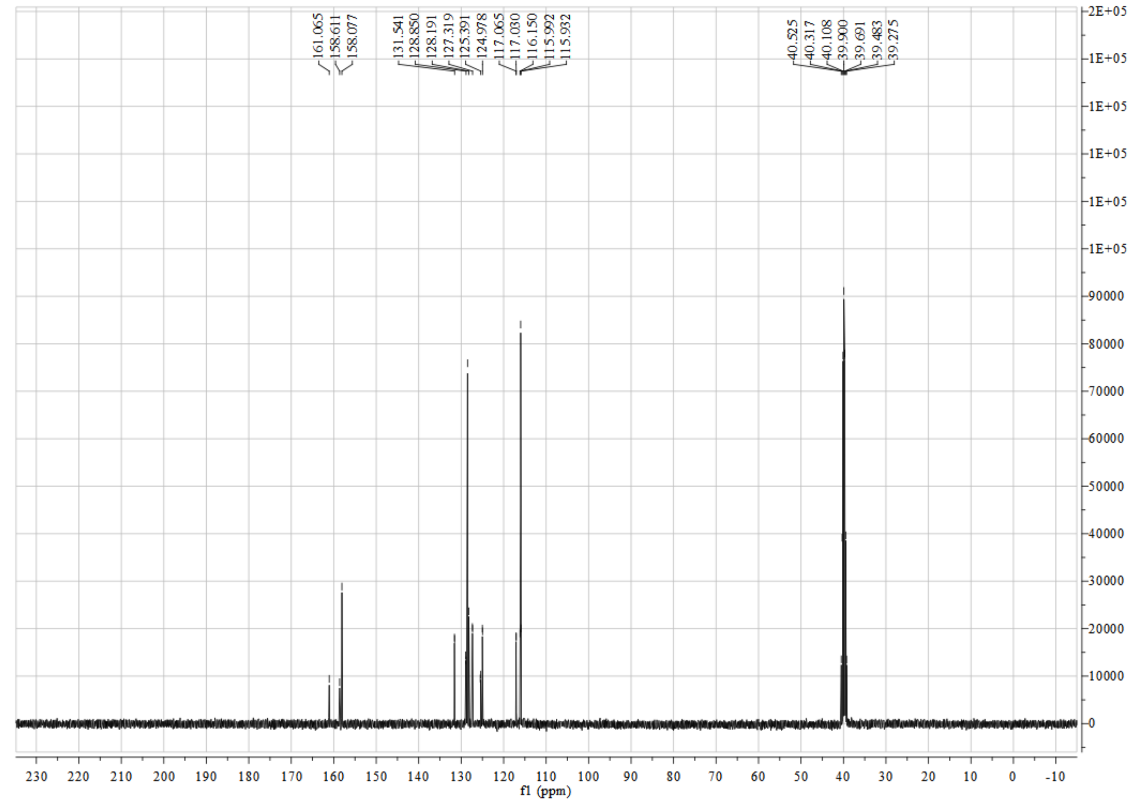

(up) ^{13}C NMR spectrum of compound **4-1** (101 MHz, DMSO-d_6)

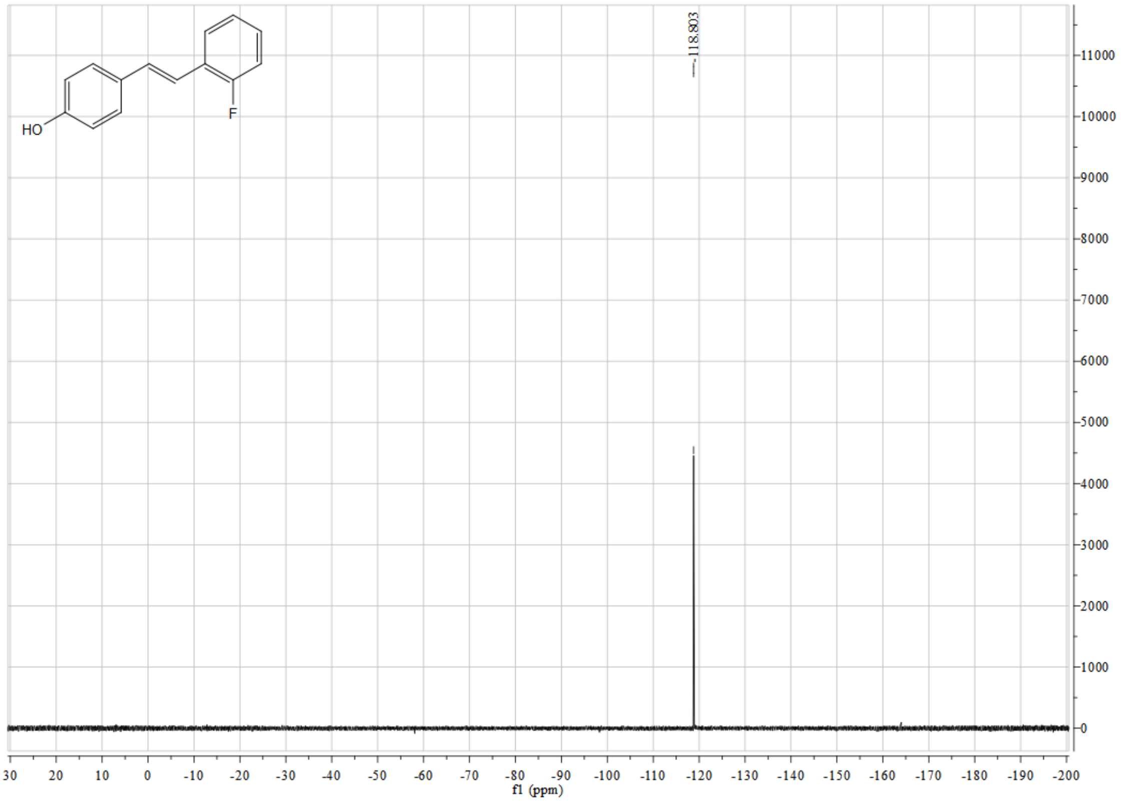

(up) ^{19}F NMR spectrum of compound **4-1** (400 MHz, DMSO-d_6)

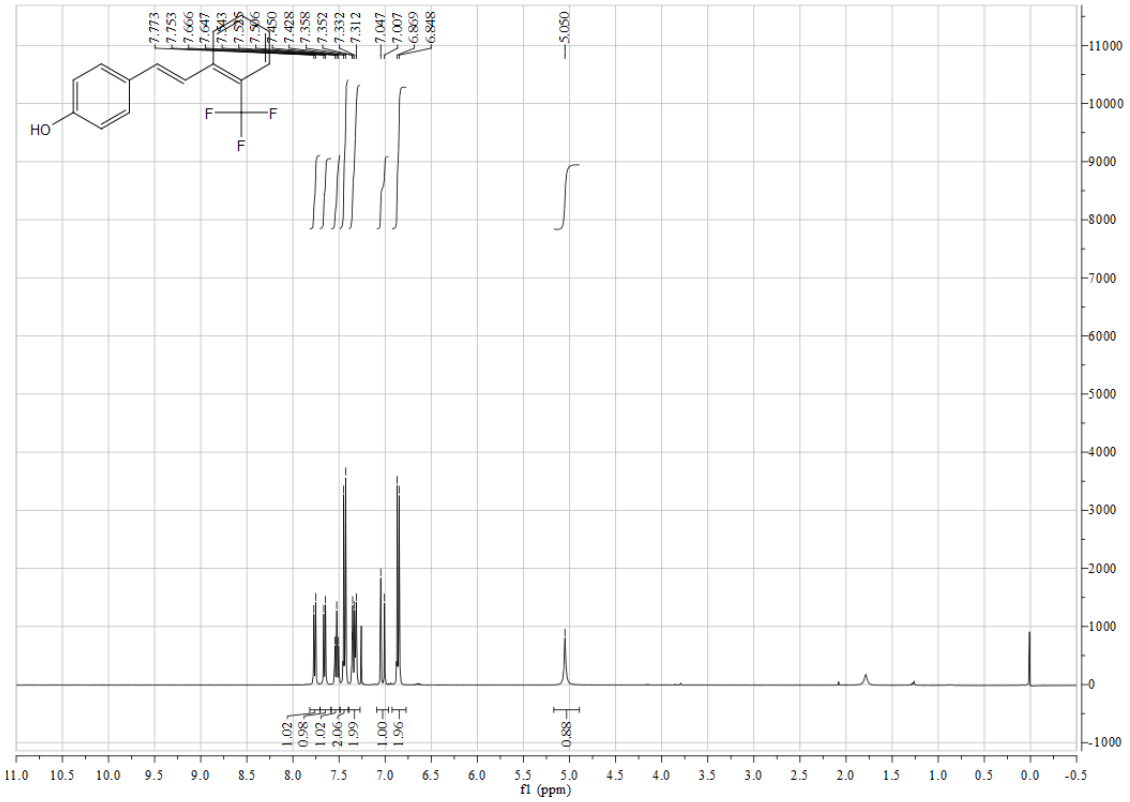

(up) ^{1}H NMR spectrum of compound **4-2** (400 MHz, $CDCl_3$)

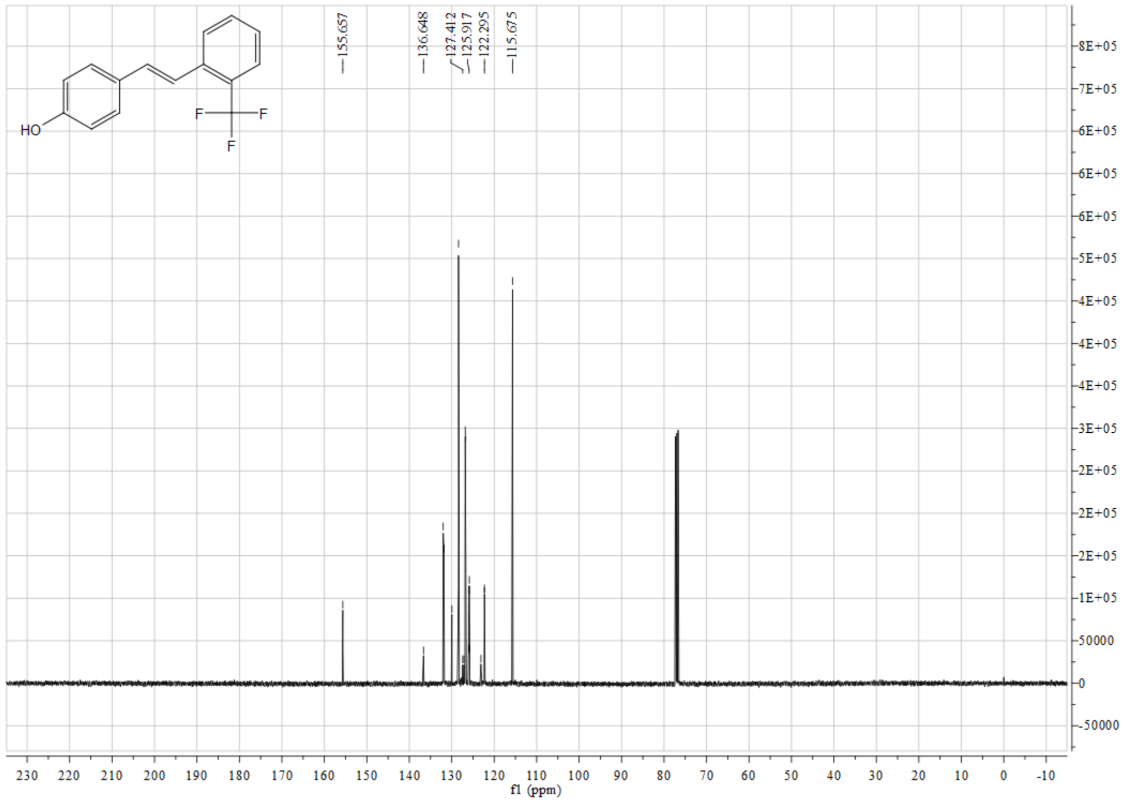

(up) ^{13}C NMR spectrum of compound **4-2** (101 MHz, $CDCl_3$)

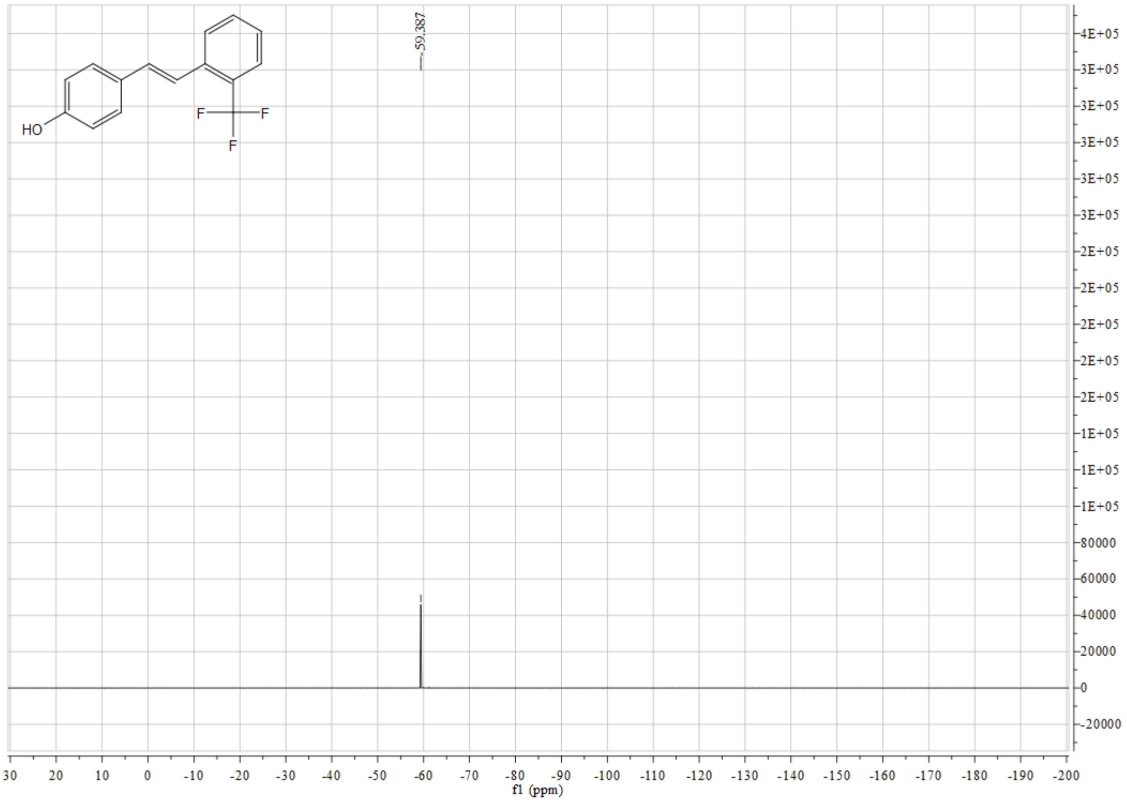

(up) ^{19}F NMR spectrum of compound **4-2** (400 MHz, $CDCl_3$)

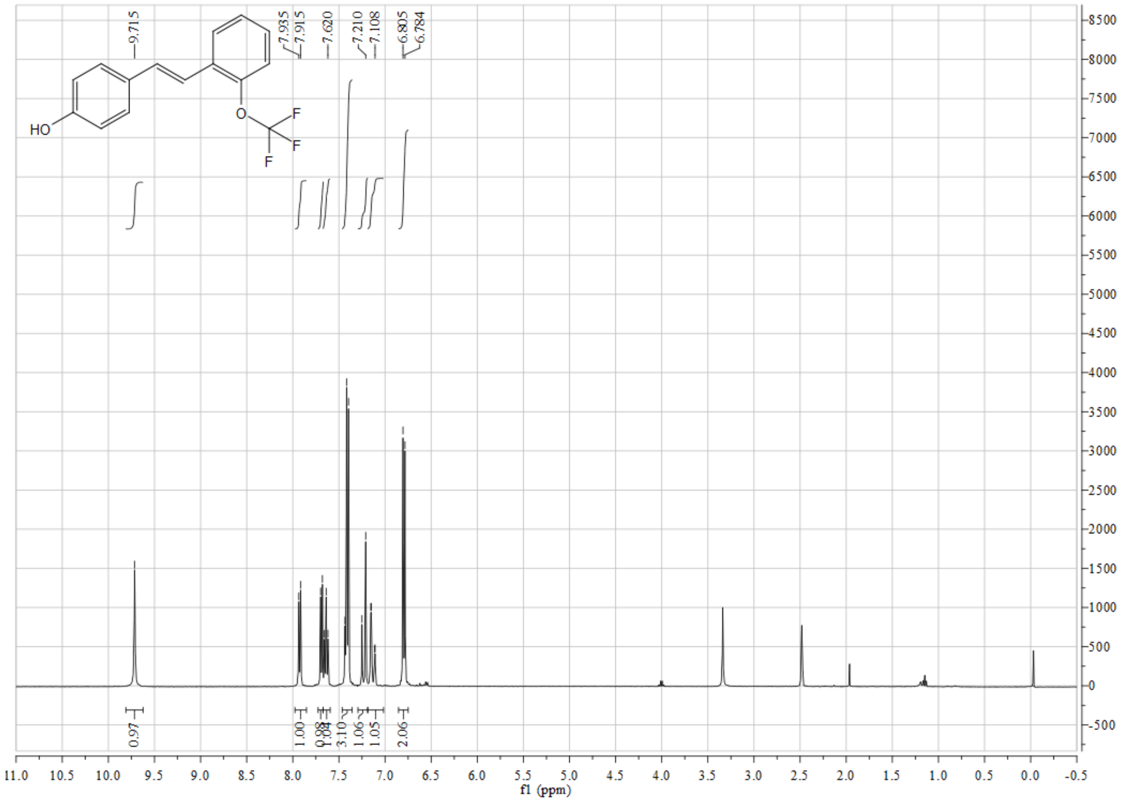

(up) ^{1}H NMR spectrum of compound **4-3** (400 MHz, DMSO-d_6)

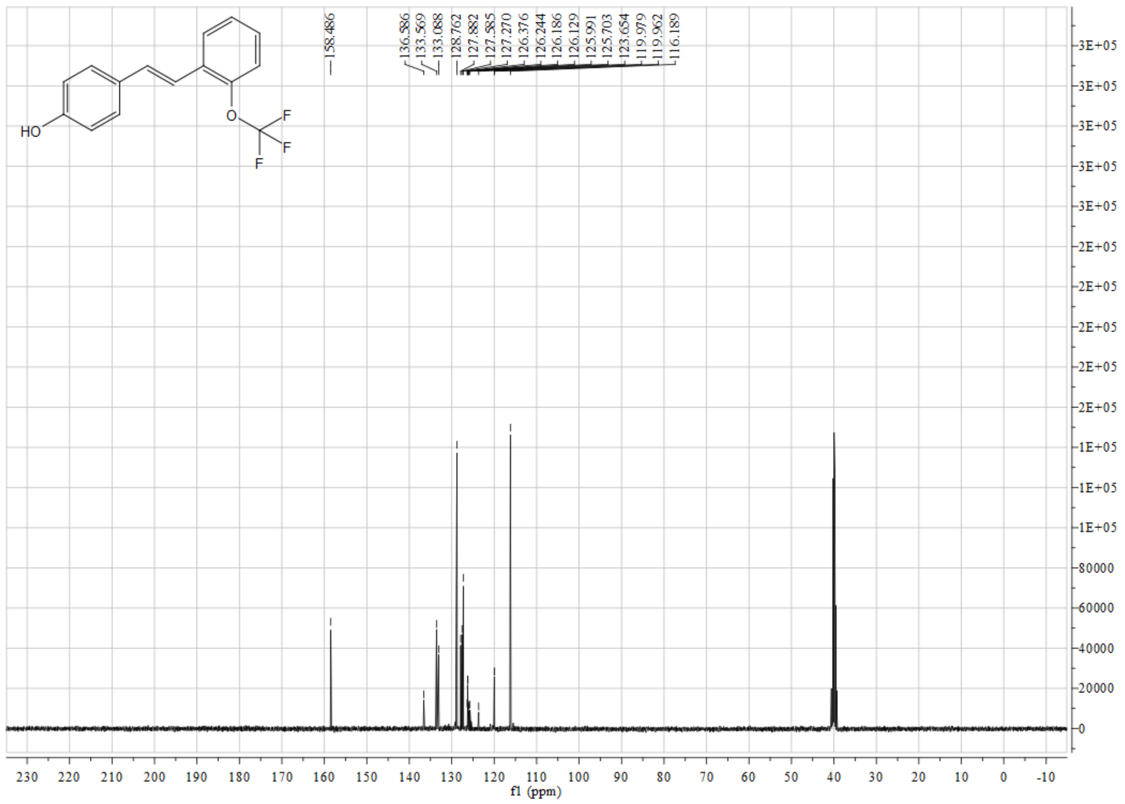

(up) ^{13}C NMR spectrum of compound **4-3** (101 MHz, DMSO-d_6)

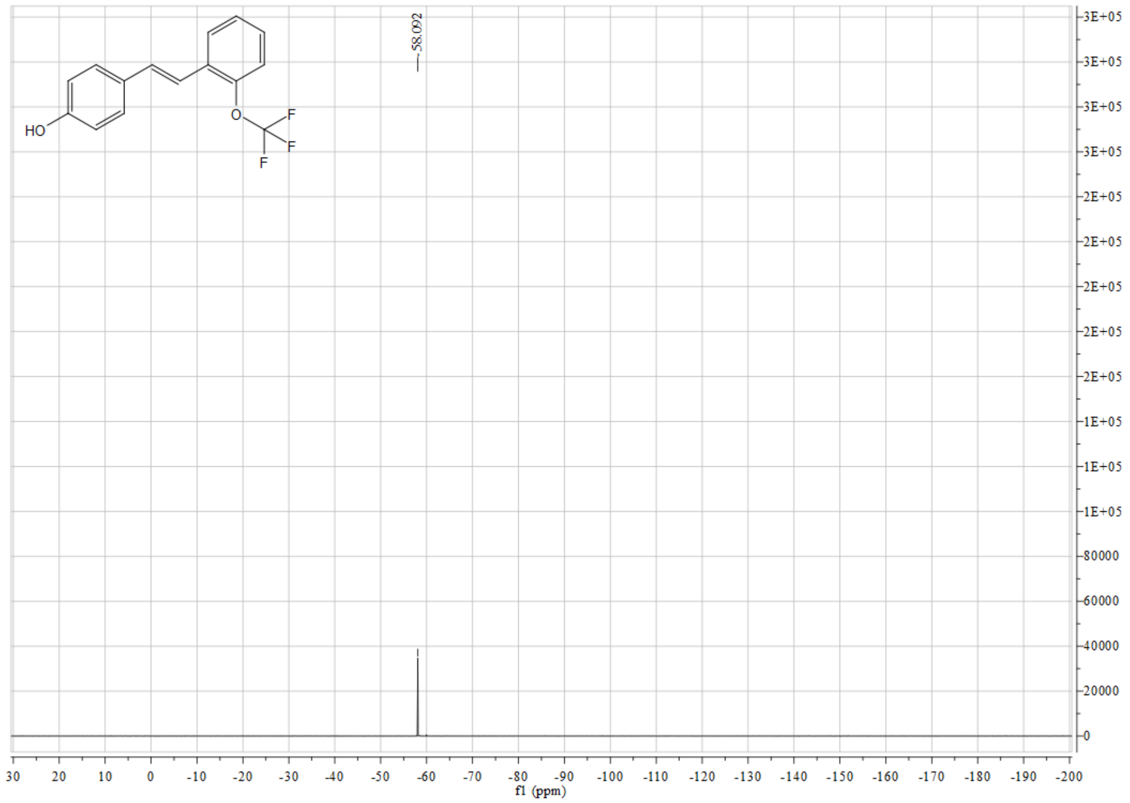

(up) ^{19}F NMR spectrum of compound **4-3** (400 MHz, DMSO-d_6)

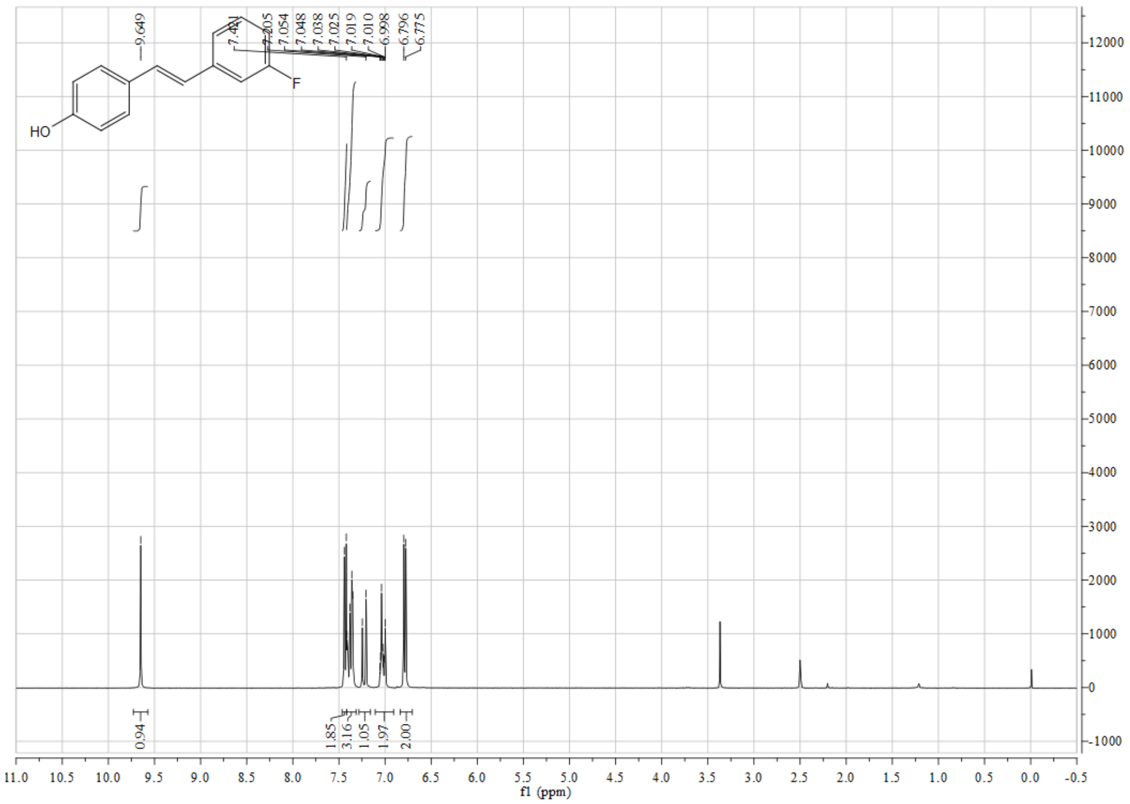

(up) ^{1}H NMR spectrum of compound **4-4** (400 MHz, DMSO-d_6)

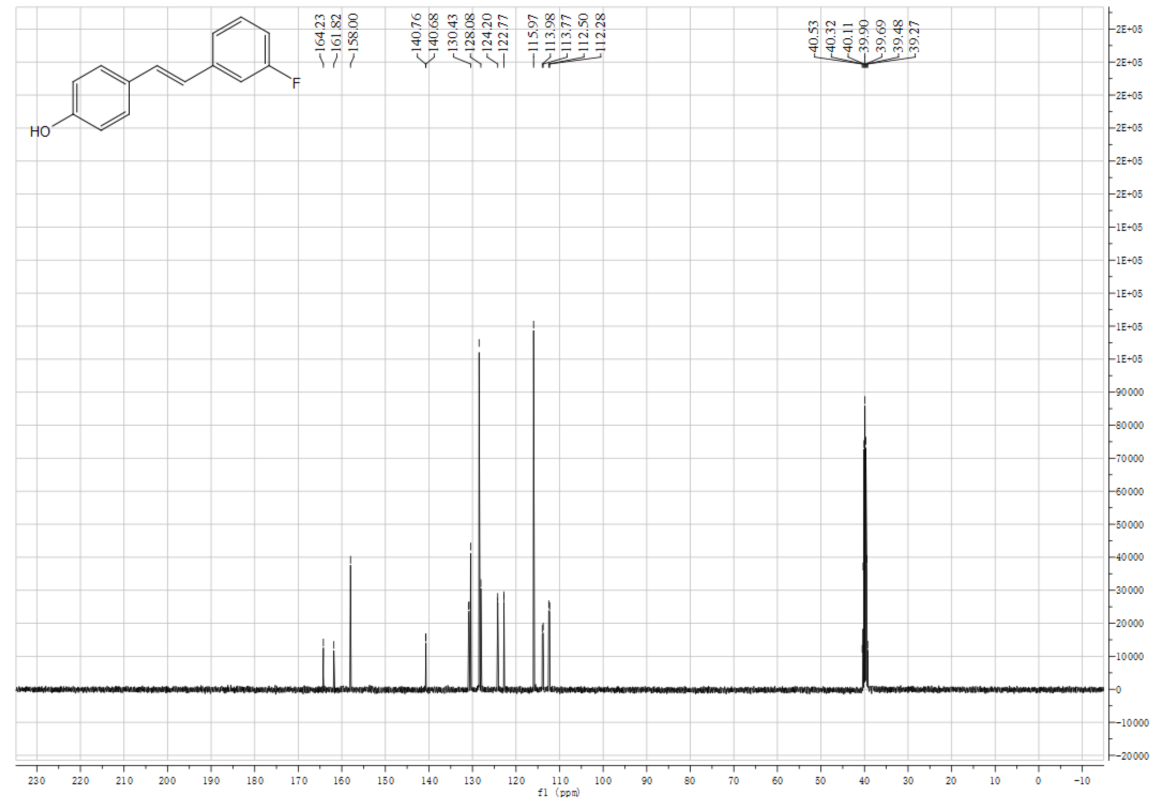

(up) ^{13}C NMR spectrum of compound **4-4** (101 MHz, DMSO-d_6)

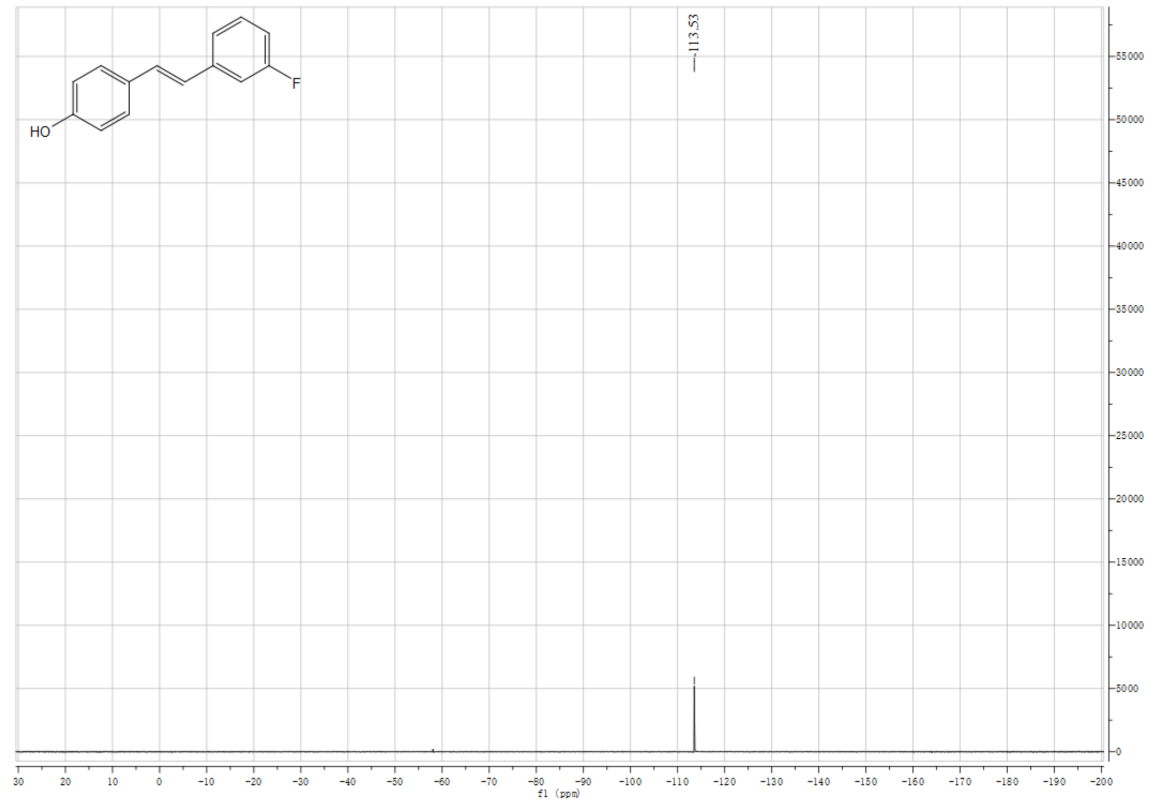

(up) ^{19}F NMR spectrum of compound **4-4** (400 MHz, DMSO-d_6)

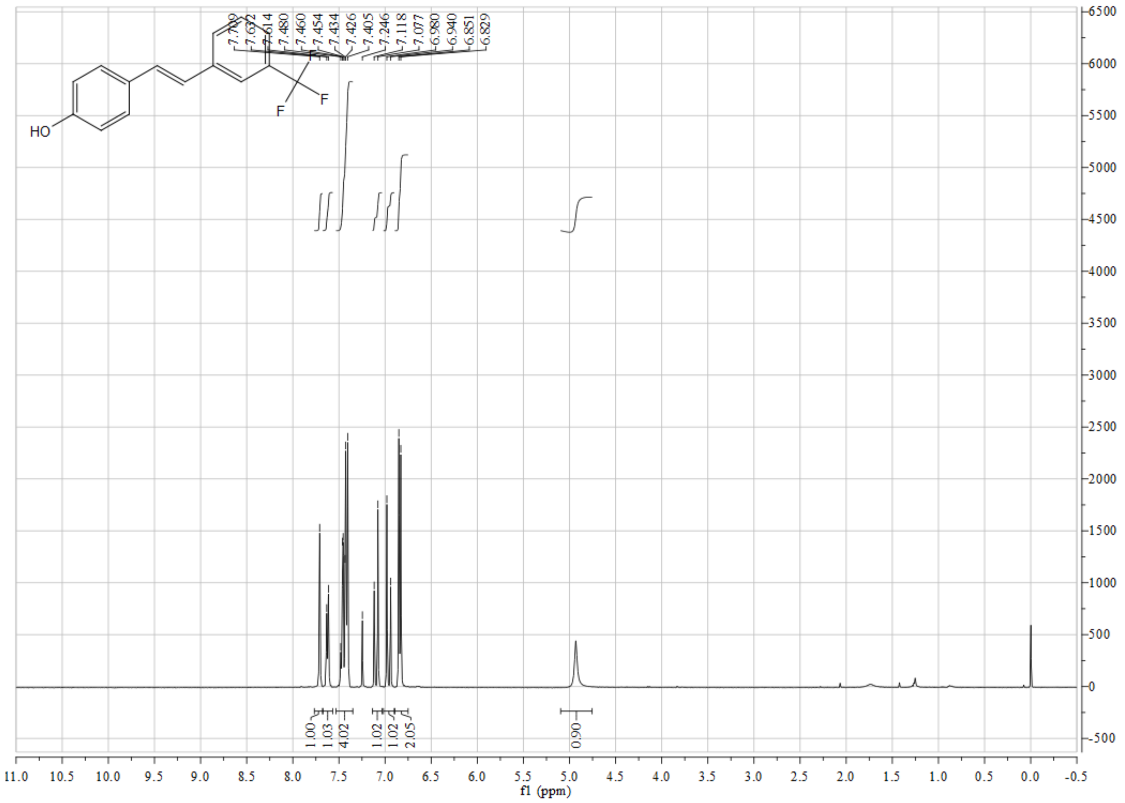

(up) ^{1}H NMR spectrum of compound **4-5** (400 MHz, $CDCl_3$)

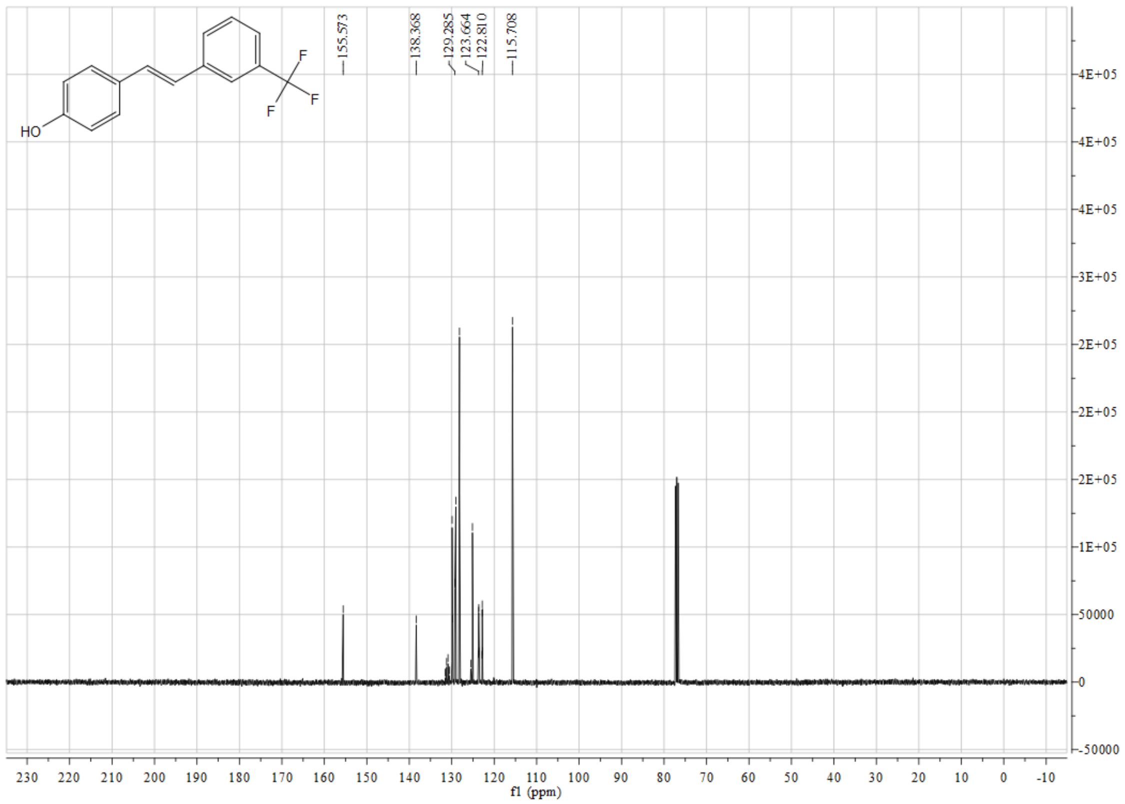

(up) ^{13}C NMR spectrum of compound **4-5** (101 MHz, $CDCl_3$)

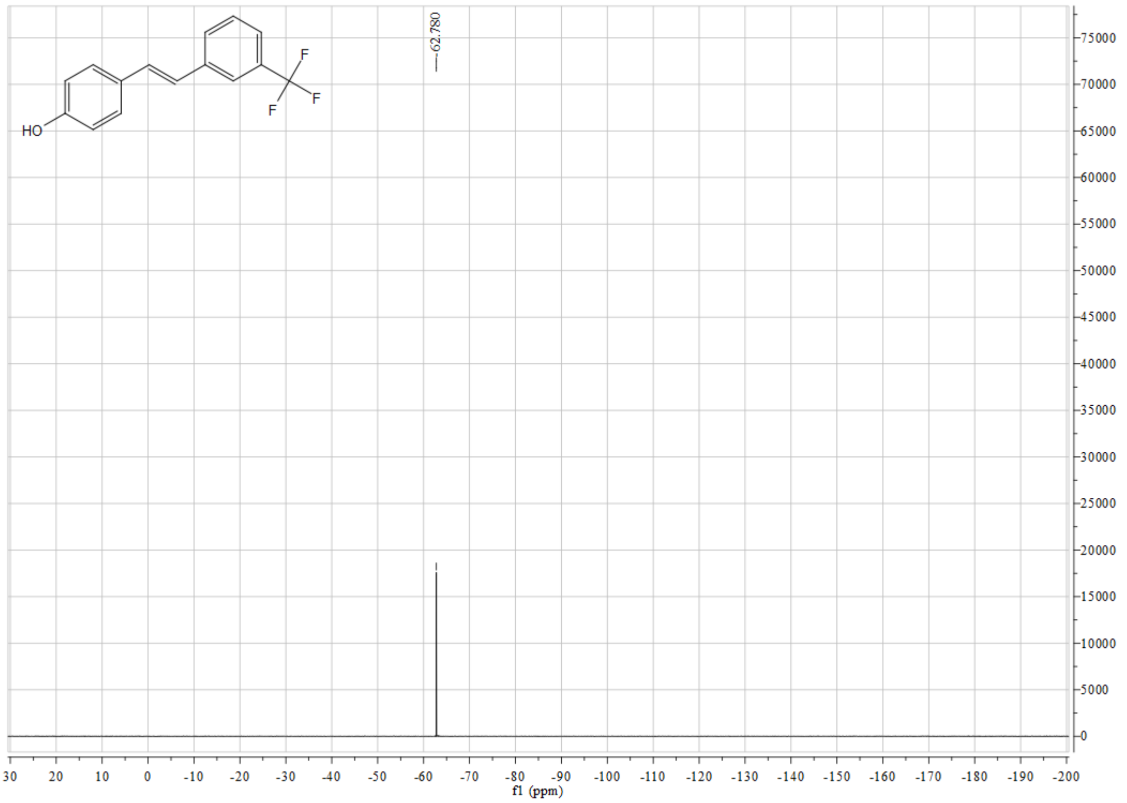

(up) ^{19}F NMR spectrum of compound **4-5** (400 MHz, $CDCl_3$)

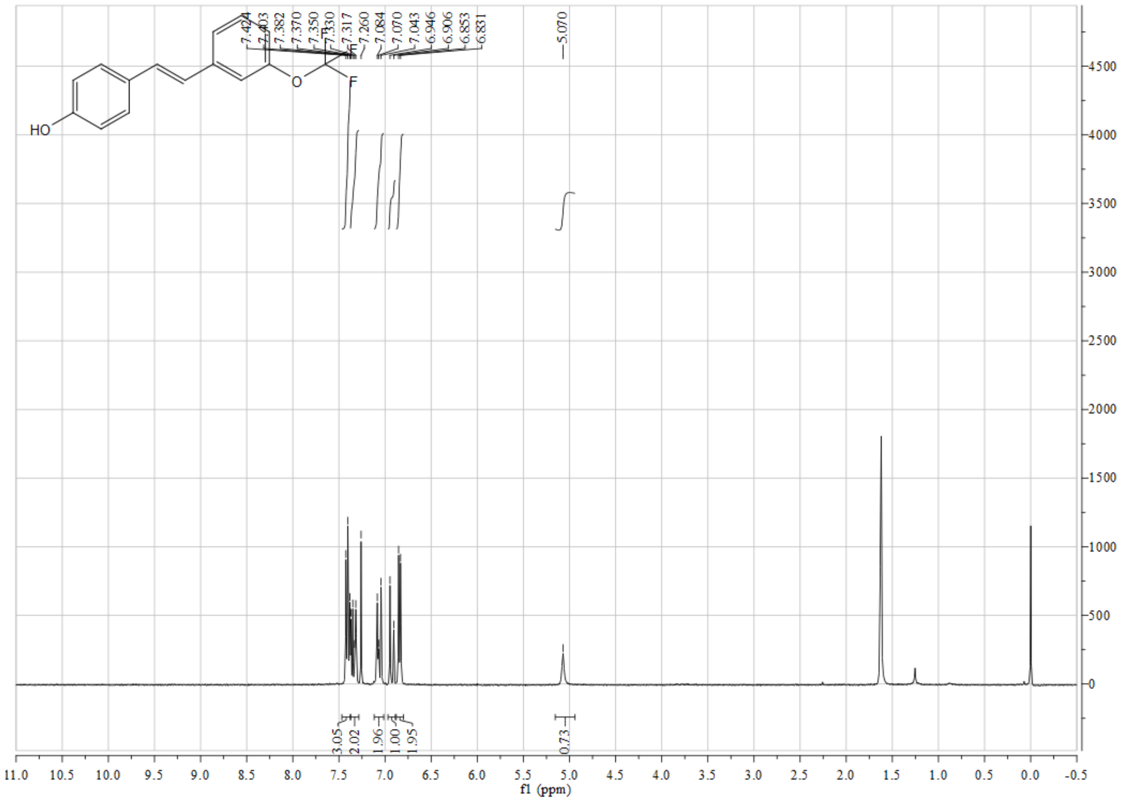

(up) ^{1}H NMR spectrum of compound **4-6** (400 MHz, $CDCl_3$)

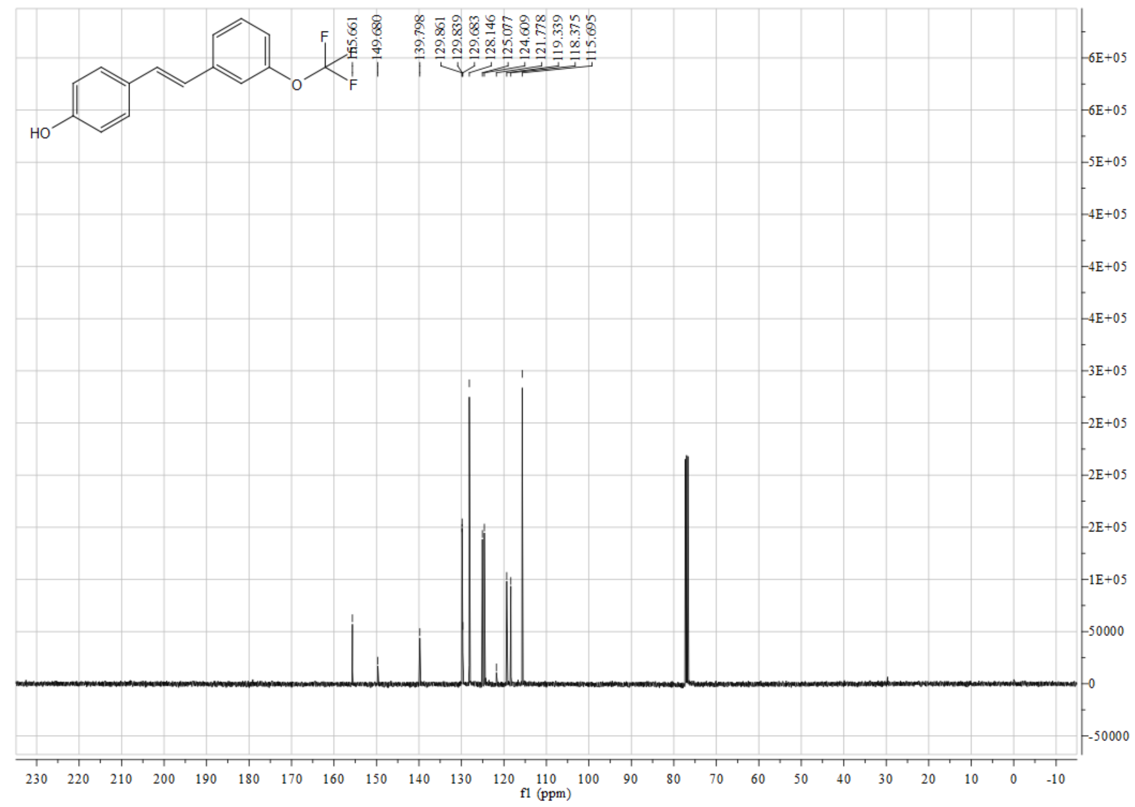

(up) ^{13}C NMR spectrum of compound **4-6** (101 MHz, $CDCl_3$)

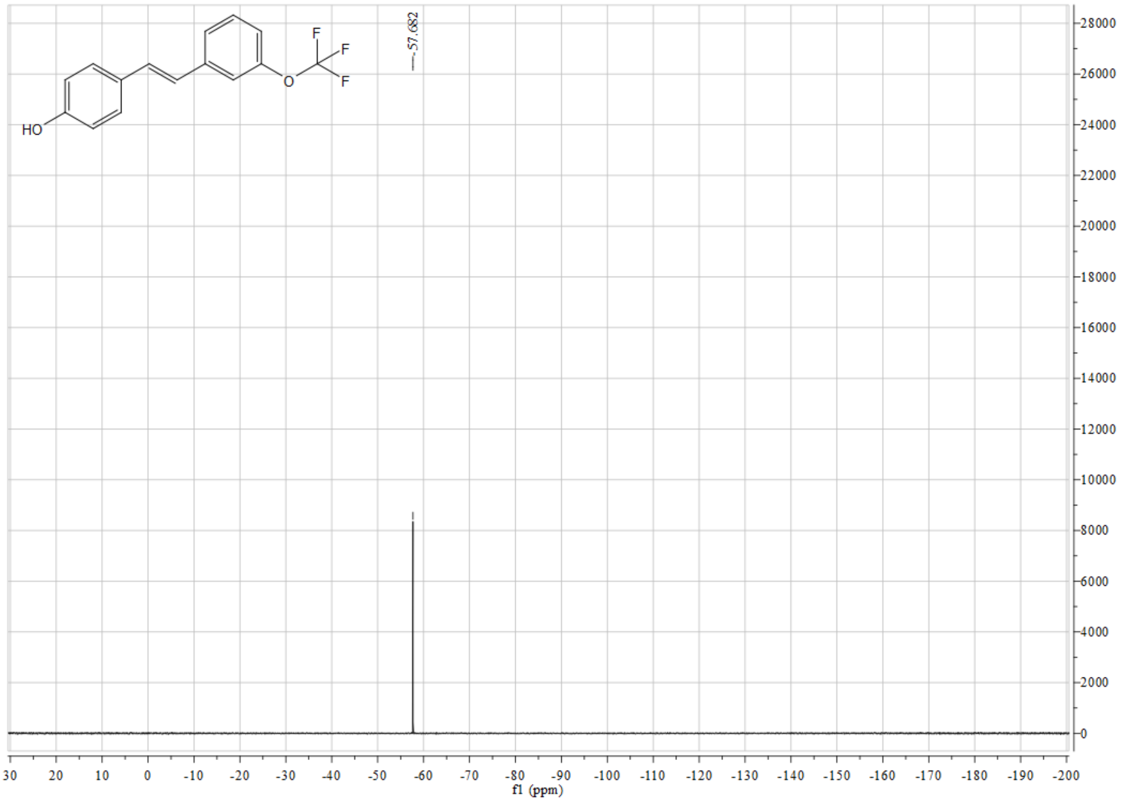

(up) ^{19}F NMR spectrum of compound **4-6** (400 MHz, $CDCl_3$)

4. HPLC spectra resveratrol derivatives

The purity of compounds was checked by HPLC and listed in Table 1 (see below).

Table S1. HPLC analysis data for the tested compounds[a]

Compound	Eluent (water/acetonitrile, v/v)	Retention time (min)	Purity (%)
**1-2**[b]	20:80	3.828	97.66
**1-3**[a]	20:80	4.877	99.82
**1-4**[a]	20:80	3.283	98.78
**2-1**[b]	20:80	2.392	99.25
**2-2**[a]	20:80	3.917	97.58
**2-3**[a]	20:80	4.850	98.90
**2-4**[a]	20:80	5.117	97.02
**2-5**[b]	20:80	3.558	97.66
**3-1**[b]	20:80	2.423	96.85
**3-2**[a]	20:80	3.233	99.29
**3-3**[a]	20:80	3.700	98.73

**3-4**[a]	20:80	3.883	98.99
**3-5**[b]	20:80	2.935	99.96
**6-1**[a]	20:80	4.050	97.03
**6-2**[a]	20:80	4.683	99.68
**6-3**[a]	20:80	4.717	99.43
**6-4**[b]	20:80	3.568	99.83
**6-5**[a]	20:80	4.733	99.61
**6-6**[b]	20:80	4.523	99.98

[a] HPLC analysis was performed using a Persee L600 instrument, with photodiode array detector and a Pgrandsil-STC-C18 (4.6x250 mm, 5 µm particle size, 100 Å). The flow rate was set at 1 mL/min.

[b] HPLC analysis was performed using a UltiMate 3000 instrument, with photodiode array detector and a Eclipse-XDB-C18 (4.6x250 mm, 5 µm particle size, 100 Å). The flow rate was set at 1 mL/min.

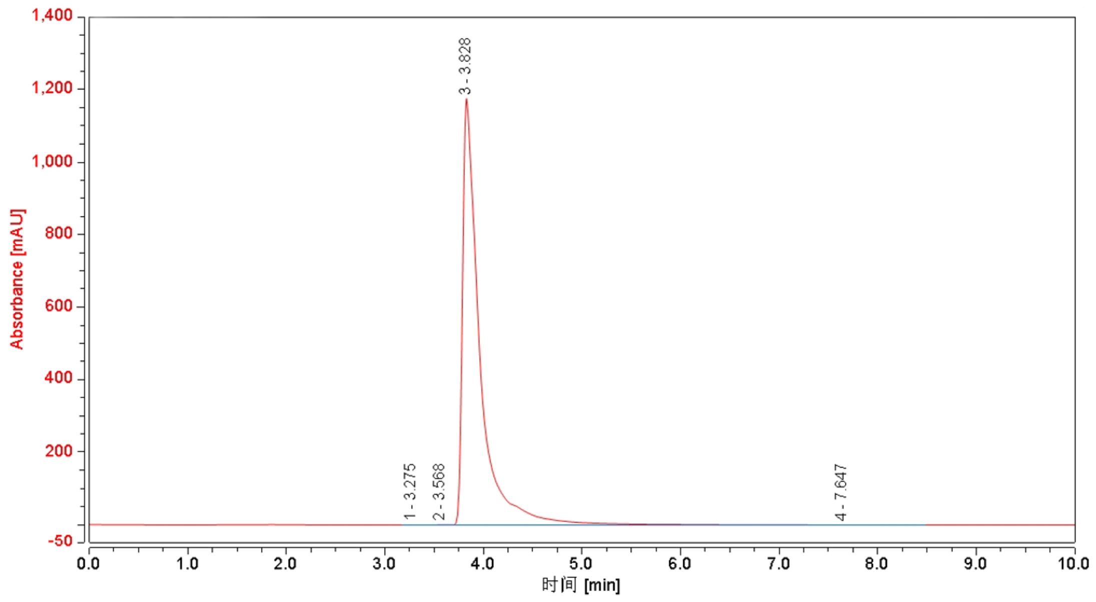

(up) HPLC spectrum of compound **1-2**

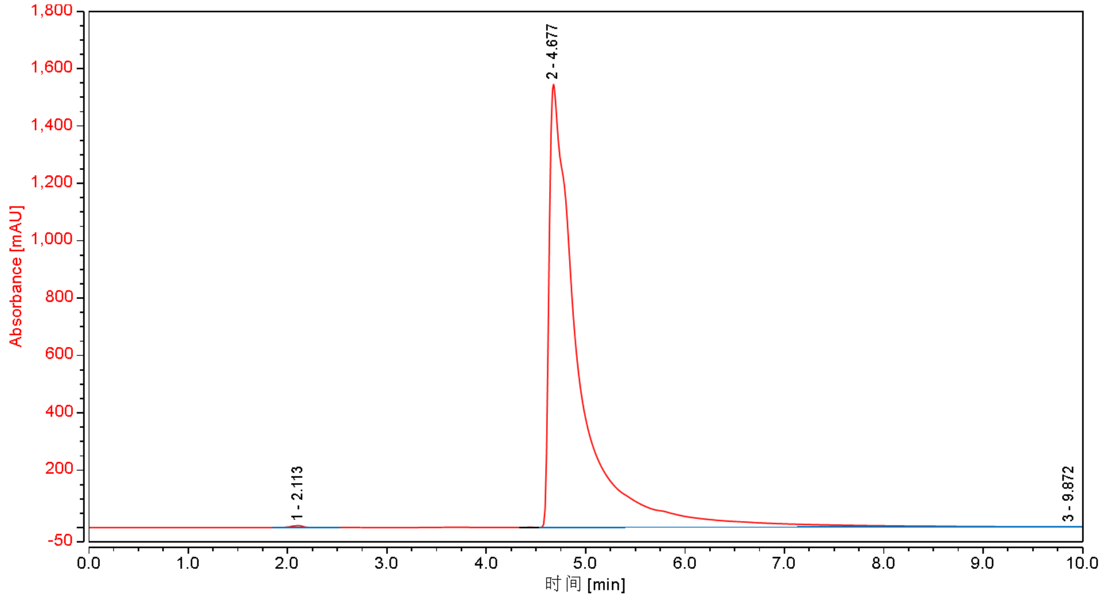

(up) HPLC spectrum of compound **1-3**

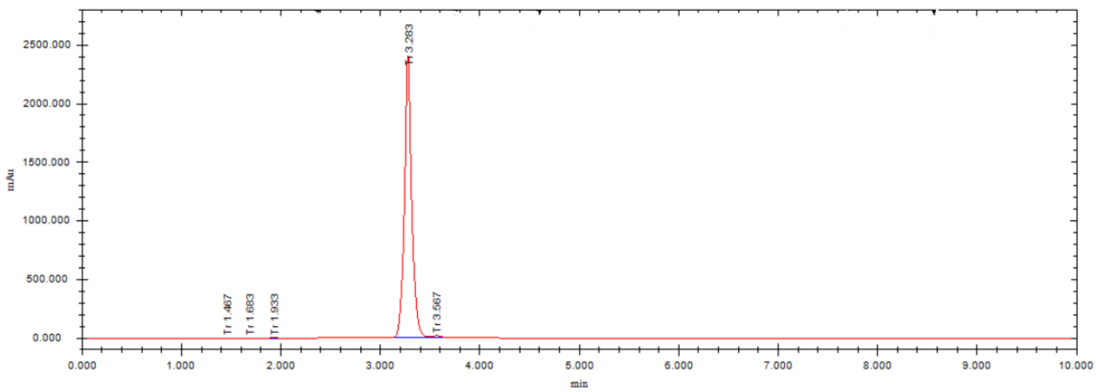

(up) HPLC spectrum of compound **1-4**

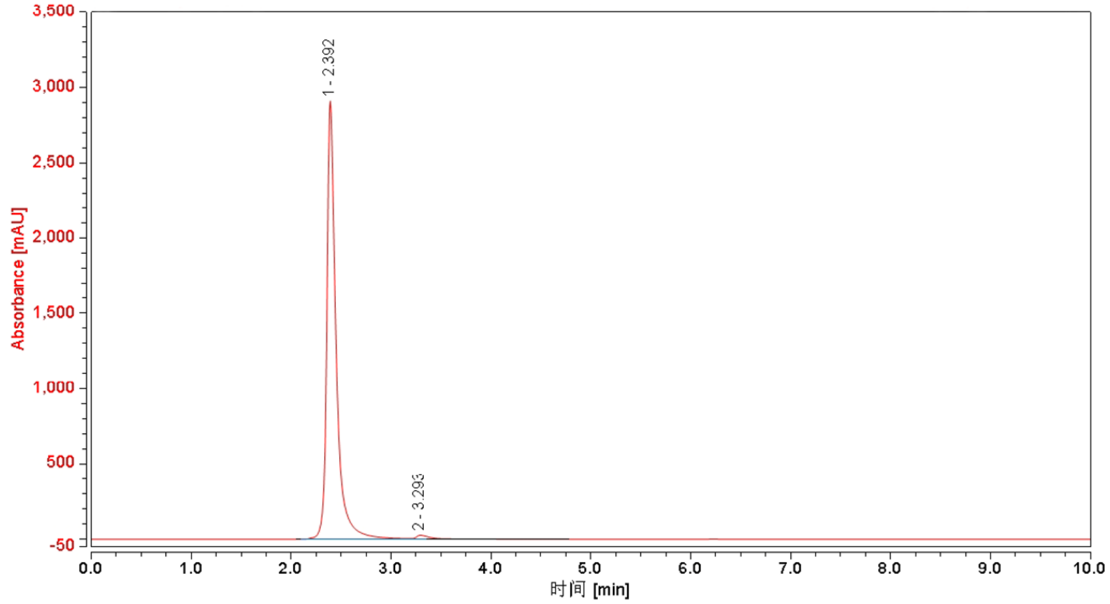

(up) HPLC spectrum of compound **2-1**

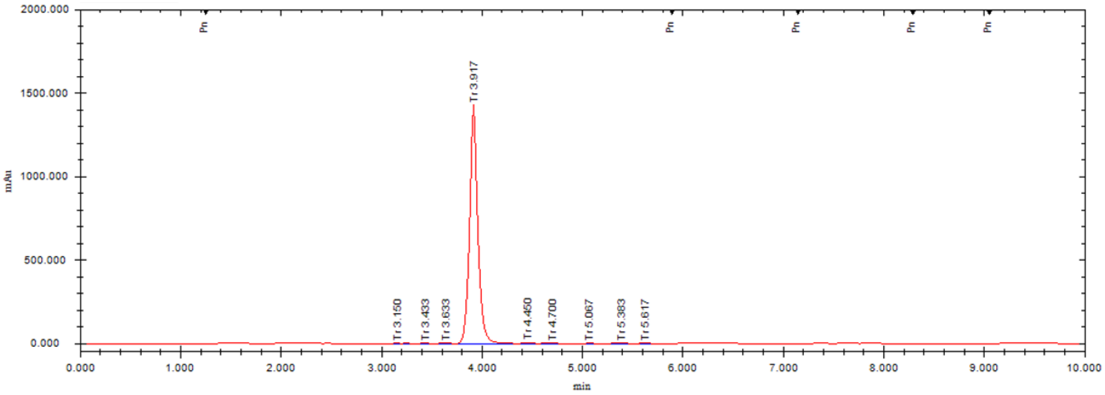

(up) HPLC spectrum of compound **2-2**

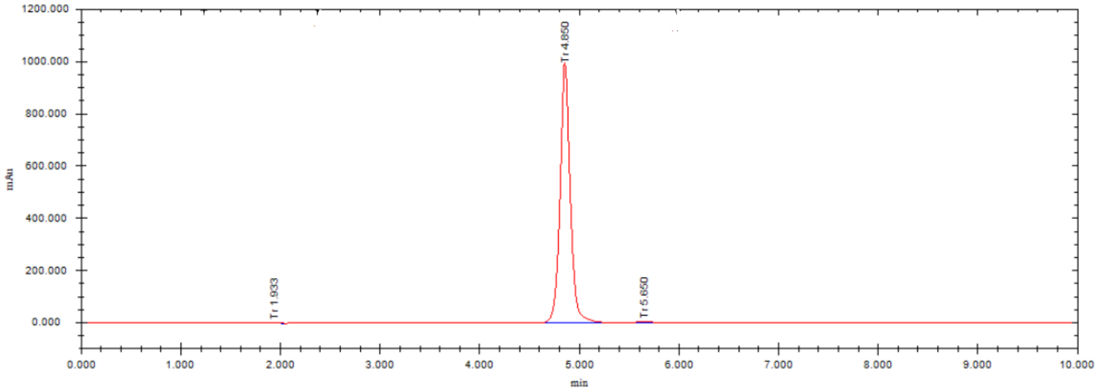

(up) HPLC spectrum of compound **2-3**

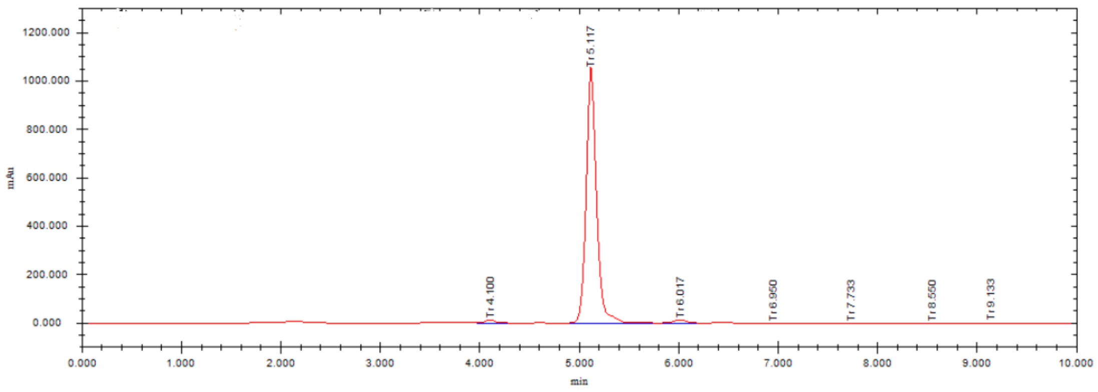

(up) HPLC spectrum of compound **2-4**

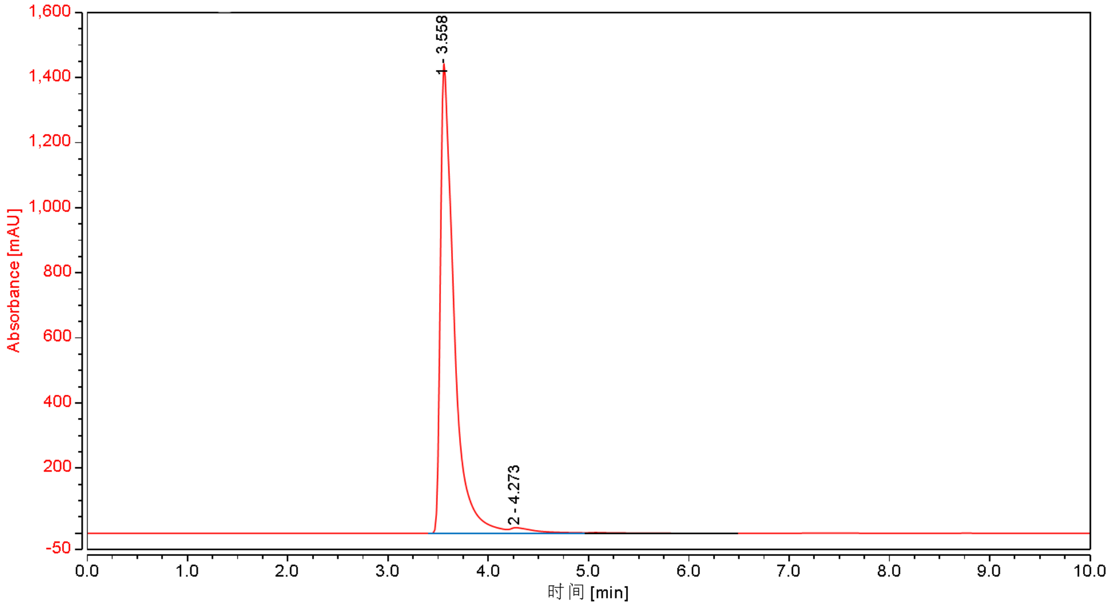

(up) HPLC spectrum of compound **2-5**

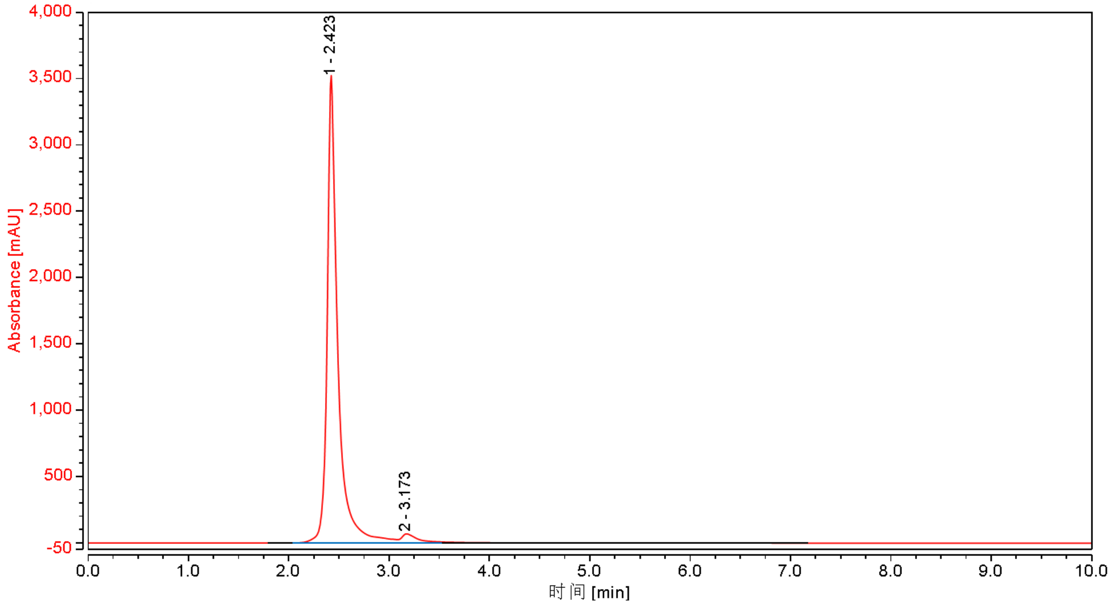

(up) HPLC spectrum of compound **3-1**

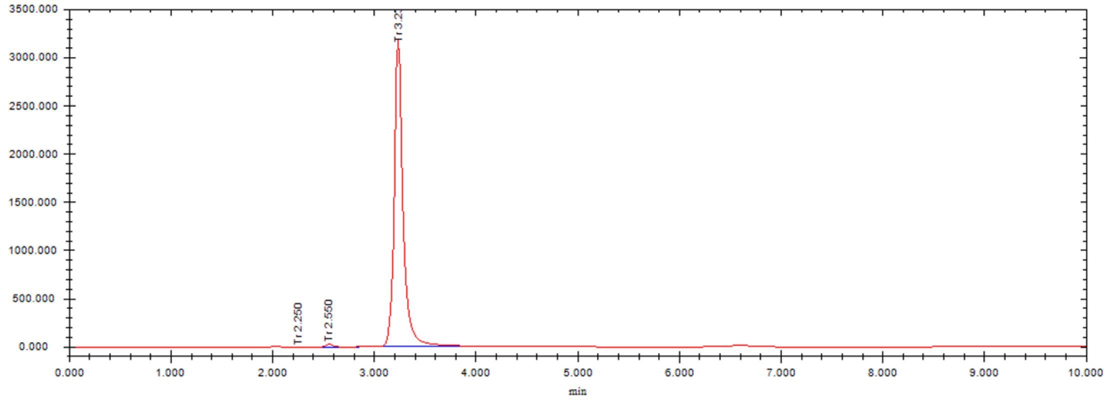

(up) HPLC spectrum of compound **3-2**

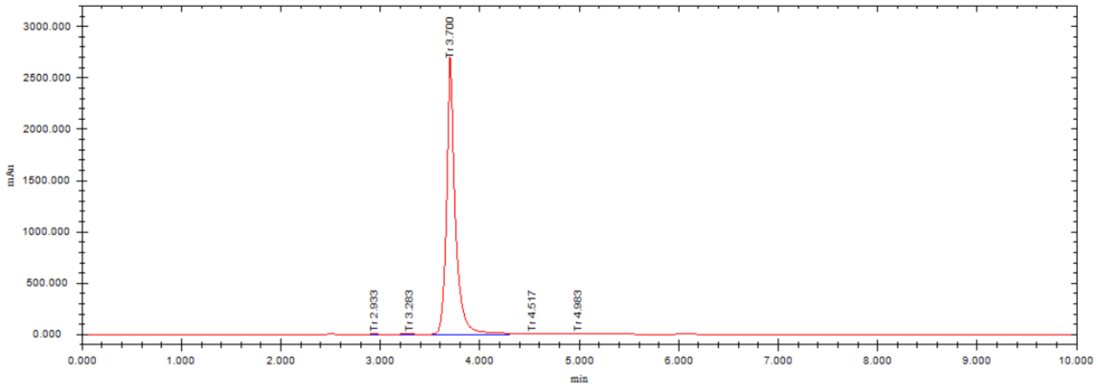

(up) HPLC spectrum of compound **3-3**

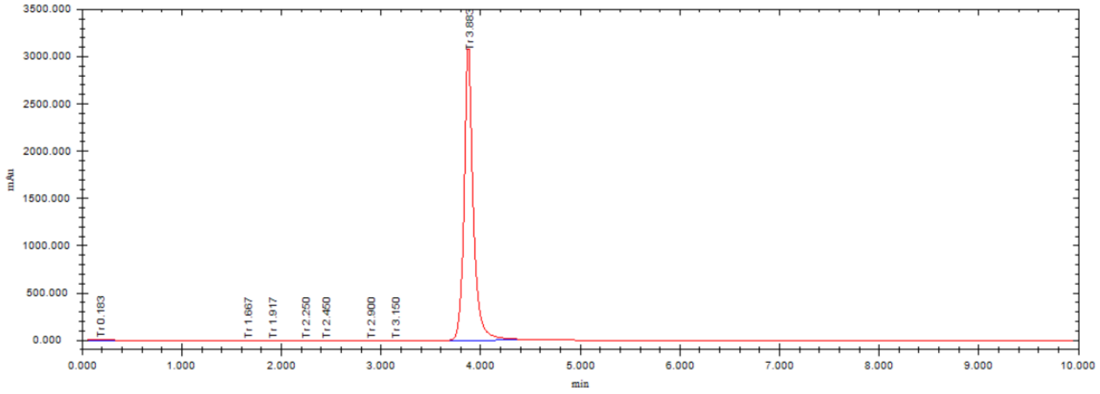

(up) HPLC spectrum of compound **3-4**

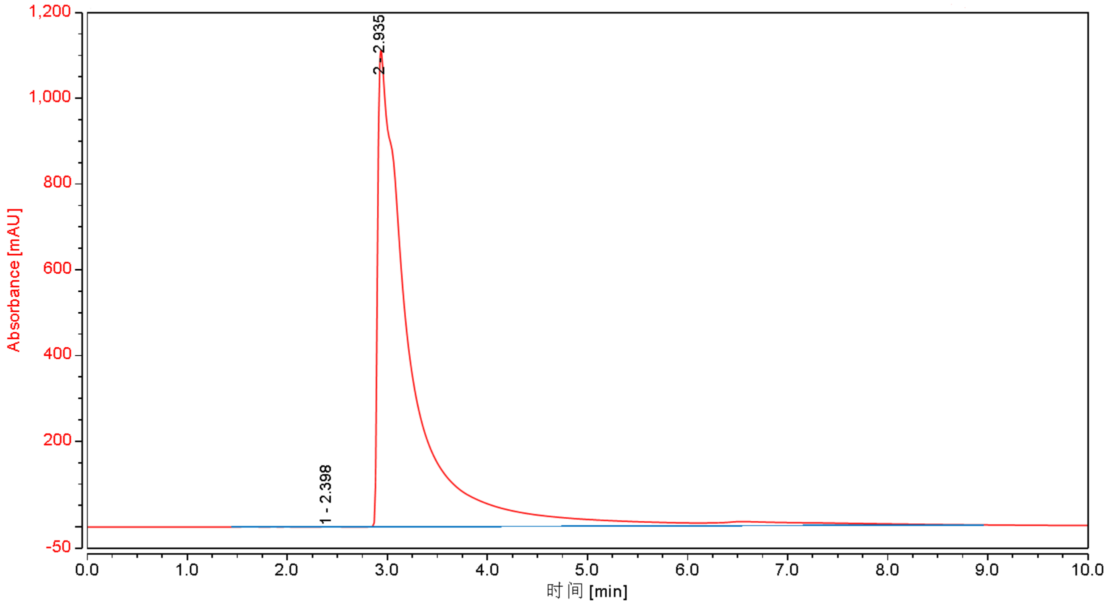

(up) HPLC spectrum of compound **3-5**

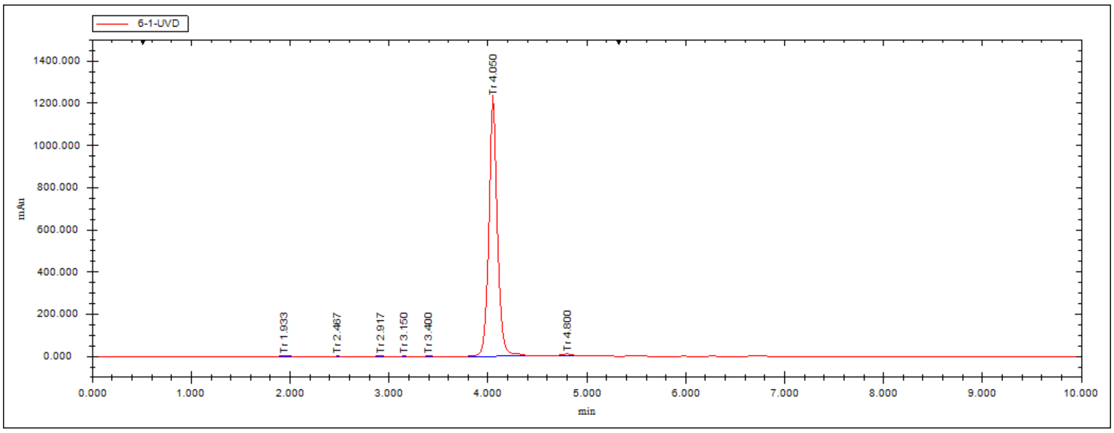

(up) HPLC spectrum of compound **4-1**

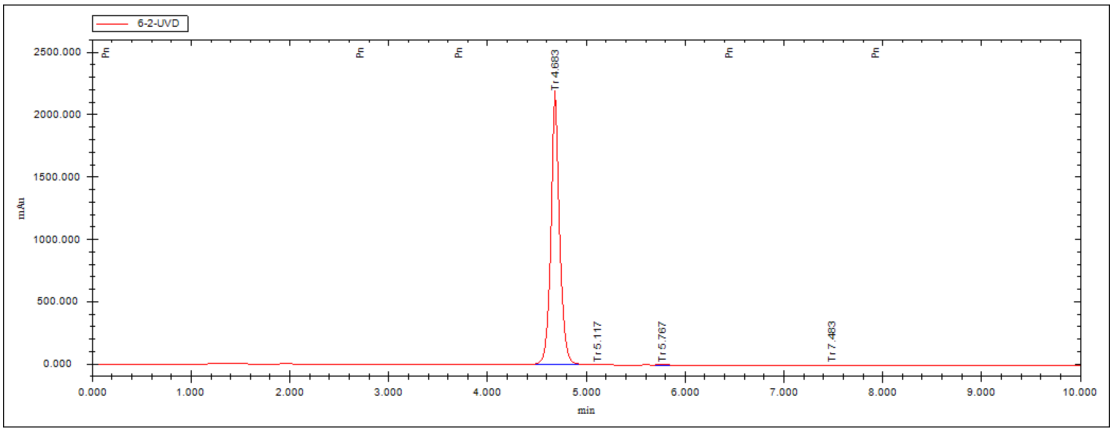

(up) HPLC spectrum of compound **4-2**

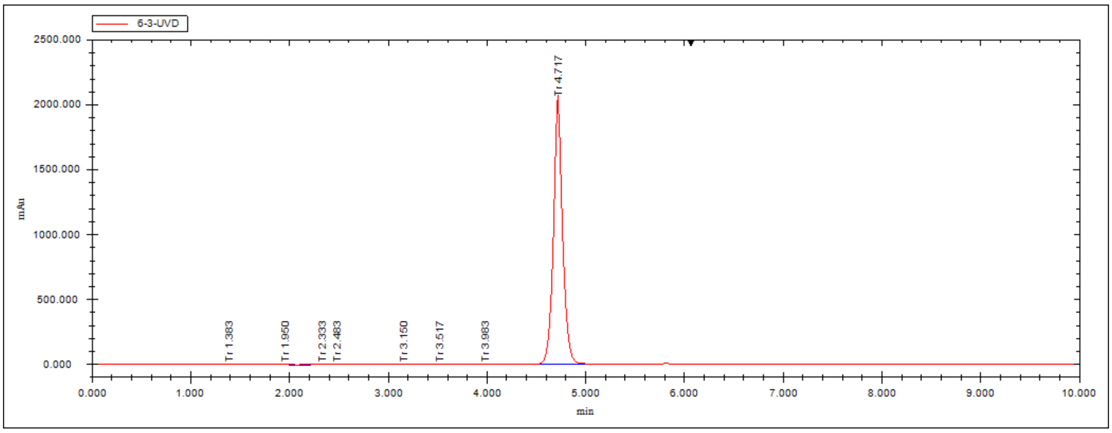

(up) HPLC spectrum of compound **4-3**

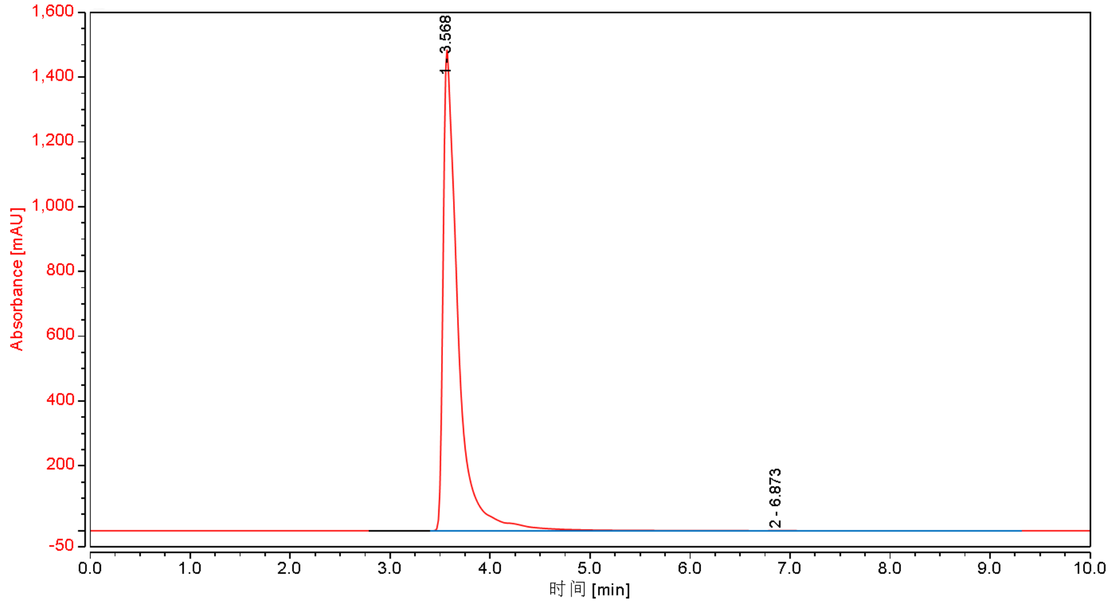

(up) HPLC spectrum of compound **4-4**

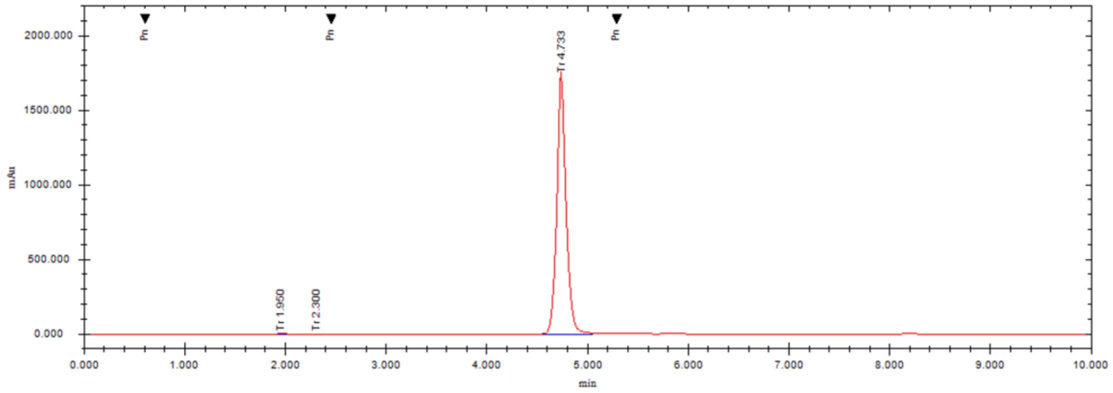

(up) HPLC spectrum of compound **4-5**

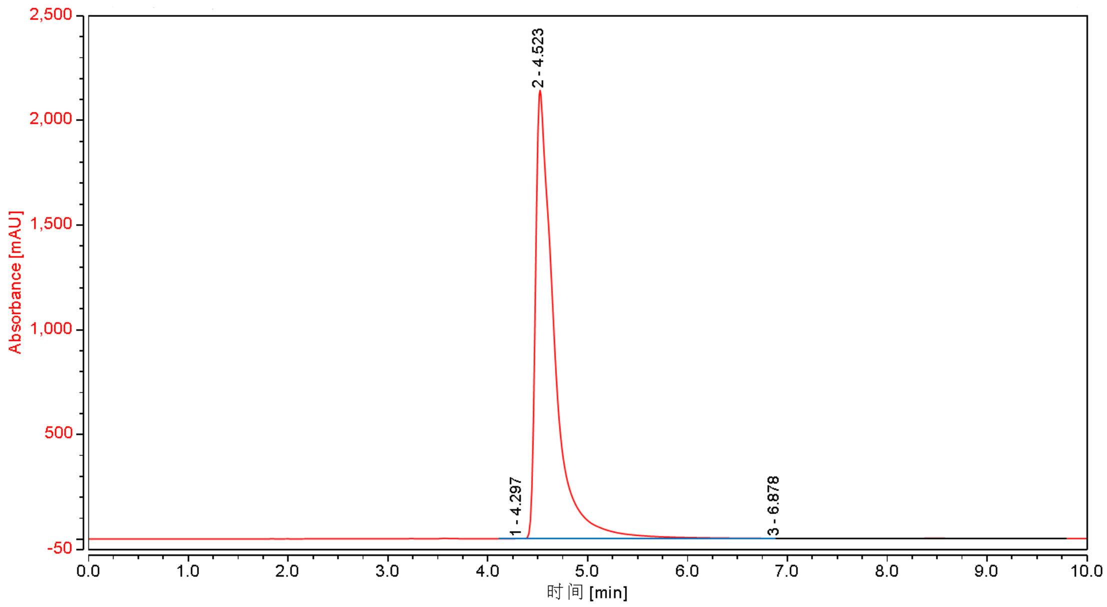

(up) HPLC spectrum of compound **4-6**

References

[1] Yang, J.; Li, J.; Qu, X.Y.; Liu, G.Y.; Liu, R.M. A novel resveratrol derivative induces oxidative stress, G1 cell cycle arrest and premature senescence in A549 cells. *Lat. Am. J. Pharm.* **2019**, *38*, 907-917.

[2] Yang, J.; Liu, F.L.; Cao, Y.X.; Li, Y.R.; Liu, G.Y. 3,5-dihydroxy-4'-fluoro-trans-stilbenzen inhibits the proliferation of A 549 cells via G1 cell cycle arrest and premature senescence. *Lat. Am. J. Phar.* **2019**, *38*, 1838-1845.

[3] Treadwell, E.M., Cermak, S.C., Wiemer, D.F., Synthesis of schweinfurthin C, a geranylated stilbene from macaranga schweinfurthii, J. Org. Chem. **1999**, *64*, 8718-23.

[4] Yerien, D.E., S. Bonesi, S., Postigo, A., Fluorination methods in drug discovery, Org. Biomol. Chem. **2016**, *14*, 8398-8427.

[5] Lee, I., Choe, Y.S., Choi, J.Y., Lee, K.H., Kim, B.T., Synthesis and Evaluation of18F-Labeled Styryltriazole andResveratrol Derivatives forβ-Amyloid Plaque Imaging, J. Med. Chem. **2012**, *55*, 883-892.

[6] Kang, S.S., M. Cuendet, D.C. Endringer, V.L. Croy, J.M. Pezzuto & M.A., Synthesis and biological evaluation of a library of resveratrol analogues as inhibitors of COX-1, COX-2 and NF-κB. Bioorgan. Med. Chem. **2009**,*17*, 1044-54.

[7] Yang, J., Liu, G.Y., Chen, J.Z., Guo, S.J., Resveratrol derivative containing fluorine group, and preparation method and application, P., CN,**2017**,107011127A.

[8] Shang, Y.J., Qian, Y.P., Liu, X.D., Dai, F., Shang, X.L., Jia, W.Q., Liu, Q., Fang, J.G., Zhou, B., Radical-scavenging activity and mechanism of resveratrol-oriented analogues: influence of the solvent, radical, and substitution, J. Org. Chem. **2009**, *74*, 5025-5031.

[9] Lion, C.J., Matthews, C.S., Stevens, M.F.G., Westwell, A.D., Synthesis, Antitumor Evaluation,

and Apoptosis-Inducing Activity ofHydroxylated (E)-Stilbenes, J. Med. Chem. **2005**, *48*, 1292-1295.
[10] Chan, S.Y., Loh, Y.C., Oo, C.W., Yam, M.F., *In vitro* study and structure-activity relationship analysis of stilbenoid derivatives as powerful vasorelaxants: Discovery of new lead compound, Bioorg. Chem. **2020**, *104*, 104239.
[11] Wang, B., Zhang, Y.K., Chen, J., Zhang, W., Song, J.G., Balzo, U.D., Brown, L., Boddupalli, S., Bobzin, S., Gilat, S., Miller, G., Cytoprotective compounds, pharmaceutical and cosmetic formulations, and methods, P., US,**2003**,73712.
[12] Zhang, Y., Wang, Q., Li, L.L., Le, Y., Liu, L., Yang, J., Li, Y.L., Bao, G.C., Yan, L.J., Synthesis and preliminary structure-activity relationship study of3-methylquinazolinone derivatives as EGFR inhibitors with enhancedantiproliferative activities against tumour cells, J. of Enzyme Inhibiton and Medicinal Chemistry, **2021**, *36*, 1205-1216.
[13] Bavo, F., Pallavicini, M., Pucci, S., Appiani, R., Giraudo, A., Oh, H., Kneisley, D.L., Eaton, B., Lucero, L., Gotti, C., Clementi, F., Whiteaker, P., Bolchi, C., Subnanomolar affinity and selective antagonism at α7 nicotinic receptor by combined modifications of 2-triethylammonium ethyl ether of 4-Stilbenol (MG624), J. Med. Chem. **2023**, *66*, 306-332.
[14] Liang, J.H., Yang, L., Wu, S., Liu, S.S., Cushman, M., Tian, J., Li, N.M., Yang, Q.H., Zhang, H.A., Qiu, Y.J., Xiang, L., Ma, C.X., Li, X.M., Qing, H., Discovery of efficient stimulators for adult hippocampal neurogenesis based on scaffolds in dragon's blood, Eur. J. Med. Chem. **2017**, *136*, 382-392.
